# A pilot study evaluating GSK1070806 inhibition of interleukin-18 in renal transplant delayed graft function

**DOI:** 10.1371/journal.pone.0247972

**Published:** 2021-03-08

**Authors:** E. Wlodek, R. B. Kirkpatrick, S. Andrews, R. Noble, R. Schroyer, J. Scott, C. J. E. Watson, M. Clatworthy, E. M. Harrison, S. J. Wigmore, K. Stevenson, D. Kingsmore, N. S. Sheerin, O. Bestard, H. A. Stirnadel-Farrant, L. Abberley, M. Busz, S. DeWall, M. Birchler, D. Krull, K. S. Thorneloe, A. Weber, L. Devey

**Affiliations:** 1 GlaxoSmithKline, Clinical Unit Cambridge, Addenbrooke’s Hospital, Cambridge, United Kingdom; 2 Department of Medicine, University of Cambridge, Cambridge, United Kingdom; 3 GlaxoSmithKline, Philadelphia, Pennsylvania, United States of America; 4 JMS Statistics Ltd, Pinner, United Kingdom; 5 University of Cambridge and the NIHR Cambridge Biomedical Research Centre and the NIHR Blood and Transplant Research Unit in Organ Donation and Transplantation at the University of Cambridge, Cambridge, United Kingdom; 6 Royal Infirmary of Edinburgh, Edinburgh, United Kingdom; 7 Queen Elizabeth University Hospital, Glasgow, United Kingdom; 8 Newcastle Biomedical Research Centre and the NIHR Blood and Transplant Research Unit in Organ Donation and Transplantation, Newcastle University, Newcastle, United Kingdom; 9 L’Hospitalet de Llobregat, Bellvitge University Hospital, Kidney Transplant Unit, Barcelona, Spain; 10 GlaxoSmithKline, R&D, Stevenage, United Kingdom; Medizinische Universitat Graz, AUSTRIA

## Abstract

**Introduction:**

Delayed graft function (DGF) following renal transplantation is a manifestation of acute kidney injury (AKI) leading to poor long-term outcome. Current treatments have limited effectiveness in preventing DGF. Interleukin-18 (IL18), a biomarker of AKI, induces interferon-γ expression and immune activation. GSK1070806, an anti-IL18 monoclonal antibody, neutralizes activated (mature) IL18 released from damaged cells following inflammasome activation. This phase IIa, single-arm trial assessed the effect of a single dose of GSK1070806 on DGF occurrence post donation after circulatory death (DCD) kidney transplantation.

**Methods:**

The 3 mg/kg intravenous dose was selected based on prior studies and physiologically based pharmacokinetic (PBPK) modeling, indicating the high likelihood of a rapid and high level of IL18 target engagement when administered prior to kidney allograft reperfusion. Utilization of a Bayesian sequential design with a background standard-of-care DGF rate of 50% based on literature, and confirmed via extensive registry data analyses, enabled a statistical efficacy assessment with a minimal sample size. The primary endpoint was DGF frequency, defined as dialysis requirement ≤7 days post transplantation (except for hyperkalemia). Secondary endpoints included safety, pharmacokinetics and pharmacodynamic biomarkers.

**Results:**

GSK1070806 administration was associated with IL18-GSK1070806 complex detection and increased total serum IL18 levels due to IL18 half-life prolongation induced by GSK1070806 binding. Interferon-γ−induced chemokine levels declined or remained unchanged in most patients. Although the study was concluded prior to the Bayesian-defined stopping point, 4/7 enrolled patients (57%) had DGF, exceeding the 50% standard-of-care rate, and an additional two patients, although not reaching the protocol-defined DGF definition, demonstrated poor graft function. Six of seven patients experienced serious adverse events (SAEs), including two treatment-related SAEs.

**Conclusion:**

Overall, using a Bayesian design and extensive PBPK dose modeling with only a small sample size, it was deemed unlikely that GSK1070806 would be efficacious in preventing DGF in the enrolled DCD transplant population.

**Trial registration:**

NCT02723786.

## Introduction

Acute kidney injury (AKI) complicates 8–18% of hospital admissions, and an increasing body of evidence shows that these acute insults are associated with short- and long-term increases in healthcare cost, morbidity and mortality [1–3]. Delayed graft function (DGF), a consequence of AKI, is defined as the failure of a transplanted kidney to function immediately after transplantation [1], and is associated with a long-term increased risk of kidney graft failure [2]. The rate of DGF in kidney transplant recipients varies between approximately 25% and 50% depending on the presence of risk factors, including donor age, cold ischemic time, and the cause of donor death [3, 4]. DGF is thought to occur as a result of ischemic renal injury associated with cellular hypoxia (depletion of adenosine triphosphate, loss of membrane potentials) and subsequent reperfusion, which initiates inflammatory cascades (formation of reactive oxygen species, immune cell stimulation, cytokine release and complement activation) [5–7]. The inflammasome can also be activated, leading to production of the inflammatory cytokine interleukin-18 (IL18) that is thought to contribute to the pathogenesis of AKI [8]. IL18, acting on its receptors, then induces synthesis of proinflammatory cytokines, including interferon-γ (IFNγ), thereby mediating inflammatory responses [9–12].

Clinical studies have shown that increases in urinary IL18 levels within the first few hours after cardiac surgery or kidney transplantation are associated with a higher risk of AKI development in native [13] and transplanted [14] kidneys, respectively. Furthermore, elevated urinary IL18 levels are associated with long-term allograft dysfunction and mortality [15, 16]. More causal evidence of the role of IL18 in AKI comes from experimental rodent models of renal ischemic injury, in which IL18 neutralization and gene deletion are protective [17–20]. IL18 induction may also play a role in acute rejection, as suggested by data showing that elevated levels of the IFNγ-inducible chemokines, IFNγ-induced protein 10 kD (IP10) and monokine induced by IFNγ (MIG), are predictive of acute rejection episodes [21–24]. Indeed, IL18 was constitutively expressed in renal biopsies and serum IL18 was elevated in patients experiencing acute allograft rejection [25].

Although machine perfusion or cooling of the donor have been suggested to provided reductions in the rate of DGF [26, 27], there are no approved medicines for DGF treatment or prevention [28] and the rate of DGF following kidney transplant remains high at 25–50% [3, 4]. As such, development of new therapies for the prevention of DGF remains a key unmet need. GSK1070806 is a humanized immunoglobulin (Ig) G1 antibody that binds with high affinity (Kd 46.0 pM) to neutralize the function of mature IL18 [29]. A single infusion of GSK1070806 was well tolerated up to the highest doses tested (10 mg/kg in healthy individuals and 3 mg/kg in obese individuals), where GSK1070806 treatment inhibited ex vivo stimulation of IFNγ production and natural killer surface marker expression in whole blood assays [29]. GSK1070806 was also well tolerated in a phase II study in patients with type 2 diabetes, in which two doses of GSK1070806 were administered 28 days apart [30].

Here we used a Bayesian sequential statistical methodology, in conjunction with a comprehensive contemporary renal transplantation dataset from the UK Transplant Registry, to design a small, single-arm study evaluating the potential for the anti-IL18 monoclonal antibody GSK1070806 to reduce DGF in donation after circulatory death (DCD) transplanted kidneys. Additionally, using a three-pillar approach [31], we have applied detailed physiologically-based pharmacokinetic (PBPK) modeling with associated sensitivity analyses to predict GSK1070806 plasma and tissue exposure, and the extent of target engagement at the sites of action considered relevant for the contribution of IL18 to DGF.

## Materials and methods

### Study design

This was a phase IIa, multicenter, single-arm Bayesian sequential design, pilot study to evaluate the efficacy, safety, tolerability, and PK of GSK1070806 in patients undergoing renal transplantation (GSK study 204824; ClinicalTrials.gov Identifier NCT02723786 https://clinicaltrials.gov/ct2/show/NCT02723786). The trial was conducted between August 2016 and March 2018.

**Fig 1:**
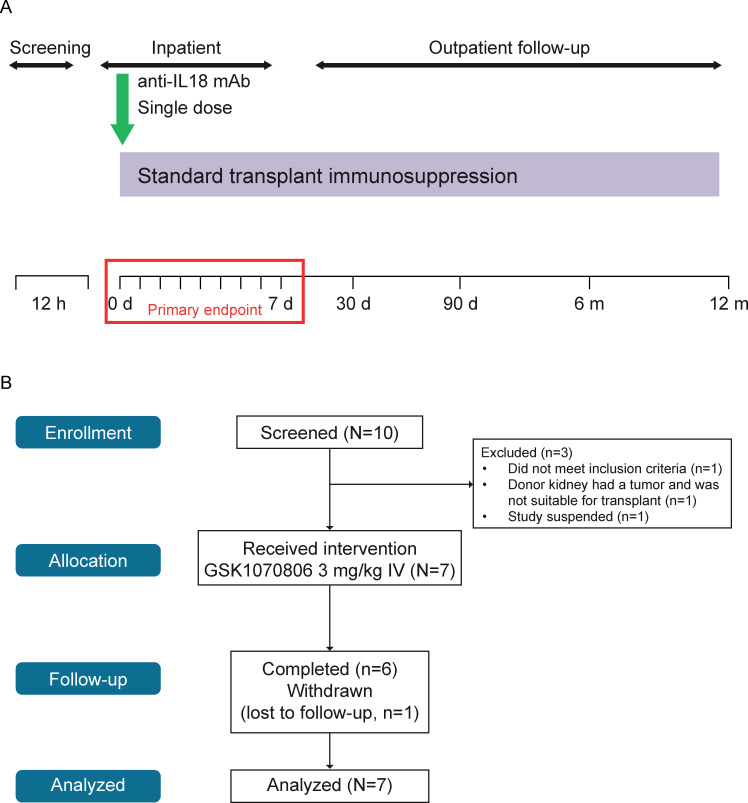
(A) Study design and (B) CONSORT flow diagram. d, day; aIL18, anti-interleukin-18; m, month; mAb, monoclonal antibody.

The trial included a screening period (at patient presentation to hospital), an inpatient period (including pre-operative, intra-operative, and in-hospital recovery periods) and a follow-up period commencing 30 days post transplantation with visits on Day 30, Day 90, Month 6, and Month 12 (**Fig 1A**). Transplantation took place on Day 0. Withdrawal and stopping criteria are described in **S1 Supplementary Material in S1 File**.

A total of 9 centers in 2 countries (hospitals in the UK and Spain) were initiated to enroll participants. Patients were identified/recruited by investigators and data collected at the study sites. The study protocol, amendments, and informed consent form were approved by relevant ethics committees or institutional review boards (the Scotland A Research Ethics Committee; the NHS; Bellvitge University Hospital Clinical Research Ethics Committee). This study was conducted in accordance with International Conference on Harmonisation of Technical Requirements for Registration of Pharmaceuticals for Human Use, Good Clinical Practice, and the ethical principles outlined in the Declaration of Helsinki 2008. Written informed consent was obtained for each participant prior to the performance of any study-specific procedures. None of the organ donors were from a vulnerable population and all donors or next of kin provided written informed consent that was freely given.

### Study endpoints

The primary endpoint was incidence of DGF, defined by a requirement for dialysis within the first 7 days post transplantation (except for hyperkalemia during the first 24 hours). Secondary endpoints included assessments of graft function/survival (as measured by serum creatinine, urine output, dialysis events), acute rejection (biopsy-proven, rejection biomarkers), safety and tolerability of GSK1070806 in renal transplantation, and GSK1070806 PK/pharmacodynamics (PD).

### Study population

Study participants were adult (≥18 years of age), dialysis-dependent recipients of first- or second-time single kidney DCD transplants, with immunosuppressive standard-of-care comprised of either: basiliximab and mycophenolate mofetil; or azathioprine, tacrolimus, and corticosteroids.

Patients were excluded from the study for influenza-like illness; if hospitalized or treated in the past 30 days with parenteral antibiotics; diagnosis of hepatitis B virus infection, hepatitis C virus infection, human immunodeficiency virus infection, or tuberculosis; or if they had received live vaccinations or biological immunosuppression within 30 days prior to dosing. Anti-infective prophylaxis therapy and viral testing were implemented to mitigate the theoretical elevated infection risk due to administration of the additional anti-IL18 immunosuppressive GSK1070806 (**S2 Supplementary Material in S1 File**). Recipients with previous organ transplantation (except first kidney or corneal transplants), malignancy in the past 5 years, immunodeficiency, liver function tests where alanine aminotransferase >2x the upper limit of normal and bilirubin >1.5x the upper limit of normal, or an electrocardiogram with QTc > 480 msec were excluded. Patients were excluded, at the discretion of the investigator, if patients had concurrent conditions deemed to pose an unacceptable risk. Full exclusion criteria are listed in **S3 Supplementary Material in S1 File**.

Donor allograft exclusions included cold ischemic time >36 hours; age <5 years old; serology positive for hepatitis B, hepatitis C, or human immunodeficiency virus; ABO blood type incompatibility; and T- and/or B-cell positive crossmatch against the recipient. Full exclusion criteria are listed in **S3 Supplementary Material in S1 File**.

### Dosing

Study participants received a single intravenous (IV) injection of GSK1070806 (3 mg/kg; 100 mg/mL IV solution) administered prior to kidney allograft reperfusion by authorized staff at each study site (**Fig 1**). This dose was selected based on PBPK modeling and prior clinical PK data (**S4 Supplementary Material, S1–S3 Figs and S1–S3 Tables in S1 File**) and was anticipated to inhibit IL18 (>90% target engagement) rapidly (1–2 hours post dose) in both the interstitial space of the kidney and the circulating plasma. Furthermore, the use of the 3 mg/kg dose was well supported by the observed safety data generated in prior clinical studies where 36 patients had received ≥3 mg/kg [29, 30]. GSK1070806 is not currently commercially available and remains under investigational development. However, data access requests and research proposals for GSK1070806 from independent investigators can be submitted to www.clinicalstudydatarequest.com and http://iss.gsk.com.

### Statistical analyses and sample size

The DGF rate in DCD transplanted kidneys was established previously at approximately 50% [3] and this was confirmed with contemporary registry data held by the UK National Health Service Blood and Transplant. Further details are provided in **S5 Supplementary Material in S1 File**.

A Bayesian sequential analysis of efficacy data was planned to allow for the possibility of stopping early for success or failure in this single-arm study. The statistical impact of various sample sizes on type I error rates was explored for a background DGF rate of 50%, along with the power for detecting a targeted 35% DGF rate with the GSK1070806 experimental therapy. This represents a 30% relative reduction in DGF from the 50% background rate (**S5 Table in S1 File**), is deemed to be clinically meaningful and predictive of a significant impact on clinical outcomes in any subsequent larger, late-stage clinical trials. Given the desire to minimize the number of patients in this proof-of-concept study, it was planned with a maximum number of 30 patients, which was considered a feasible sample size that could be recruited at a small number of clinical sites over a reasonable time period.

**Fig 2:**
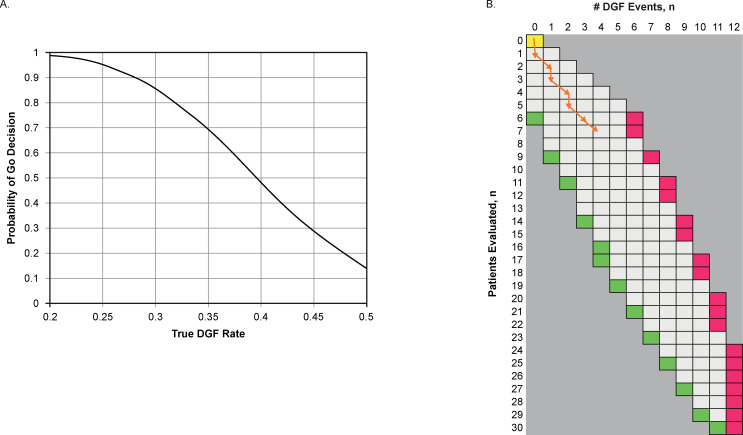
Sequential decision rules. (A) Probability of a Go decision by DGF rate* (B) Sequential Go/No Go/Continue rule^†^. *At a maximum of 30 patients, this design yields the probability of a Go decision of 0.139 when the GSK1070806 DGF rate is 50% (i.e., null hypothesis) and 0.69 at what has been a clinically impactful GSK1070806 DGF rate of 35%. ^†^The number in the first column indicates the number of patients who have completed study treatment. A sequential Go/No Go/Continue rule is based on the predictive probability of success. A high predictive probability (PP) of success means that GSK1070806 is likely to be efficacious by the end of the study given the observed data, whereas a low PP suggests that the treatment may not have sufficient activity. If the PP value <2% (red region) the alternative hypothesis is rejected. If the PP is >92% (green), the conclusion may be made that GSK1070806 has better efficacy than the standard of care. If the PP is 2–92% (white region), the trial will continue to the next interim or until reaching 30 completed patients. The sequential path observed in the study is represented by the orange line. Although the pathway ends in the white region, the decision to terminate the study was made as only 1 of the 7 patients completing study treatment was not on dialysis and had creatine <400 μmol/L; this suggested that it was unlikely that GSK1070806 3 mg/kg reduced the risk of DGF. DGF, delayed graft function.

Although a maximum cohort size of 30 was selected for this study, the actual number was determined based on patients’ sequential DGF outcomes. The sample size and decision criteria were selected to be adequately powered to detect the prespecified treatment effect and so there was a reasonable type 1 error rate in the event treatment was ineffective. The design yielded the probability of an erroneous “Go” decision of 0.139 when the GSK1070806 DGF rate is 50% (i.e., null hypothesis), and a probability of an appropriate “Go” decision of 0.659 if a DGF rate of 35% occurs **(Fig 2A; S5 Table in S1 File)**.

“Success” at full enrolment was defined as 11 or fewer DGF events in 30 patients (<37% DGF rate) with the outcome of the study depending on the DGF rate in the presence of GSK1070806 administration. With patient DGF status evaluated sequentially as each patient reached 7 days post transplantation, early success on the primary endpoint would be declared if the predictive probability of success was >0.92 (i.e., 92% probability the eventual number of DGF events would be 11 or fewer if the study continued to full enrolment) and a failure would be declared on the primary endpoint if the predictive probability of success was <0.02. **Fig 2B** illustrates which events may trigger an early Go (success) decision as the predictive probability of success is >0.92 (green box), and a No Go (failure) decision as the predictive probability of success is <0.02 (red box).

A summary of statistical analyses for the study endpoints can be found in **S6 Supplementary Material in S1 File**.

### Pharmacokinetic sampling

Blood samples were collected at 4–8 hours post reperfusion, Day 1, Day 2, Day 7 (or earlier at discharge), Day 30, Day 90, Month 6, and Month 12. GSK1070806 levels were measured with an immunoassay as previously described [29]. To calculate the area under the curve, the linear trapezoidal method was employed for all incremental trapezoids arising from increasing concentrations and the logarithmic trapezoidal method was used for decreasing concentrations (i.e., Linear Up/Log Down calculation method in Phoenix WinNonlin Professional [Certara, NJ, USA]).

### Pharmacodynamics and biomarkers

#### Serum

Blood samples were collected at baseline (pre-operative), 45 minutes, and 4–8 hours post reperfusion, and following transplantation on Day 1, Day 2, discharge, Day 30, Day 90, Month 6, and Month 12. Levels of free IL18 (unbound), IL18-GSK1070806 complexes, and total IL18 (includes free IL18, IL18 bound to GSK1070806, and/or IL18 binding protein [IL18-BP]) were measured in serum as previously described [29]. Antibody 16D10, used as the capture antibody in total and IL18/GSK1070806 complex assays, is specific for the mature form of IL18 and does not compete for IL18-BP. Antibody 13G9, used as the detection antibody in the free IL18 assay, is specific for both pro and mature forms of IL18, and competes for IL18-BP. Additionally, the free IL18 assay used GSK1070806 as the capture antibody, so the free IL18 assay was also specific for mature IL18. R&D systems Quantizing® Elisa Human IL18 BPa Kit was used for detection of serum IL18-BP. Recombinant human IL18 BPa reference standard was reconstituted and assays were conducted according to manufacturer specifications.

Levels of serum IP10 and MIG were measured using individual Meso-Scale Discovery immunoassays, Human IP10 V-plex plus (K151NVG) and Custom MIG K151A0H-2.

#### Urine

Urine samples were collected at baseline (pre-operative), 4–8 hours post reperfusion, and daily following transplantation until discharge, at discharge, Day 30, Day 90, Month 6, and Month 12. Aliquots of urine were collected in sterile, screw-capped polypropylene tubes, frozen immediately and stored at −20°C. Total urine IL18 was assessed as described for serum IL18. Kidney Injury Molecule-1 (KIM1) and neutrophil gelatinase-associated lipocalin (NGAL) levels were assessed using assays performed by Biomarker services, Q^2^ Solutions®.

#### Renal biopsies

Graft biopsies (core or wedge samples) were collected approximately 45 minutes post reperfusion and fixed in 10% neutral buffered formalin, then processed to paraffin block. Paraffin sections were cut at 4 microns, then stained using immunohistochemistry (IHC) Ventana Ultra Protocol 1249. GSK1070806 was detected using a mouse anti-GSK1070806 idiotype antibody 1H03. Total IL18 (pro and mature) was detected using a rabbit anti-IL18 antibody (Origene TA590088). An automated protocol was optimized using the Ventana Discovery Ultra staining system. Anti-idiotype antibody staining in a non-dosed, control kidney obtained from the National Disease Research Interchange was used to set the non-specific background threshold.

## Results

### Dose selection modeling

A PBPK modeling approach was used to estimate an appropriate IV dose of GSK1070806 [32, 33] in renal transplant recipients. Briefly, this technique uses physiological volumes (vascular, extracellular, and endosomal) and fluid flows (lymph/plasma) combined with mechanistic calculations of extravasation, binding, and neonatal Fc Receptor recycling to predict drug distribution and elimination. As part of the validation, verification was conducted in which, model-simulated plasma PK parameters were compared with measured PK from healthy volunteers, showing good agreement (**S4 Supplementary Material, and S3 Table in S1 File**).

The kidney interstitium was considered the critical site of action, as IL18 signaling on the basolateral receptors of the renal tubular epithelial cells is thought to mediate apoptotic signaling [34]. To simulate a worst-case scenario for achieving target suppression, all IL18 synthesis was assigned to the kidney interstitium as a zero-order reaction and was carried to plasma by lymphatics, and to a lesser extent diffusion into capillaries. The IL18 generation rate was adjusted to produce an IL18 plasma concentration of 835 pg/mL, which is consistent with the 90^th^ percentile of patients with DGF [35]. The degradation rate was set to achieve an IL18 half-life of 35 hours [36]. GSK1070806 doses of 0.1–10 mg/kg were modeled (**S1 Fig in S1 File**) with the goal of identifying dose levels to achieve a >90% reduction versus baseline in free IL18 levels in the kidney interstitium within 1–2 hours of kidney reperfusion post transplantation.

**Fig 3:**
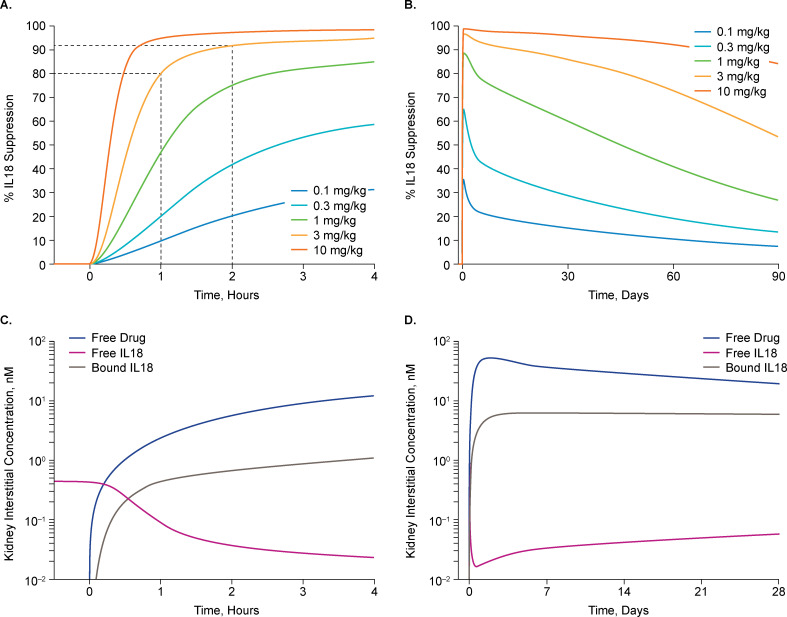
Simulation of drug exposure in the kidney interstitium based on IL18 suppression using PBPK modeling up to 6 hours (A) and 90 days (B) post dose. Predicted drug concentration and free/bound IL18 in the kidney interstitium up to 4 hours (C) and 1-month (D) post dose with 3 mg/kg. IL18, interleukin-18, PBPK, physiologically-based pharmacokinetic modeling.

Simulations of the 3 mg/kg dose indicated a rapid and robust decrease in free IL18 levels in the kidney interstitial space with >80% reduction by 1 hour and >90% achieved 2 hours post dose (**Fig 3A and 3B**). Maximum knockdown in free IL18 levels in the kidney of 96% was expected approximately 43 hours post dose, inhibition >90% was maintained for 7 days (period of assessing the DGF primary endpoint), and >85% target engagement was sustained through the simulated 28-day time course (**Fig 3C and 3D**). Use of the single 3 mg/kg dose provided a >99% reduction in circulating (plasma) levels of free IL18 within minutes post dose and sustained over the following 28 days (**S1 Fig in S1 File**). The kidney interstitium is considered a difficult tissue to penetrate, so the extensive penetration modeled for the kidney suggests that GSK1070806 exposure should also be sufficient in other tissues if they are relevant for IL18 DGF pathophysiology.

Numerous sensitivity analyses (**S2 Fig in S1 File**) were performed around renal blood and lymph flow (1–300%), IL18 half-life (1–100 hours), circulating IL18 levels (100–3000 pg/mL), and capillary-tubule distance (up to 600 μm), all supporting that the single 3 mg/kg IV dose was the minimum that would confidently achieve >90% IL18 inhibition in the initial hours after transplantation, during which it was presumed that the pathological processes causing DGF would be initiated.

Further details of the simulation methodologies, model validation, and sensitivity analysis are included in the **S4 Supplementary Material, and S1–S3 Figs and S1–S3 Tables in S1 File**.

### Patient and donor characteristics, and trial flow

Ten patients were screened, and seven patients were enrolled and received a single GSK1070806 3 mg/kg IV dose. One patient was lost to follow-up prior to the 12-month study visit (**Fig 1B**). Donor- and recipient-paired baseline demographic characteristics are summarized in **Table 1**; additional baseline characteristics of the recipients are shown in **Table 2**. All patients were male between 33 and 72 years of age. The study was terminated primarily due to an overall lack of efficacy after the seventh patient was assessed for DGF. Recruitment was also slower than expected.

**Table 1:** Donor and recipient paired baseline demographic characteristics and DGF outcomes.

Donor				Recipient					
Age (years)	Sex	WIT (min)	CIT (min)	Patient #	Age (years)	Sex	Weight (kg)	Sequence*	DGF
61	M	9^†^	811	1	71	M	80	1	**N**
57	F	22	978	2	63	M	107	2	**Y**
65	M	9	678	3	56	M	111	3	**N**
36	F	19	793	4	33	M	87	6	**Y**
59	M	15	514	5	64	M	98	4	**Y**
52	M	15	540	6	72	M	64	5	**N**
61	M	ND	1246	7	52	M	104	7	**Y**

All donor and recipients were of European ethnicity. None of the donor kidneys were transported using a cold machine perfusion system, except that received by Patient 54. Only DCD kidneys were used.

*Sequence indicates the order of evaluation for Bayesian sequential study design (**Fig 2B**).

^†^Donor WIT for Patient 1 was originally erroneously reported as 4 minutes as it did not include the 5-minute mandatory waiting period between circulatory arrest and initiation of surgery, per UK legislation.

DCD, donation after circulatory death; DGF, delayed graft function; CIT, cold ischemic time; N, no; ND, no data; WIT, warm ischemic time; Y, yes.

**Table 2:** Baseline demographics and clinical characteristics of patients receiving GSK1070806.

	GSK1070806 3 mg/kg IV (N = 7)
**Age (years), mean (SD)**	58.7 (13.46)
**Sex, n (%)**	
Female	0 (0)
Male	7 (100)
**Ethnicity, n (%)**	
Hispanic or Latino	0 (0)
Non-Hispanic or Latino	7 (100)
**Height (cm), mean (SD)**	179.1 (9.35)
**Weight (kg), mean (SD)**	92.97 (16.806)
**BMI (kg/m**^**2**^**), mean (SD)**	29.20 (6.014)
Current medical conditions*	
Cardiovascular risk factors, n (%)	
Any condition	7 (100)
Hypertension	7 (100)
Hyperlipidemia	2 (29)
Angina pectoris	1 (14)
Diabetes	1 (14)
Renal and urinary disorders, n (%)	
Any condition	5 (71)
Glomerulonephritis/autoimmune	2 (29)
Hypertensive nephropathy	1 (14)
Polycystic kidney disease	1 (14)
Sequelae of systemic autoimmunity	1 (14)
Other condition, n (%)	
Any condition	6 (86)
Respiratory, thoracic, and mediastinal disorders	2 (29)
Gout	2 (29)
Anemia normocytic	1 (14)
Endocrine disorders	1 (14)
Hepatobiliary disorders	1 (14)
Immune system disorders	1 (14)
Known left ventricular hypertrophy	1 (14)
Musculoskeletal and connective tissue disorders	1 (14)
Psychiatric disorders	1 (14)
Pacemaker inserted	1 (14)
Scleritis	1 (14)
Vascular disorders	1 (14)
Anemia	1 (14)

*Current medical conditions were collected as per the protocol but summarized post-hoc; patients may have had more than one current medical condition.

### Efficacy

Of the seven patients enrolled, four exhibited DGF (**Table 1**). This outcome (57% DGF rate, 95% credible interval [CrI]: 25–90%) exceeded the 50% expected standard-of-care rate. The sequential path in **Table 1,** represented by the orange line in the grid in **Fig 2B,** was as follows (note that patients were not evaluated in the same order as they were enrolled): no DGF (Patient 1), DGF (Patient 2), no DGF (Patient 3), DGF (Patient 5), no DGF (Patient 6), DGF (Patient 4), and DGF (Patient 7), with Patient 6 receiving dialysis for hyperkalemia. Only one of the seven patients (Patient 1) had creatinine levels <400 μmol/L and did not require dialysis over the first 4 days. The posterior probability that <30% of patients would experience DGF was 7% (95% CrI: 0–100%), and the posterior probability that <50% of patients would experience DGF was 34% (95% CrI: 0–100%).

**Fig 4:**
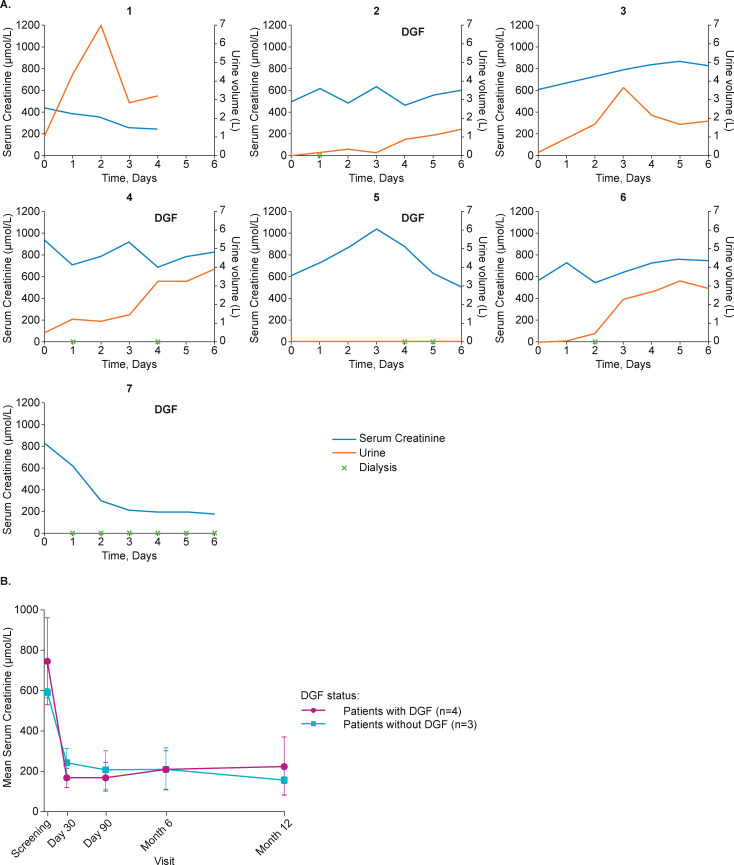
(A) Creatinine levels and urine output during the first 7 days following transplantation. (B) Mean serum creatinine by DGF occurrence over the study course. There was some variability between patients in timeframe and extent of data collection: Patient 1, no data collected for Days 5 and 6; Patient 5, urine output was negligible during this period; Patient 7, urine output not recorded due to patient undergoing continued dialysis during this period. Any event occurring at any time within a calendar day was charted at that study day, e.g., for Patient 4, dialysis at Day 1 as shown on the chart may have occurred at any time point during Day 1. DGF, delayed graft function.

Serum creatinine was elevated at baseline in all patients, ranging between 448 μmol/L and 996 μmol/L, and generally remained elevated over 6 days post transplantation (**Fig 4A**). Overall, serum creatinine had decreased at 12 months in all patients, irrespective of the presence of DGF (**Fig 4B and S4 Fig in S1 File**). A protracted recovery of urine output was observed in most cases (**Fig 4A**).

### Safety and tolerability

Six (86%) patients experienced a total of 17 serious adverse events (SAEs) (**S6 Table in S1 File**). The seventh patient enrolled developed an SAE of ventricular fibrillation and cardiac arrest (non-fatal) intra-operatively. This event was considered unrelated to GSK1070806, as the patient had multiple risk factors for coronary artery disease and had been experiencing chest pain prior to transplantation. Subsequently, this patient also developed an SAE of visceral leishmaniasis not considered related to study treatment. Another patient experienced two SAEs, pneumonia and respiratory arrest, considered related to study treatment. In a further patient, an SAE of acute cellular rejection was observed at Day 57 and was considered unrelated to treatment, with a biopsy at Day 59 showing a borderline rejection result. All SAEs were resolved and no fatal SAEs occurred.

All patients experienced ≥1 AE, most frequently anemia (5/7; 71%), constipation (4/7; 57%), diarrhea (3/7; 43%), and urinary tract infection (3/7; 43%). No AEs led to study withdrawal. Five patients experienced AEs considered related to GSK1070806 (**Table 3**), most commonly anemia (4/7; 57%), as well as several infections, including urinary tract infections, bacterial disease carrier, nail infection, pharyngotonsillitis, postoperative wound infection, purulent discharge, acute pyelonephritis, influenza, and lower respiratory tract infection. With the exception of the SAE of cardiac arrest, there were no clinically significant electrocardiography abnormalities during the study. While other clinically significant laboratory abnormalities were recorded during the study, they were as expected for this patient population.

**Table 3:** Summary of drug-related adverse events by system organ class.

System organ class preferred term	Patients, n (%) N = 7
**Any event**	5 (71)
**Blood and lymphatic system disorders**	
Any event	5 (71)
Anemia	4 (57)
Leukopenia	1 (14)
**Investigations**	
Any event	3 (43)
Blood temperature decreased	1 (14)
Cytomegalovirus test positive	1 (14)
Hemoglobin increased	1 (14)
**Infections and infestations**	
Any event	2 (29)
Influenza	1 (14)
Lower respiratory tract infection	1 (14)
Pneumonia	1 (14)
**Injury, poisoning and procedural complications**	
Any event	2 (29)
Arteriovenous fistula site complication	1 (14)
Wound dehiscence	1 (14)
**Skin and subcutaneous tissue disorders**	
Any event	2 (29)
Pruritis	1 (14)
Urticaria	1 (14)
**Gastrointestinal disorders**	
Any event	1 (14)
Lip swelling	1 (14)
**Metabolism and nutrition disorders**	
Any event	1 (14)
Hypoalbuminemia	1 (14)
Hypomagnesemia	1 (14)
**Respiratory, thoracic and mediastinal disorders**	
Any event	1 (14)
Respiratory arrest	1 (14)

### Pharmacokinetics

Plasma PK results for GSK1070806 are summarized in **S7 Table in S1 File**. A mean (95% confidence interval [CI]) maximum observed concentration of 53.14 (29.69–76.59) μg/mL was achieved. Mean (95% CI) serum half-life was 37.96 (32.77–43.15) days and mean (95% CI) area under the concentration-time curve from time zero to infinity was 43.27 (27.34–59.19) h*mg/mL.

### Pharmacodynamics and biomarkers

**Fig 5:**
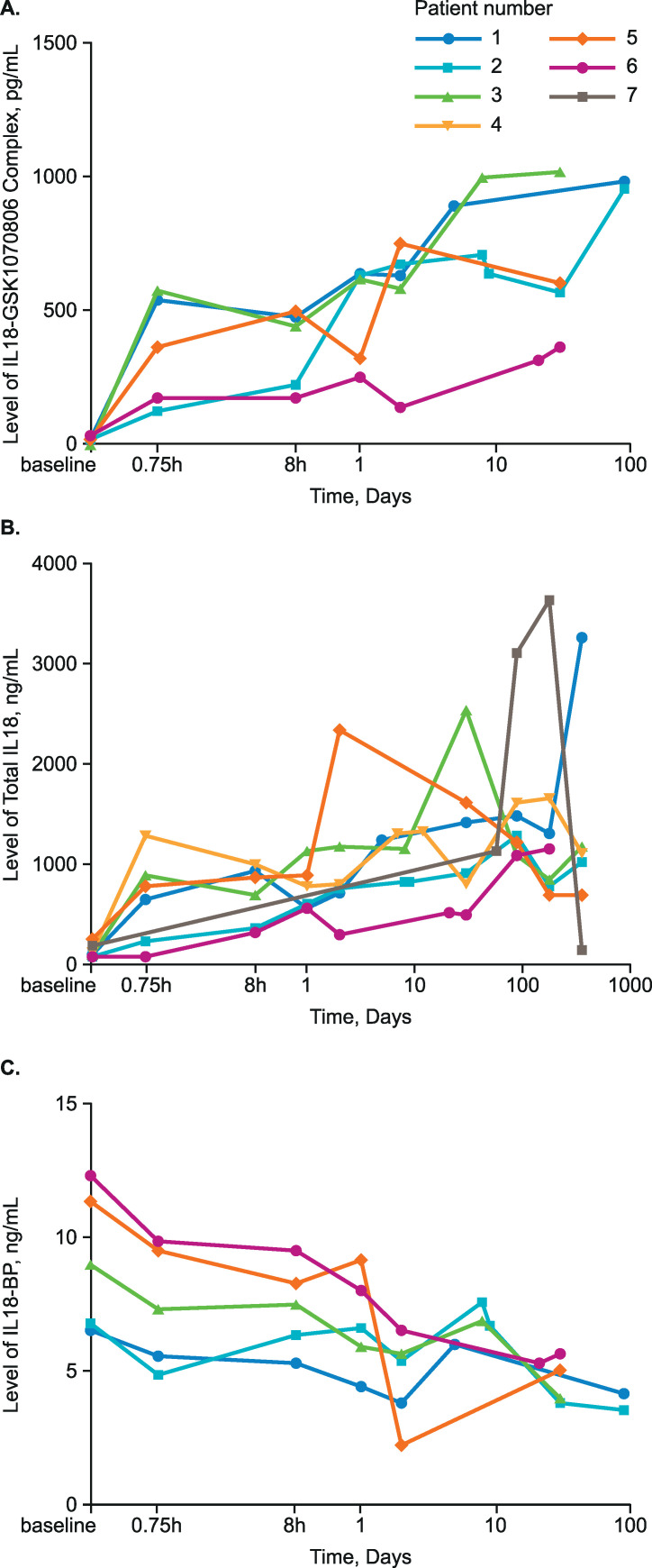
Serum levels of (A) IL18-GSK1070806 complex, (B) total IL18, and (C) IL18-BP over the study course. BP, binding protein; IL18, interleukin-18. Figures produced post hoc.

Serum IL18 levels (total and IL18-GSK1070806 complex) rose rapidly following dosing and transplantation and remained high for a prolonged period (**Fig 5A and 5B**). Serum IL18-BP levels were reduced by as much as 2-fold following treatment (**Fig 5C**). Levels of free IL18 were predominantly at or below the lower limit of quantification at baseline and throughout the study; as a result, sampling was discontinued part-way through the trial, therefore data are sparse.

Urinary IL18 levels were monitored in five patients; levels were low, except in two patients (Patients 2 and 3) who had levels above 100 pg/mL within the first 2 postoperative days, which is considered predictive of increased AKI risk [37] (**S8 Table in S1 File**). After transplant, urinary NGAL levels were elevated above 153 ng/mL and urinary KIM1 levels were above 2.37 ng/mL in all six patients for whom data were available (Patients 1–6); these levels of NGAL [38] and KIM1 [37] are considered predictive of AKI progression (**S8 Table in S1 File**).

**Fig 6:**
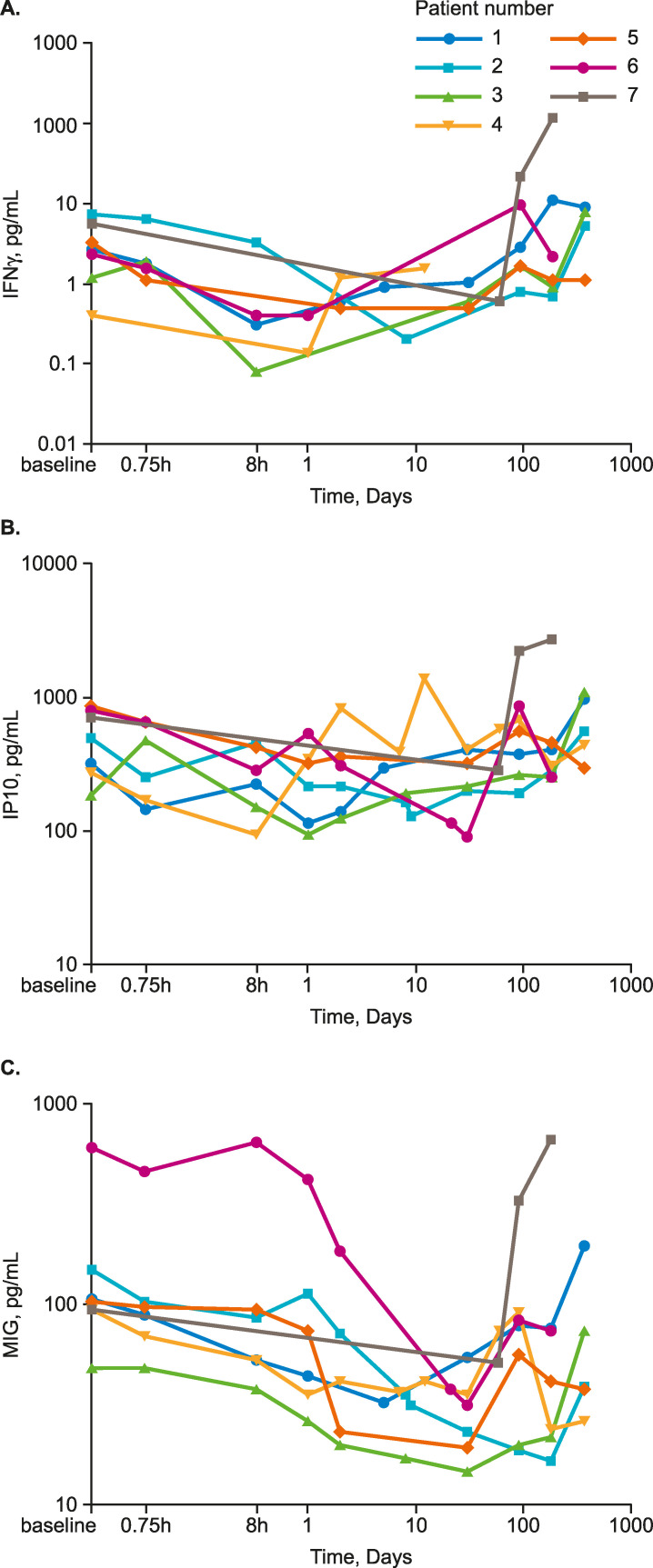
Serum cytokine levels over the study course. (A) IFNγ, (B) IP10, (C) MIG. IFNγ, interferon-γ; IP10, IFNγ-induced protein 10 Kd; MIG, monokine induced by IFNγ. Figures produced post-hoc.

Serum levels of IFNγ, which is directly induced by IL18, were low (between 0.05 pg/mL and 11 pg/mL) except in Patient 7, in whom they rose to 22 pg/mL by 90 days and 124 pg/mL by 6 months. Generally, IFNγ decreased to Day 1, after which levels increased. IP10 levels were largely unchanged over the course of treatment, except in Patient 7, in whom the levels decreased through Day 58 and increased nearly 4-fold from pretreatment levels thereafter. In Patient 4, after an initial decline to 8 hours post dose, IP10 levels increased and generally remained elevated, with two transient peaks at Day 2 and Day 12; this patient experienced transplant rejection on Day 57. Serum MIG levels generally decreased across the first 30 days, before beginning to increase towards the end of the study period. Changes in MIG levels mirrored those of IFNγ and IP10 in Patient 7 (**Fig 6**).

Levels of additional serum cytokines IL1β, IL2, IL8, macrophage inflammatory protein 1β, monocyte chemoattractant protein, and tumor necrosis factor-α are shown in **S5 Fig in S1 File**. Overall levels were consistent with low or diminishing proinflammatory responses over the course of treatment.

### Histology

Histological assessments of biopsies taken approximately 45 minutes post reperfusion showed varying degrees of pre-existing pathology that may have contributed to the attenuated allograft function observed in this study. Hematoxylin and eosin staining revealed moderate tubular vacuolation and basement membrane thickening in DGF Patients 2 and 4, and extensive basement membrane thickening, increased connective tissue, and dilated Bowman’s space in DGF Patient 5 (**S6 Fig in S1 File**). No biopsies were available for analysis for DGF Patient 7 or no-DGF Patient 6.

**Fig 7:**
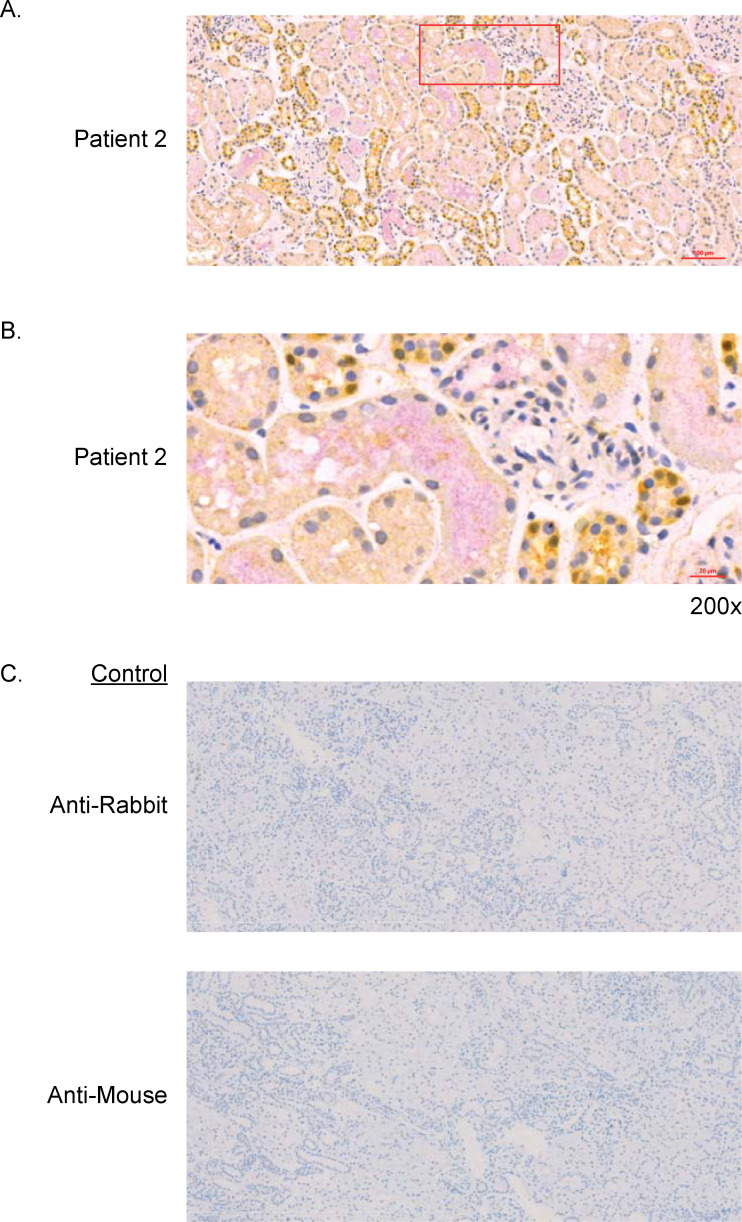
Dual immunohistochemical detection. (A) Co-distribution of GSK1070806 (pink) with IL18 (yellow) in wedge biopsies from Patient 2. Nuclei stained with hematoxylin. (B) 200x magnified region of Patient 2 biopsy, taken from the area outlined in A, showing the luminal co-distribution of GSK1070806 (pink) and IL18 (yellow) in the tubules. (C) Isotype control staining. IL18, interleukin-18.

Total tissue IL18 (pro-IL18 and mature IL18) and GSK1070806 staining was assessed by IHC in five of the seven patients (**Fig 7A**). GSK1070806 staining was observed within the epithelium of a subset of distal tubules, was distributed heterogeneously, and coincided to a limited degree with total IL18 expression in the same regions (**Fig 7B**). Signal-to-noise within and between patients was variable although background IgG1 isotype staining did not contribute to this (**Fig 7C**). A non-dosed and non-transplanted control kidney was stained with GSK1070806 as the primary antibody (to identify mature IL18 only), showing strong IL18 staining in a subset of tubular epithelial cells and moderate staining in the luminal spaces of a subset of tubules, consistent with the pattern of anti-IL18 staining in biopsies from the study (**S7 Fig in S1 File**).

## Discussion

In this study, neutralization of IL18 with GSK1070806 was evaluated for the prevention of DGF following renal transplantation using a single-arm Bayesian sequential design. Four of seven patients (57%) satisfied the definition of DGF. Two further patients had high creatinine levels in the postoperative period, one of whom required dialysis for hyperkalemia, leaving only one of seven patients who was not on dialysis and with creatine <400 μmol/L. Collectively, these data suggested that it was unlikely that GSK1070806 3 mg/kg reduced the risk of DGF. Based on this evidence, the trial was terminated.

Several clinical studies have been performed to evaluate the effect of novel investigational medicines on the incidence of DGF. These studies have been largely unsuccessful and require the recruitment of large numbers of patients using placebo comparator designs to evaluate efficacy [39]. Utilizing a Bayesian approach to minimize sample size reduces investment barriers to evaluating new therapies (both clinical trial resource and funding), exposes fewer patients to investigational medicines with limited safety profiles, and enables efficient decision making. Indeed, the novel Bayesian sequential approach in this study, using the background DGF rate determined from literature and confirmed with registry data formed the Go/No Go design, allowed for fewer patient exposures and avoided the need for a control arm. Each patient enrolled in this study had a varied risk for DGF at baseline based on their individual characteristics. This was accounted for as closely as possible when confirming the background DGF rate using registry data, in which only patients meeting the current clinical trial inclusion/exclusion criteria and treated at recruiting sites were used to confirm the 50% DGF background rate. From a development perspective, if this initial proof-of-concept study had indicated that there was the potential for GSK1070806 to reduce DGF, the results would have been confirmed in a randomized double-blind placebo-controlled study.

Consistent with the poor renal function in 6/7 patients in the study, urinary levels of NGAL and KIM1 were elevated post-transplant in all six patients for whom data were available. Urinary IL18 levels were low, except in two patients, whose elevated levels were observed immediately following or within 2 days post-transplantation. One possible explanation for the overall lower levels of urinary IL18 could be sequestration of IL18 within the kidney or circulation via formation of IL18-GSK1070806 complexes. Furthermore, urinary IL18 levels appear non-correlated with postoperative graft function, as the patient with the highest urinary IL18 level did not experience DGF.

PD analyses showed serum levels of total IL18 and IL18-GSK1070806 complexes climbed rapidly following transplantation and remained high for a prolonged period, reflecting peripheral blood IL18 target engagement with GSK1070806 enhancing IL18 half-life. This is consistent with the first-in human study [29]. However, serum levels of total IL18 in this study showed large variation between patients, especially by the end of the study. This is perhaps not surprising, given that the first-in-human study included relatively healthy participants, whereas the present study comprised a complex patient population. Extraneous factors may have been involved in the observed IL18 variability, such as visceral leishmaniosis infection in Patient 7 and pyelonephritis and rejection events in Patient 4.

Although acute allograft rejection was not assessed directly in this study (e.g., via surveillance biopsies), an exploratory evaluation of the potential impact of GSK1070806 on acute allograft rejection was performed by measuring serum levels of IFNγ and IFNγ-inducible chemokines, IP10 and MIG, which are predictive of acute rejection episodes [22–24]. Levels of MIG in most patients were unchanged over the study and levels of IFNγ and IP10 initially declined in most patients following treatment, consistent with antagonism of IFNγ−induced Th1 cytokine responses and predicting a low probability of acute rejection occurrence. One case of acute rejection occurred (Patient 4; event considered unrelated to study treatment) with elevated levels of IP10, MIG and total IL18 corresponding to the rejection event. No rejection was observed in Patient 7 despite steady increases in IP-10 and MIG throughout the study, suggesting their elevated levels were associated with a different mechanism (e.g., long-term sequelae of visceral leishmaniasis).

While these data indicate engagement in the circulation, they may not reflect GSK1070806 activity in the kidney interstitium, which could be the relevant site of action. By IHC of kidney biopsies, we were able to identify the presence of GSK1070806 outside of the renal vasculature, indicating that GSK1070806 did penetrate the transplanted kidney tissue. GSK1070806 selectively binds human mature IL18 with a high affinity (Kd = 46.0 pM at 37°C) and neutralizes its function [30], but GSK1070806 has a much lower affinity for pro-IL18 (Kd = 46.7 nM at 37°C) (**S7 Supplementary Material, S4 Table in S1 File**). Therefore, we would not expect GSK1070806 to bind pro-IL18 at the anticipated GSK1070806 exposures in vivo. The IL18 antibody used for IHC labeling in renal biopsies from patients treated with GSK1070806 in this study does not differentiate between pro and mature IL18, so the observed staining results potentially include both forms. With just one post-dose biopsy from each patient, we are unable to eliminate the possibility of regional differences in drug distribution. Additional confounding factors include the small sample size, inter- and intra-sample variation, and the type of biopsy collected (3 wedge and 2 needle). Therefore, the IHC findings should be interpreted with caution: solely based on the IHC data we cannot conclude whether therapeutically relevant exposure was achieved within the kidney during acute reperfusion (as predicted by the PBPK modeling); we can conclude only that GSK1070806 was present along with pro and/or mature IL18.

Achieving exposure within the interstitium of the kidney with sufficient rapidity to inhibit IL18 after kidney reperfusion was deemed of potentially critical importance in this study and triggered substantial modeling efforts prior to dose selection. Extensive sensitivity analyses completed using the PBPK modeling approach allowed us to confidently select the single GSK1070806 3 mg/kg IV dose level evaluated here for its ability to reduce the occurrence of DGF. Dose escalation to 10 mg/kg was considered following the lack of efficacy observed in this study. However, the PBPK modeling results predicted substantial IL18 inhibition in the first hours following transplantation at the 3 mg/kg dose, and it was considered unlikely that increasing exposure with the maximum dose previously tested (10 mg/kg) would improve efficacy (Fig 3). Although the renal interstitium was hypothesized as the site of action for GSK1070806, if neutralization was only required in the circulation GSK1070806 at the 3 mg/kg dose was predicted to achieve >99% IL18 suppression within minutes of administration. Note, plasma PK in this study was in line with that observed in the first-in-human study following administration of a single 3 mg/kg dose [29], and a phase II study assessing GSK1070806 in patients with type 2 diabetes [30]. However, we cannot preclude the possibility that IL18 neutralization occurred too late in the pathological process of DGF. Although we hypothesized that injury occurs upon reperfusion, it is possible that it occurs prior to transplantation, which could be an explanation for the lack of efficacy observed in this study. While it is possible that IL18 inhibition in donors prior to kidney removal or during organ transport (e.g., through a normothermic perfusion/preservation circuit) would have been beneficial in reducing risk of DGF, ethical and operational issues make these hypotheses difficult to evaluate and no such studies are planned.

All patients reported AEs, and 6 of the 7 patients enrolled experienced SAEs; all SAEs were resolved and there were no fatalities. Overall, the AEs recorded were as expected for patients undergoing kidney transplantation; indeed, all patients had multiple comorbidities and suboptimal kidney function. Although most patients reported SAEs consistent with an unwell transplant population, only two of the SAEs, both occurring in one patient, were considered related to GSK1070806 (pneumonia and respiratory arrest). Relatedness was indicated due to the potential of the study drug to magnify immunosuppression. In addition to the drug-related SAE of pneumonia, other non-serious infections considered related to GSK1070806 occurred. One patient experienced a cardiac event, which was deemed unrelated to study treatment. This patient was found to have critical coronary stenoses on post-operative angiography, which suggested a chronic cause of cardiac complications. End-stage renal disease is associated with two-fold increase in risk of death from cardiovascular disease [40] and, in the post renal transplant setting, cardiovascular disease is reported to be the leading cause of death with graft function (36.1%) [40]. In previous studies, GSK1070806 was well tolerated with no reported cardiac complications [29, 30]; however, we cannot completely rule out a possible effect of the study drug in the observed cardiac event.

In conclusion, this study does not provide data supporting efficacy of GSK1070806 in preventing DGF after DCD renal transplantation in the recruited this patient population. However, utilization of the novel Bayesian sequential study design enabled rapid decision making to not proceed further with the development of GSK1070806 for renal transplantation, and hence avoided excess exposure of patients to the investigational drug. Although we were not able to measure target engagement directly in the kidney interstitium, based on PBPK modeling and associated sensitivity analyses, we are compelled to think that the 3 mg/kg dose provided sufficient exposure in these patients; and that our negative clinical findings are due to a lack of efficacy in response to IL18 neutralization at the time of reperfusion, rather than poor exposure/target engagement with GSK1070806.

## Supporting information

S1 Checklist TREND statement checklist. (PDF)

S1 File (DOCX)

S2 File (PDF)

S1 Fig (EPS)

S2 Fig (EPS)

S3 Fig (EPS)

S4 Fig (EPS)

S5 Fig (EPS)

S6 Fig (EPS)

S7 Fig (EPS)
