# Supplementary material for: A pilot study evaluating GSK1070806 inhibition of interleukin-18 in renal transplant delayed graft function
Source: PLoS One. 2021 Mar 8;16(3):e0247972. doi: 10.1371/journal.pone.0247972 (PMC7939287; doi:10.1371/journal.pone.0247972)
Supplement: S1 File — (DOCX) [file pone.0247972.s002.docx]

# S1 Supplementary Material. Withdrawal/stopping criteria

A patient could have withdrawn from study participation at any time at his/her own request, or at the discretion of the investigator for safety, behavioral or administrative reasons. As patients received a single dose of study medication on Day 0, withdrawal was defined as a patient declining to participate in follow-up activities after the dose was administered.

Patients who withdrew were encouraged to return for subsequent visits, particularly the Safety Follow-up visit at 12 months, and to continue with adjustments to infectious prophylaxis/standard of care medicines intended to mitigate safety risks associated with administration of a long-acting, novel immunosuppressive.

# S2 Supplementary Material. Prophylaxis and surveillance for infection

As GSK1070806 is a cytokine antagonist, and was administered in addition to standard-of-care immunosuppressive therapy, intensive prophylaxis and surveillance against perioperative bacterial, pneumocystis, viral and fungal infections was considered appropriate.

## Pneumocystis and fungal infection prophylaxis

A minimum of 3 months of pneumocystis and fungal infection prophylaxis with cotrimoxazole and nystatin, respectively (or equivalents), was implemented unless otherwise contraindicated. In cases of cotrimoxazole intolerance, alternative therapy was considered at the discretion of the investigator.

## Cytomegalovirus (CMV) infection

Patients at risk of CMV were defined as:

- Donor positive/Recipient negative (risk of primary infection)
- Donor positive/Recipient positive (risk of re-infection)
- Donor negative/Recipient positive (risk of re-activation)

For these patients, CMV infection prophylaxis with a 200-day course of valganciclovir was administered. If the patient received additional immunosuppression for allograft rejection during the course of prophylaxis, the course was extended and the patient received treatment for 200 days afterwards. If anti-rejection medicines were administered 6–12 months post-transplantation, a repeat 200-day course of valganciclovir was prescribed.

Patients that were Donor positive and Recipient negative for CMV received prophylaxis with acyclovir for 200 days.

## BK virus

In the event that a patient became BK positive (assessed by BK polymerase chain reaction of whole blood), it was possible for immunosuppression to be reduced or titrated at the discretion of the investigator. In the event of deteriorating renal function in the context of BK viremia, a transplant biopsy to diagnose BK nephropathy was considered at the discretion of the investigator.

# S3 Supplementary Material. Exclusion criteria

A patient was not eligible for inclusion in the study if any of the following criteria applied:

1. Concurrent conditions/medical history
   1. Liver function: alanine aminotransferase >2x the upper limit of normal (ULN) and bilirubin >1.5xULN (isolated bilirubin >1.5xULN is acceptable if bilirubin is fractionated and direct bilirubin <35%).
   2. QT interval: single or average QTc >480 msec or in patients with bundle branch block QTc >500 msec (these criteria do not apply to patients with predominately paced rhythms).
   3. Ventricular arrhythmia: history of cardiac arrest or malignant ventricular arrhythmia at any time. Any acute cardiovascular syndrome within 6 months before screening. These may include acute coronary syndrome (myocardial infarction or unstable angina) or coronary intervention (percutaneous or surgical), or acute stroke.
2. Concomitant medications
   1. Concurrent medication: patients who receive treatment that is prohibited for safety reasons (eg, live vaccines, cyclophosphamide, or other biologic immunosuppressants) should not receive investigational product without the explicit approval of the Medical Monitor (Sponsor).
   2. Investigational product: any within 5 half-lives or twice the duration of the biological effect whichever is longer (investigational product refers to any drug not approved for sale in the country in which it is being used).
   3. Immunosuppression: are being considered for steroid-free, anti-thymocyte globulin or alemtuzumab induction, which have a much more profound and prolonged immunosuppressive effect than basiliximab.
   4. Prior biologic immunosuppressives: has received an agent within the following time period prior to the day of dosing in the current study: 30 days, 5 half-lives or twice the duration of the biological effect, whichever is longer.
   5. Vaccines: a live vaccine within 30 days prior to GSK1070806 administration.
3. Contraindications
   1. Receiving a Donation after Circulatory Death kidney allograft from a donor with any of the following characteristics:
      1. Cold ischemic time >36 hours
      2. Age <5 years old
      3. ABO blood type incompatible against the recipient
      4. T- and/or B-cell positive crossmatch by complement dependent cytotoxicity or flow cytometry against the recipient (where positive crossmatch is unavailable, virtual crossmatch is allowed).
      5. Serology positive for hepatitis B (except hepatitis B surface antibody and prior vaccination), hepatitis C or human immunodeficiency virus (HIV)
      6. Epstein–Barr virus-positive donor allograft with an Epstein–Barr virus-negative recipient
      7. Donor had acute or chronic bacterial, viral or fungal infection that according to the investigator causes a risk to recipient, particularly if the infection was resistant or systemic
      8. Normothermic regional machine perfusion organ retrieval techniques were utilized
      9. Surgical damage to donor allograft during organ procurement (surgical damage is defined as damage requiring surgical repair to make the organ usable)
      10. “Uncontrolled” Maastricht Category 1 (dead on arrival), Category 2 (unsuccessful resuscitation), and Category 5 (unexpected cardiac arrest in a critically ill patient)
   2. Previous organ transplantation: has previously undergone any other organ transplantation (with the exception of first kidney and/or corneal transplantation).
   3. Malignancy: has a history of malignancy in the past 5 years except for adequately treated cancers of the skin (basal or squamous cell) or carcinoma in situ of the uterine cervix.
   4. Acute or chronic infection: has required management of acute or chronic infections (excludes prophylaxis of infections), as follows:
      1. Currently being treated for a chronic infection, which in the opinion of the investigator, could put the patient at undue risk
      2. Hospitalized for treatment of infection, or treated for an infection with parenteral antibiotics (includes antibacterials, antivirals, antifungals, or antiparasitic agents) within 30 days before Day 0, which in the opinion of the investigator, could put the patient at undue risk
      3. Current evidence, or history within the last 14 days, of an influenza-like illness as defined by fever (>38°C) and two or more of the following symptoms: cough, sore throat, runny nose, sneezing, limb/joint pain, headache, vomiting/diarrhea
      4. Patients with any history of active tuberculosis, recent tuberculosis exposure, or judged by investigators to be at risk of tuberculosis will be excluded from the study
   5. Other disease/conditions; has any of the following:
      1. Clinical evidence of significant unstable or uncontrolled acute or chronic diseases, which in the opinion of the investigator, could confound the results of the study or put the patient at undue risk
      2. A surgical procedure planned in the 12 months after Day 0, other than kidney transplantation or related procedure
      3. A known history of any other medical disease (eg, cardiopulmonary), laboratory abnormality, or condition (eg, poor venous access) that, in the opinion of the investigator, makes the patient unsuitable for the study
   6. Hepatitis B: patients will be excluded with any evidence of acute or chronic infection, or if interpretation of their results is unclear, including:
      1. Hepatitis B surface antigen+
      2. Anti-hepatitis B core antigen+
      3. Hepatitis B DNA+
      4. It is permissible to enroll patients who are hepatitis B surface antibody+ only, when this is attributable to vaccination and there is no history of previous infection.
   7. Hepatitis C: patients will be excluded if there is any evidence of past or current hepatitis C infection, including hepatitis C antibody, hepatitis C Recombinant ImmunoBlot Assay, or polymerase chain reaction.
   8. HIV: known to have a historically positive HIV test.
   9. Immunodeficiency: recipient with a history of, or laboratory evidence of immunodeficiency.
   10. Drug sensitivity: has a history of sensitivity to any of the study medications including:
       1. GSK1070806
       2. Background immunosuppressive regimen
       3. Designated prophylactic anti-infective therapies or components thereof, or a history of drug or other allergy including a previous anaphylactic reaction to parenteral administration or biologic therapy (ie, monoclonal antibody [mAb]) that, in the opinion of the Investigator or Medical Monitor, contraindicates their participation
   11. Substance abuse: has clinical evidence of current drug or alcohol abuse or dependence.
   12. Co-enrollment: participating in another interventional study (participation in purely observational or cohort studies is acceptable provided they do not impair feasibility or involve excessive additional sampling).
   13. Compliance: is unlikely to comply with scheduled study visits based on investigator judgment or has a history of a psychiatric disorder or condition that may compromise communication with the investigator.

# S4 Supplementary Material. Dose selection using a physiologically-based pharmacokinetics approach

A physiologically-based pharmacokinetic (PBPK) approach was used to estimate an appropriate dose. The site of action considered was the kidney interstitium, where interleukin-18 (IL18) signaling on the basolateral receptors of the renal tubular epithelial cells is thought to mediate apoptotic signaling. The dose was selected to achieve a reduction in free IL18 levels of >90% baseline within 1 hour of kidney reperfusion. To avoid confounding of expected pharmacodynamic (PD) effects with carrier effects of target suppression due to the prolonged half-life of IL18 when bound to GSK107806, the ratio of current free IL18 post-dose levels to free IL18 pre-dose levels is used rather than the more common receptor occupancy, generally described as the ratio of free IL18 to total IL18.

The PBPK approach used has been previously described in detail [1, 2]. Briefly, this modeling technique uses physiological volumes (vascular, extracellular, and endosomal) and fluid flows (lymph/plasma) combined with mechanistic calculations of extravasation, binding, and neonatal Fc Receptor recycling to predict drug distribution and elimination. In this simulation, free IL18, free GSK1070806, IL18-GSK107806 complex, and endogenous immunoglobulin G are allowed to co-circulate in the model. The binding equilibrium is described by a simple first order kinetic reaction in all compartments. Tissue physiologies, including total, interstitial, and plasma volumes as well as plasma flows, were found in the BioDMET database [3]. Endosomal volumes were set to 0.5% of the tissue volume [4], and all lymph flows except the kidney lymph flow were set to 1/500 the plasma flow to the tissue [5]. The kidney lymph flow was set to 1.75 mL, consistent with the mean of the range reported by McIntosh and Morris in sheep [6] (a similarly sized mammal to humans). This is lower than the 6.8 mL/hour calculated from 0.2% of plasma flow and a more conservative assumption for the availability of drug at target. A range of lymph flow rates is considered further in this analysis.

## S1 Table. Tissue-specific parameters used in the PBPK model

|  | **Total Volume**  **(mL)** | **Plasma Volume (mL)** | **Interstitial Volume (mL)** | **Endosomal Volume (mL)** | **Plasma flow (mL/hr)** | **Lymph flow (mL/hr)** |
| --- | --- | --- | --- | --- | --- | --- |
| **Heart** | 341.5 | 12.9 | 48.8 | 1.7 | 7751.7 | 11.5 |
| **Lung** | 2294.0 | 649.0 | 188.6 | 5.0 | 181912.5 | 24.6 |
| **Muscle** | 30078.4 | 649.0 | 3910.0 | 150.4 | 33468.6 | 277.1 |
| **Skin** | 3408.0 | 126.1 | 1125.0 | 17.0 | 11625.9 | 35.5 |
| **Adipose** | 13465.2 | 166.0 | 2289.0 | 67.3 | 11233.2 | 20.5 |
| **Bone** | 10164.4 | 142.1 | 1891.0 | 50.8 | 2590.5 | 185.8 |
| **Brain** | 1314.9 | 28.9 | 236.7 | 7.3 | 21453.3 | 0.0 |
| **Kidney** | 331.6 | 18.0 | 49.8 | 1.7 | 36402.3 | 6.8 |
| **Liver** | 2142.8 | 121.4 | 429.0 | 10.7 | 13209.9 | 55.1 |
| **GIT** | 2140.7 | 18.0 | 349.1 | 1.9 | 26997.3 | 57.5 |
| **Pancreas** | 103.7 | 3.9 | 18.0 | 0.5 | 3055.8 | 0.7 |
| **Thymus** | 6.4 | 0.4 | 1.1 | 0.0 | 353.1 | 0.0 |
| **Spleen** | 221.5 | 26.6 | 44.3 | 1.1 | 6342.6 | 0.2 |
| **Lymph Node** | 274.1 |  | 95.9 |  |  |  |
| **Other** | 4711.6 | 12.5 | 443.6 | 24.3 | 7428.3 | 60.3 |
| **Plasma** | 1149.2 |  | 0.0 |  |  |  |
| **Blood cells** | 2558.0 |  | 0.0 |  |  |  |

GIT, gastrointestinal tract

## S2 Table. Calculations of extravasation, binding, and FcRn recycling to predict drug distribution and elimination

| **Parameter** | **Value** | **Units** | **Source** |
| --- | --- | --- | --- |
| **Serum IgG Concentration** | 98.3 | µM | Average of reported range from 7 |
| **Total Endosomal FcRn** | 266 | µM | 2 |
| **FcRN-IgG Binding On Rate in Endosomes** | 0.56 | 1/(nM*hr) | 8 |
| **FcRN-IgG Binding Off Rate in Endosomes** | 23.9 | hr^-1^ | 8 |
| **Glomerular Filtration Rate** | 8402 | mL/hr | 9 |
| **Urine Production Rate** | 58.3 | mL/hr | 9 |
| **Large Pore Radius** | 22.9 | nM | 10 |
| **Small Pore Radius** | 4.4 | nM | 10 |
| **Lymph Pore Radius** | 25.0 | nM | 11 |
| **Endosomal Turnover Rate** | 0.9 | hr^-1^ | 2 |
| **Fraction Endosomal Recycling to Interstitial Space** | 0.715 |  | 8 |
| **Endosomal IgG Free Fraction Degradation Rate** | 95.6 | hr^-1^ | Fitted to match reported mAb half-life |
| **Large Pore Conductance** | 0.042 |  | 1 |
| **GSK1070806-Free hIL18 Binding On Rate** | 2.79 | 1/(nM*hr) | Measured using Biacore T1000 at 37°C |
| **GSK1070806-Free hIL18 Binding Off Rate** | 0.50 | 1/hr | Measured using Biacore T1000 at 37°C |

FcRn, neonatal Fc receptor; IgG, immunoglobulin G; hIL18, human interleukin-18; mAb, monoclonal antibody

Baseline levels of IL18 in all compartments in the model were calculated using a synthesis-degradation approach. To simulate a worst-case scenario for achieving target suppression at the site of action, all IL18 synthesis was assigned to the kidney interstitium as a zero-order reaction. IL18 degradation was assigned as a first-order degradation reaction in all compartments. The degradation rate was set to achieve an IL18 half-life of 35 hours, consistent with the measured half-life of clinically administered recombinant IL18 [7]. While this half-life seems quite long relative to the reported half-life of other cytokines, it is further supported by the observed total IL18 accumulation ratio of ~40–80 fold observed in the previous first-time-in-human study [8], which would imply 9–18-hour half-life assuming the bound complex takes on the 31 day half-life of the free mAb. Accelerated IL18-mAb complex clearance would imply a slightly longer half-life. Sensitivity analysis around IL18 half-life was performed to provide further confidence in the dose selection and is reported here.

*Pharmacokinetic validation*

A verification of the simulated plasma PK profiles was performed against previous measurements in healthy volunteers [8]. Doses of 0.008 mg/kg to 10 mg/kg were simulated and non-compartmental analysis was performed to generate comparative summary statistics. The simulated areas under the concentration-time curve and maximum observed concentrations (C_max_) values are generally in good agreement with observed values; however, they tend to skew low. This may be due to some degree of analytical skew in the original measurements as it is of note that the reported C_max_ values are higher than the theoretical maximum, calculated as the mass dose divided by human plasma volume (ie, 3 L for a 71 kg patient).

## S3 Table. Simulated PK parameters vs reported measurements in healthy volunteers

| Dose  (mg/kg) | C_max_  (ug/mL)  Simulated Reported | | AUC_0-672 hr_  (mg*h/mL)  Simulated Reported | | AUC_0-∞_  (mg*h/mL)  Simulated Reported | | Plasma half-life  (Days)  Simulated Reported | |
| --- | --- | --- | --- | --- | --- | --- | --- | --- |
| 0.008 | 0.2 | 0.1 | 0.0 | NR | 0.1 | NR | 31 | NR |
| 0.05 | 1.0 | 1.5 | 0.2 | 0.3 | 0.5 | 0.5 | 31 | 18.8 |
| 0.25 | 4.9 | 7.6 | 1.1 | 2.2 | 2.3 | 3.9 | 31 | 29.0 |
| 1 | 19.8 | 35.3 | 4.5 | 9.2 | 9.4 | 19.7 | 31 | 31.2 |
| 3 | 59.9 | 100.0 | 13.6 | 28.8 | 28.1 | 69.0 | 31 | 38.5 |
| 10 | 199.7 | 232.0 | 45.2 | 71.0 | 93.4 | 194.6 | 31 | 49.2 |

AUC, area under the concentration-time curve; C_max_, maximum observed concentration

*Baseline simulation*

In order to support dose selection, simulation of drug exposure in the kidney interstitium was performed using PBPK modeling. Required drug exposure was calculated based on IL18 suppression, which is defined as the percent change in free IL18 levels from pretreatment levels. Initial simulations were performed assuming that IL18 was generated and degraded only in the kidney and was carried to plasma by lymphatics and, to a lesser extent, diffusion into capillaries. The half-life of IL18 in the kidney was fixed (a half-life of 1 hour in the kidney), and the generation rate was adjusted to generate a plasma concentration of 835 pg/mL, which is consistent with the IL18 plasma concentrations in the 90^th^ percentile of patients with delayed graft function (DGF) [9]. The results of this simulation on receptor occupancy, free drug levels, and free IL18 levels were simulated and plotted below. Based on this scenario, >90% receptor occupancy is maintained over the 2 months following treatment. High receptor occupancies were achieved relatively quickly with 90% receptor occupancy ~50 minutes after dosing.

## S1 Fig. Simulation of drug exposure in the kidney interstitium based on IL18 levels using PBPK modeling


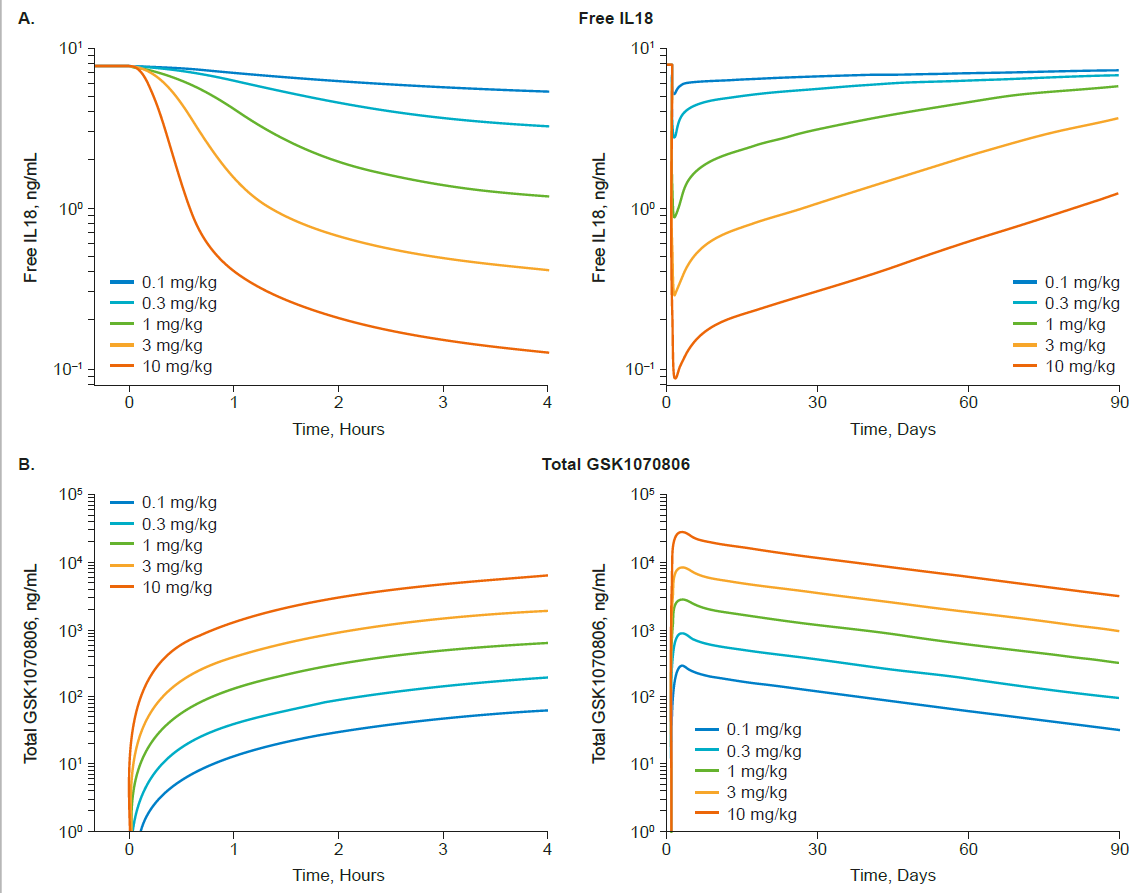


IL18, interleukin-18; PBPK, physiologically-based pharmacokinetics

*Sensitivity analysis*

Due to the nature of DGF, significant inter-patient physiological effects are expected due to damaged lymphatics, no-reflow conditions, and variation in IL18 expression. Further, we sought to address additional uncertainties in the IL18 turnover rate. A sensitivity analysis was performed against the following critical parameters: renal blood flow, renal lymph flow, IL18 levels in plasma, and IL18 turnover rate (ie, half-life). Blood and lymphatic flow rates of 1% to 300% of nominal were simulated, as well as IL18 half-lives of 1 hour to 100 hours and IL18 plasma concentrations of 100 pg/mL to 3000 pg/mL. Simulated target engagement is quite robust to blood flow, IL18 half-life, and IL18 plasma concentration, with >90% IL18 suppression achieved at 2 hours even with parameters varied 3-fold from the nominal value. Decrease in lymphatic circulation by 3-fold would result in a delay of 90% target engagement achieved from 2 hours to 3 hours.

## S2 Fig. Results of 3 mg/kg dose sensitivity analysis across key renal and IL18 physiological parameters in the model


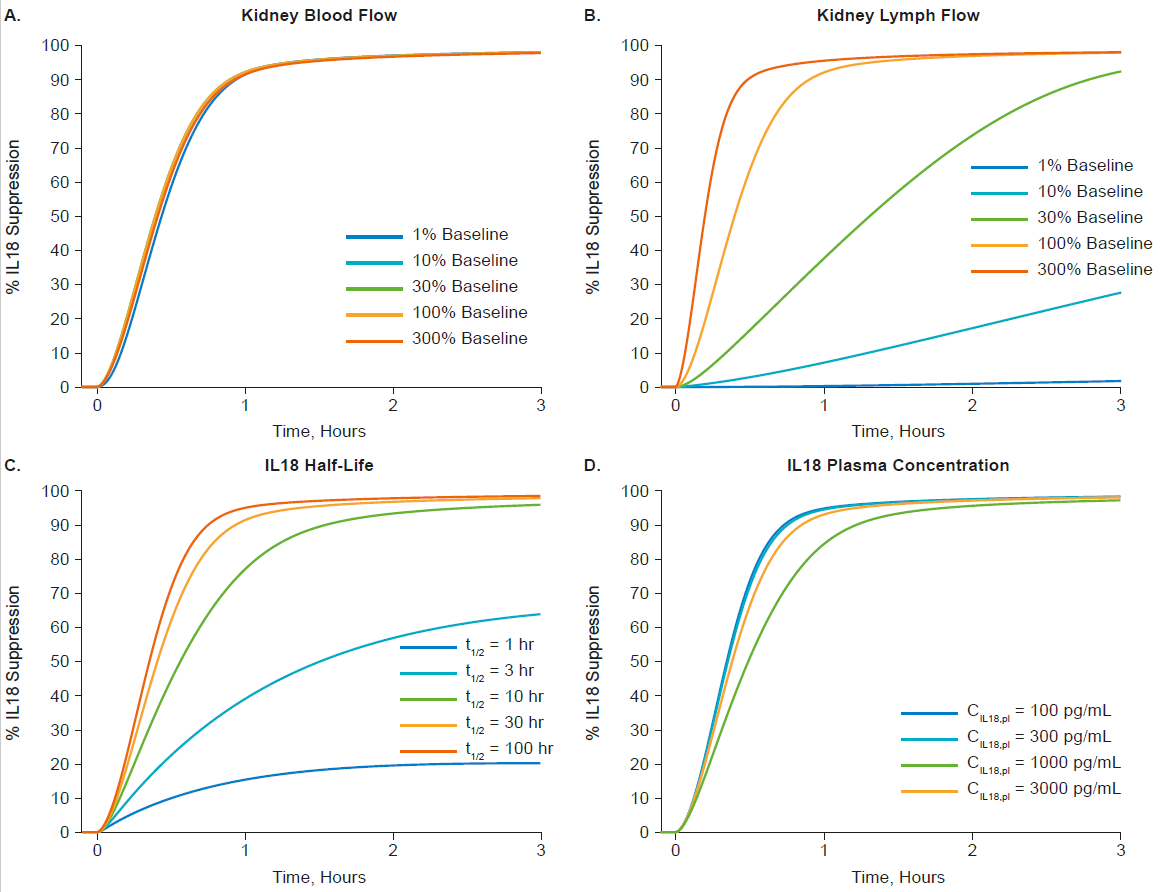


C_IL18.pl_, plasma concentration of IL18; IL18, interleukin-18; t_1/2_, terminal phase half-life

*Diffusion effects*

In the previous PBPK modeling approach, the tissue interstitium was considered as a well-mixed compartment, which is generally considered as a good assumption for PK-PD timescales. As apoptosis-mediated DGF is thought to be a highly time-sensitive process, this assumption was evaluated by calculating the expected time required for diffusion of the antibody from the vascular wall to the furthest point in the tissue (ie, the inter-capillary distance). Simulation of the average concentration profile was performed using Fick’s second law of diffusion (below), where the diffusion coefficient was set to 9E-8 cm^2^/s based on previously reported values for immunoglobulin G in vivo [10].

Fick’s second law of diffusion

$$\frac{dC}{dt}=D\frac{d^{2}C}{dx^{2}}$$

Intercapillary distances up to 600 µm were simulated. However, this is thought to be a significant overestimate, with many of the kidney tubules located adjacent to peritubular capillaries [11], suggesting tighter distances than the estimated mean 40-µm separation in human tissue [12]. These simulations indicate that the minimum concentration in the tissue should approach the wall concentration within 30 minutes for any intercapillary distance within the Krogh distance, and more likely within 5 minutes for distances of <100 µm, well within the expected diffusion range, even when considering a tortuous path around cells and extracellular matrix. It was therefore concluded that diffusion was not expected to be rate limited for the required inhibition of IL18 proximal to the tubule cells. This assumption is further relaxed by the expectation that any net fluid flow in the kidney interstitium would further aid in the distribution of the drug.

## S3 Fig. Estimated concentration gradient in tissue as a function of distance between capillaries


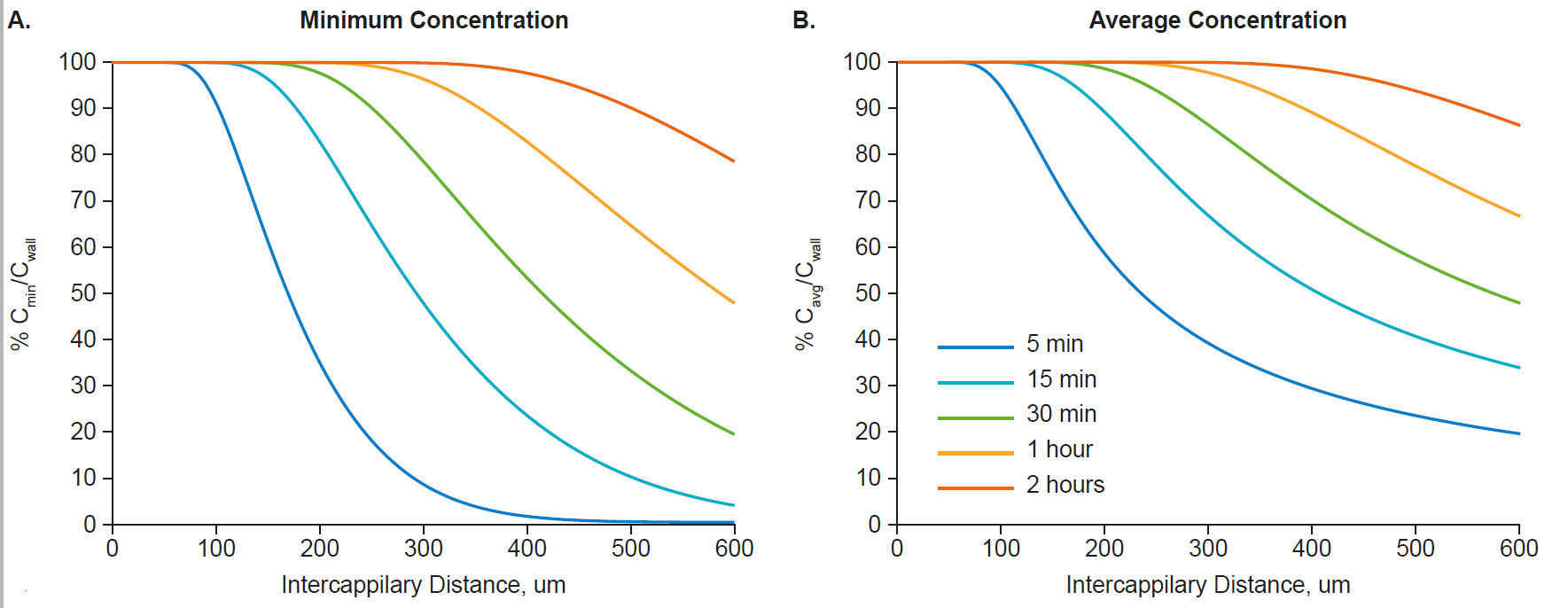


C_avg_, average observed concentration; C_min_, minimum concentration; C_wall_, concentration at the capillary wall

# S5 Supplementary Material. Estimation of background DGF rates

Contemporary historical data (n=14,887) was obtained from the National Health Service Blood and Transplant registry, limiting to years 2010–2014 inclusive. From this registry, inclusion/exclusion criteria for the present trial were used to retrieve a relevant data set of 2928 patients. Further limitation of this study population to the 4 original UK clinical sites planned for enrollment in an attempt to normalize for standard-of-care yielded 760 patients.

For this limited population of 760 patients, DGF rates were verified by each site with source documentation for accuracy: missing data were filled, enhancing confidence in DGF rates and the historical control data set as a whole. After this verification process, the overall DGF rate was 48.8% aggregated across sites and years (range 42.8–54.8%), which was highly comparable with the published rate of 49% DGF in DCD transplantation in the UK between 2005 and 2010 [13]. A sensitivity analysis confirmed no significant evolution of DGF rates over time, nor variation between sites. Overall, this process provided data supporting the use of an estimated background DGF rate of 50% in the employed single-arm Bayesian study design.

# S6 Supplementary Material S6. Statistical analyses

The primary statistical analysis was an estimate of the observed DGF rate during the study. The proportion of patients with DGF was also estimated using a logistic regression model with an intercept only term. The estimated posterior distribution for the proportion of patients with DGF was summarized using the mean and 95% credible intervals (Crl). The posterior probability that the proportion of patients experiencing DGF was <30% and 50% was also produced (with 95% Crl). A non-informative prior was used. All other endpoints were summarized descriptively where data were available. Safety data were summarized using the All Patients population, which comprised of patients who received the dose of study medication. Efficacy and PD/biomarker data were summarized using the Analysis population. For DGF-related endpoints, this was defined as patients in the All Patients population who exhibited DGF or had reached 7 days post-implant. For other endpoints, the analysis population was defined as patients having assessments at baseline and at ≥1 post-baseline time point. Serum cytokine figures were re-produced post hoc on a log scale to aid interpretation. Serum levels of IL18-GSK1070806 complex, total IL18, and IL18-BP over the study course were also plotted post hoc from data listings. Current medical conditions were also summarized post hoc from the original database collected as per protocol.

# S7 Supplementary Material. Determination of binding constants for GSK1070806 binding to recombinant human pro-IL18 and mature IL18 by surface plasmon resonance

Binding constant determinations were performed on a Biacore™ T100 platform (GE Lifesciences, Marlborough, MA). GSK1070806, recombinant human pro-IL18 and mature IL18 were prepared by GlaxoSmithKline as previously described [14]. HBS-EP buffer (GE Lifesciences, Marlborough, MA) was used for the binding experiments. Binding analyses were carried using an antibody capture method. Briefly, Protein A was immobilized on a CM5 chip by primary amine coupling in accordance with the manufacturer’s instructions. GSK1070806 was captured on the Protein A surface. After a period of stabilization, defined concentrations of analyte (recombinant human pro-IL18 or mature IL18) were passed over the antibody captured surface and binding sensorgrams were generated. After each injection of IL18 the Protein A surface was regenerated using acidic regeneration conditions (either 10 mM glycine pH 1.5, or 100 mM phosphoric acid). The regeneration removed the captured antibody, but it did not significantly impact the ability of the Protein A surface to capture antibody for another IL18 binding event. Also included in the binding analysis was a buffer injection over the captured antibody surface. This was used to double-reference the binding curves and to eliminate baseline drift and machine artefacts in accordance with Biacore best practice. Binding experiments were performed multiple times (2 to 6 independent assay runs) at 25°C (mature IL18 only) and 37°C (proIL18 and mature IL18). The data were analyzed using the Biacore T100 analysis software and was fit to the 1:1 binding model to calculate the binding constants.

## S4 Table. Binding constants (Kd) for GSK1070806

| **Human IL18** | **Binding constant (Kd)** | |
| --- | --- | --- |
|  | **25°C** | **37°C** |
| Pro-IL18 | ND | 46.7 nM |
| Mature IL18 | 16.3 pM | 46.0 pM |

ND, not determined

# S5 Table. Probability decision under the null for various sample sizes

|  | **Probability of “Go” decision** | |
| --- | --- | --- |
| Sample size, n_max_ | Type I error  (assuming true DGF rate is 50%) | Power  (if true DGF rate is 35%) |
| 25 | 0.143 | 0.659 |
| 30 | 0.139 | 0.690 |
| 35 | 0.135 | 0.716 |
| 40 | 0.138 | 0.742 |
| 45 | 0.114 | 0.747 |

DGF, delayed graft function; n_max_, maximum number of patients

# S6 Table. Summary of serious adverse events by system organ class

| **System organ class preferred term** | **Patients, n (%) N=7** |
| --- | --- |
| **Any event** | 6 (86) |
| **Infections and infestations**  Any event  Pneumonia  Urinary tract infection  Visceral leishmaniasis | 3 (43)  1 (14)  1 (14)  1 (14) |
| **Renal and urinary disorders**  Any event  End stage renal disease  Ureteric obstruction | 2 (29)  1 (14)  1 (14) |
| **Cardiac disorders**  Any event  Cardiac failure  Ventricular fibrillation | 1 (14)  1 (14)  1 (14) |
| **Gastrointestinal disorders**  Any event  Small intestinal obstruction | 1 (14)  1 (14) |
| **General disorders and administration site conditions**  Any event  Hernia | 1 (14)  1 (14) |
| **Immune system disorders**  Any event  Kidney transplant rejection | 1 (14)  1 (14) |
| **Injury, poisoning and procedural complications**  Any event  Transplant dysfunction | 1 (14)  1 (14) |
| **Reproductive system and breast disorders**  Any event  Prostatomegaly | 1 (14)  1 (14) |
| **Respiratory, thoracic and mediastinal disorders**  Any event  Respiratory arrest | 1 (14)  1 (14) |
| **Surgical and medical procedures**  Any event  Ureteral stent removal | 1 (14)  1 (14) |

# S7 Table. Summary statistics of serum GSK1070806 pharmacokinetics

| **Parameter** | **n** | **Mean** | **95% CI** | **SD** | **Median** | **Min.** | **Max**. |
| --- | --- | --- | --- | --- | --- | --- | --- |
| **AUC_∞_, mg.h/mL** | 6 | 43.27 | 27.34–59.19 | 15.18 | 40.45 | 25.30 | 61.20 |
| **AUC_o-τ_, mg.h/mL** | 7 | 36.34 | 17.40-55.29 | 20.48 | 35.10 | 2.10 | 61.00 |
| **C_max_, μg/mL** | 7 | 53.14 | 29.69-76.59 | 25.35 | 61.00 | 1.68 | 75.90 |
| **t_1/2_** | 6 | 38 | 33-43 | 5 | 38 | 31 | 44 |

AUC_∞_, area under the concentration-time curve from time zero (pre-dose) extrapolated to infinite time; CI, confidence interval; C_max_, maximum observed concentration; t_1/2_, terminal phase half-life.

# S8 Table. Urinary biomarkers of acute kidney injury (AKI)

| **Visit** | **Patient** | | | | | | | | | | | | | | | | | | | | |
| --- | --- | --- | --- | --- | --- | --- | --- | --- | --- | --- | --- | --- | --- | --- | --- | --- | --- | --- | --- | --- | --- |
| **Day** | **51** | | | **52** | | | **53** | | | **54** | | | **101** | | | **151** | | | **301** | | |
|  | **IL18** | **KIM1** | **NGAL** | **IL18** | **KIM1** | **NGAL** | **IL18** | **KIM1** | **NGAL** | **IL18** | **KIM1** | **NGAL** | **IL18** | **KIM1** | **NGAL** | **IL18** | **KIM1** | **NGAL** | **IL18** | **KIM1** | **NGAL** |
| PreOp | BLQ | 0.9 | 41 | BLQ | 1.5 | 340 | ** | ** | ** | ** | ** | ** | ** | ** | ** | ** | ** | ** | ** | ** | ** |
| 0 | 0.07 | 1.9 | 55 | 0.06 | 0.2 | 331 | 0.42 | 5.3 | 15 | ** | 0.2 | 55 | ** | ** | ** | ** | ** | ** | ** | ** | ** |
| 1 | 0.04 | 4.4 | 100 | 0.04 | 2.0 | 485 | 0.04 | 0.2 | 96 | ** | 0.3 | 29 | ** | ** | ** | 0.07 | 1.2 | 695 | ** | ** | ** |
| 2 | BLQ | 1.1 | 18 | 0.13 | 5.6 | 403 | 0.24 | 0.5 | 307 | ** | 0.2 | 105 | ** | ** | ** | 0.01 | 0.9 | 166 | ** | ** | ** |
| 3 | ** | 0.6 | 116 | 0.03 | 5.0 | 554 | 0.08 | 0.4 | 76 | ** | 0.8 | 95 | ** | ** | ** | BLQ | 0.6 | 279 | ** | ** | ** |
| 4 | ** | 0.5 | 238 | 0.04 | 5.2 | 491 | BLQ | 0.2 | 14 | ** | 0.7 | 88 | ** | ** | ** | 0.01 | 0.7 | 961 | ** | ** | ** |
| 5 | 0.01 | 1.7 | 301 | BLQ | 1.1 | 222 | 0.02 | 0.7 | 157 | ** | 1.1 | 97 | ** | ** | ** | 0.07 | 0.6 | 321 | ** | ** | ** |
| 6 | ** | ** | ** | ** | 0.6 | 340 | BLQ | 0.5 | 20 | ** | 1.0 | 307 | ** | ** | ** | BLQ | 0.5 | 611 | ** | ** | ** |
| 7 | ** | ** | ** | BLQ | 0.7 | 85 | BLQ | 1.1 | 19 | ** | 1.4 | 157 | ** | ** | ** | BLQ | 0.5 | 352 | ** | ** | ** |
| 8 | ** | ** | ** | ** | 0.8 | 144 | BLQ | 0.3 | 19 | ** | 1.9 | 83 | ** | ** | ** | BLQ | 0.4 | 967 | ** | ** | ** |
| 9 | ** | ** | ** | ** | 1.3 | 318 | ** | ** | ** | ** | 4.8 | 145 | 0.02 | 1.7 | 72 | 0.04 | 1.9 | 576 | ** | ** | ** |
| 10 | ** | ** | ** | ** | ** | ** | ** | ** | ** | ** | 1.1 | 25 | 0.01 | 1.7 | 326 | 0.03 | 2.6 | 240 | ** | ** | ** |
| 11 | ** | ** | ** | ** | ** | ** | ** | ** | ** | ** | 1.2 | 29 | BLQ | 1.7 | 221 | 0.04 | 2.3 | 256 | ** | ** | ** |
| 12 | ** | ** | ** | ** | ** | ** | ** | ** | ** | ** | 1.8 | 52 | BLQ | 1.3 | 290 | BLQ | 1.2 | 412 | ** | ** | ** |
| 13 | ** | ** | ** | ** | ** | ** | ** | ** | ** | ** | ** | ** | BLQ | 0.3 | 207 | BLQ | 0.8 | 565 | ** | ** | ** |
| 14 | ** | ** | ** | ** | ** | ** | ** | ** | ** | ** | ** | ** | BLQ | 0.7 | 513 | ** | 0.6 | 514 | ** | ** | ** |
| 15 | ** | ** | ** | ** | ** | ** | ** | ** | ** | ** | ** | ** | BLQ | ** | ** | BLQ | ** | ** | ** | ** | ** |
| 16 | ** | ** | ** | ** | ** | ** | ** | ** | ** | ** | ** | ** | 0.09 | ** | ** | BLQ | ** | ** | ** | ** | ** |
| 17 | ** | ** | ** | ** | ** | ** | ** | ** | ** | ** | ** | ** | 0.01 | ** | ** | 0.01 | ** | ** | ** | ** | ** |
| 18 | ** | ** | ** | ** | ** | ** | ** | ** | ** | ** | ** | ** | 0.01 | ** | ** | 0.04 | ** | ** | ** | ** | ** |
| 19 | ** | ** | ** | ** | ** | ** | ** | ** | ** | ** | ** | ** | BLQ | ** | ** | 0.01 | ** | ** | ** | ** | ** |
| 20 | ** | ** | ** | ** | ** | ** | ** | ** | ** | ** | ** | ** | BLQ | ** | ** | BLQ | ** | ** | ** | ** | ** |
| 21 | ** | ** | ** | ** | ** | ** | ** | ** | ** | ** | ** | ** | ** | ** | ** | 0.01 | 3.1 | 447 | ** | ** | ** |
| 22 | ** | ** | ** | ** | ** | ** | ** | ** | ** | ** | ** | ** | BLQ | ** | ** | ** | ** | ** | ** | ** | ** |
| 23 | ** | ** | ** | ** | ** | ** | ** | ** | ** | ** | ** | ** | BLQ | ** | ** | ** | ** | ** | ** | ** | ** |
| 24 | ** | ** | ** | ** | ** | ** | ** | ** | ** | ** | ** | ** | BLQ | ** | ** | ** | ** | ** | ** | ** | ** |
| 25 | ** | ** | ** | ** | ** | ** | ** | ** | ** | ** | ** | ** | 0.01 | ** | ** | ** | ** | ** | ** | ** | ** |
| 26 | ** | ** | ** | ** | ** | ** | ** | ** | ** | ** | ** | ** | 0.03 | ** | ** | ** | ** | ** | ** | ** | ** |
| 27 | ** | ** | ** | ** | ** | ** | ** | ** | ** | ** | ** | ** | 0.01 | ** | ** | ** | ** | ** | ** | ** | ** |
| 28 | ** | ** | ** | ** | ** | ** | ** | ** | ** | ** | ** | ** | 0.01 | ** | ** | ** | ** | ** | ** | ** | ** |
| 29 | ** | ** | ** | ** | ** | ** | ** | ** | ** | ** | ** | ** | 0.01 | ** | ** | ** | ** | ** | ** | ** | ** |
| 30 | BLQ | 0.5 | 4 | BLQ | 0.1 | 5 | BLQ | 0.6 | 11 | ** | 2.4 | 36 | BLQ | 1.7 | 276 | ** | 1.0 | 735 | ** | ** | ** |
| 31 | ** | ** | ** | ** | ** | ** | ** | ** | ** | ** | ** | ** | 0.06 | ** | ** | ** | ** | ** | ** | ** | ** |
| 33 | ** | ** | ** | ** | ** | ** | ** | ** | ** | ** | ** | ** | 0.02 | 4.7 | 117 | ** | ** | ** | ** | ** | ** |
| 86 | ** | ** | ** | ** | ** | ** | ** | ** | ** | ** | ** | ** | ** | ** | ** | ** | ** | ** | ** | ** | ** |
| 90 | BLQ | 0.5 | 7 | BLQ | 0.9 | 95 | ** | 0.7 | 16 | ** | 2.6 | 59 | ** | 8.0 | 150 | ** | 1.3 | 490 | ** | 0.5 | 235 |
| 180 | ** | 0.4 | 86 | ** | 1.0 | 69 | ** | 1.1 | 63 | ** | 0.5 | 97 | ** | 1.2 | 348 | ** | 1.0 | 563 | ** | ** | ** |
| 360 | ** | 0.2 | 51 | ** | 0.6 | 61 | ** | 0.3 | 61 | ** | 1.1 | 68 | ** | 1.9 | 111 | ** | ** | ** | ** | ** | ** |

Values at Hospital Discharge are boxed in blue. Elevated values are shaded: IL18 >0.1 ng/mL; KIM1 >2.37 ng/ml; NGAL >153 ng/mL

**, not determined; BLQ, below limit of quantification; IL18, interleukin-18, KIM1, Kidney Injury Molecule-1; NGAL, neutrophil gelatinase-associated lipocalin; PreOp, prior to transplantation. All units are represented as ng/mL

# S4 Fig. Serum creatinine levels over the 12-month course of study


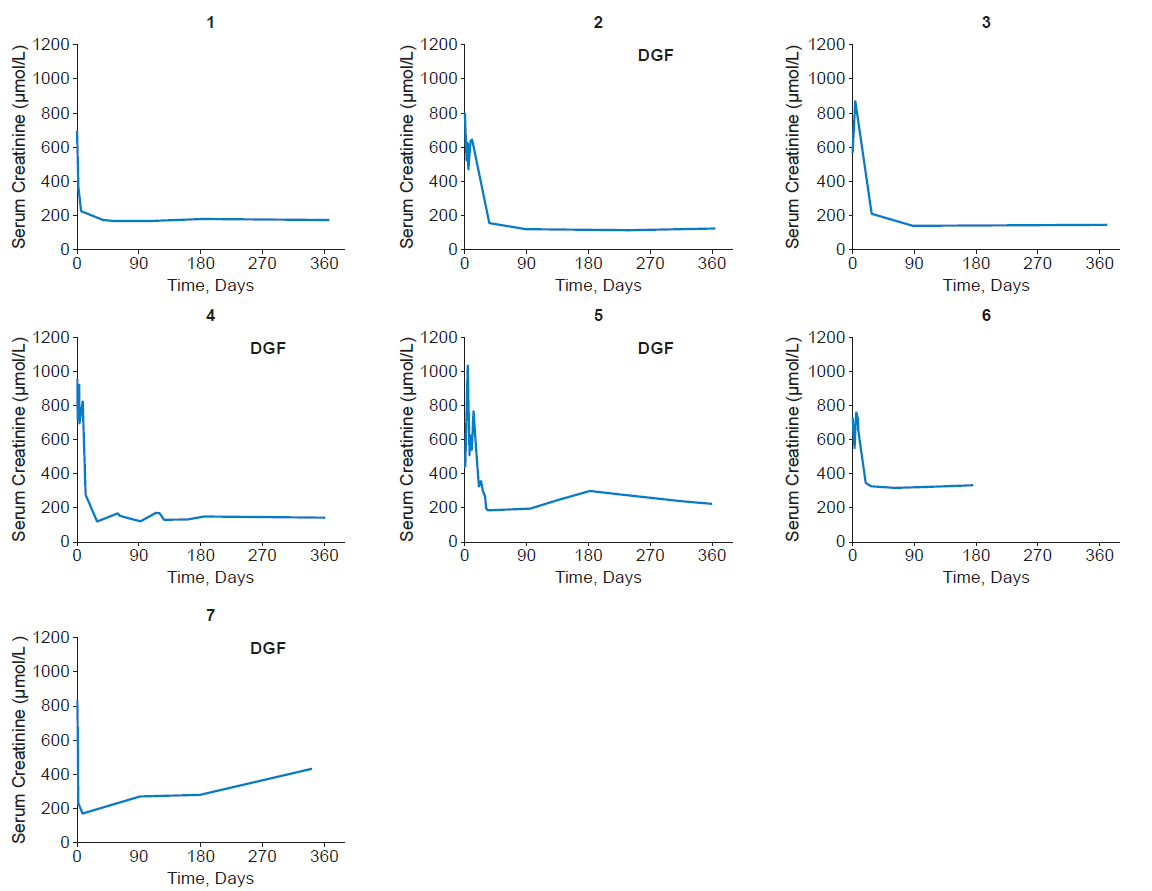


DGF, delayed graft function

# S5 Fig. Serum cytokine levels over the study course. (A) IL1β, (B) IL2, (C) IL8, (D) MCP1, (E) MIP1β, (F) TNFα


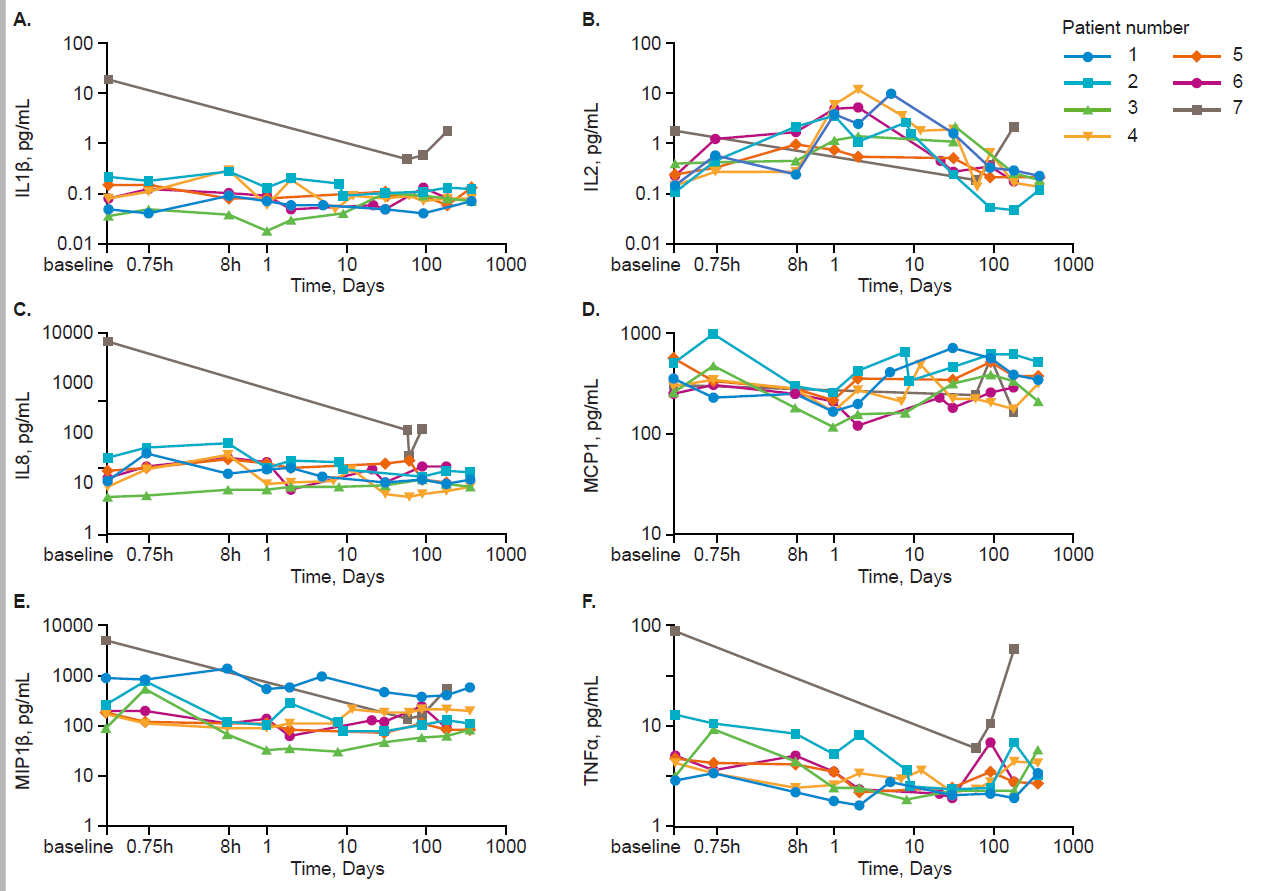


IL2 levels were elevated in all patients at baseline and decreased over the study course. IL8, IL1β, MIP1β, and TNFα levels were low and largely unchanged in all patients except in Patient 301, in whom they were elevated at baseline and decreased by Day 59 before beginning to increase.

IL1β, interleukin-1β; IL2, interleukin-2; IL8, interleukin-8; MCP1, monocyte chemoattractant protein 1; MIP1β, macrophage inflammatory protein 1β; TNFα, tumor necrosis factor-α

Figures produced post hoc

# S6 Fig. Hematoxylin and eosin staining of samples from Patients 2 (A), 4 (B) and 5 (C)


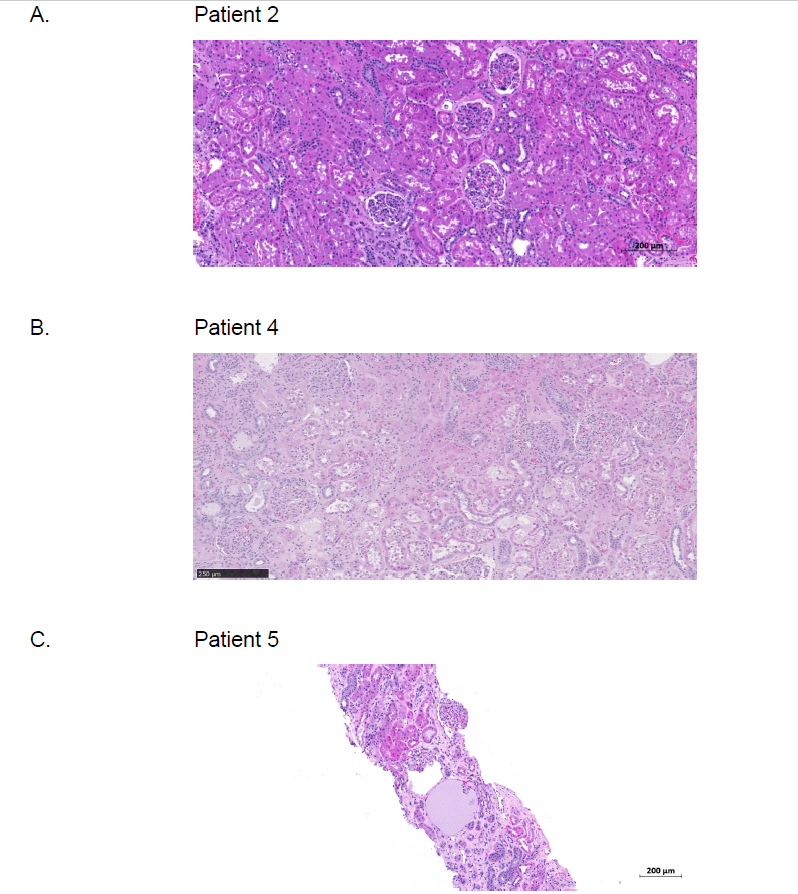


Hematoxylin and eosin staining showing moderate tubular vacuolation and basement membrane thickening in DGF Patients 2 and 4, and extensive basement membrane thickening, increased connective tissue, and dilated Bowman’s space in DGF Patient 5

# S7 Fig. Anti-IL18 immunohistochemistry staining in a non-dosed and non-transplanted control kidney

GSK1070806 only


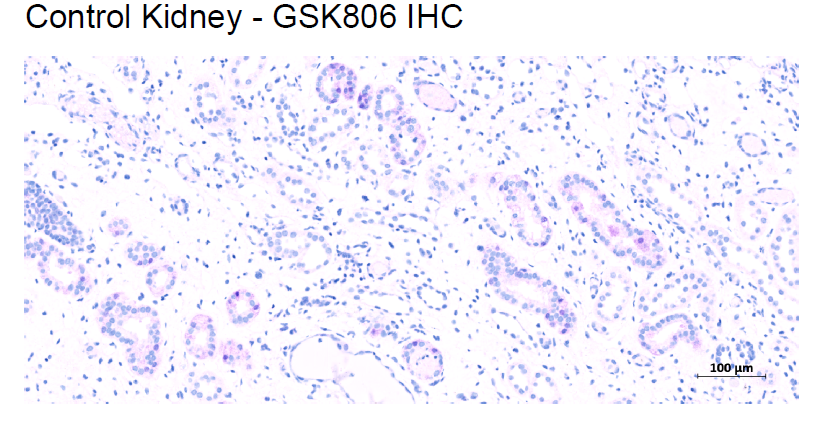


Anti-idiotype antibody only

Primary GSK1070806; secondary anti-idiotype

Cadaveric kidney (not suitable for transplantation) obtained from the National Disease Research Interchange (EBA012216A) stained with anti-GSK1070806 idiotypic antibody alone or using GSK1070806 as primary antibody for detection of IL18.

# References

1. Sepp A, Berges A, Sanderson A, Meno-Tetang G. Development of a physiologically based pharmacokinetic model for a domain antibody in mice using the two-pore theory. J Pharmacokinet Pharmacodyn. 2015;42: 97-109.
2. Sepp A, Meno-Tetang G, Weber A, Sanderson A, Schon O, Berges A. Computer-assembled cross-species/cross-modalities two-pore physiologically based pharmacokinetic model for biologics in mice and rats. J Pharmacokinet Pharmacodyn. 2019;46: 339-359.
3. Graf JF, Scholz BJ, Zavodszky MI. BioDMET: a physiologically based pharmacokinetic simulation tool for assessing proposed solutions to complex biological problems. J Pharmacokinet Pharmacodyn. 2012;39: 37-54.
4. Garg A, Balthasar JP. Physiologically-based pharmacokinetic (PBPK) model to predict IgG tissue kinetics in wild-type and FcRn-knockout mice. J Pharmacokinet Pharmacodyn. 2007;34: 687-709.
5. Swartz MA. The physiology of the lymphatic system. Adv Drug Deliv Rev. 2001;50: 3-20.
6. McIntosh GH, Morris B. The lymphatics of the kidney and the formation of renal lymph. J Physiol. 1971;214: 365-376.
7. Robertson MJ, Mier JW, Logan T, Atkins M, Koon H, Koch KM, et al. Clinical and biological effects of recombinant human interleukin-18 administered by intravenous infusion to patients with advanced cancer. Clin Cancer Res. 2006;12: 4265-4273.
8. Mistry P, Reid J, Pouliquen I, McHugh S, Abberley L, DeWall S, et al. Safety, tolerability, pharmacokinetics, and pharmacodynamics of single-dose antiinterleukin-18 mAb GSK1070806 in healthy and obese subjects. Int J Clin Pharmacol Ther. 2014;52: 867-879.
9. Hall IE, Doshi MD, Poggio ED, Parikh CR. A comparison of alternative serum biomarkers with creatinine for predicting allograft function after kidney transplantation. Transplantation. 2011;91: 48-56.
10. Pluen A, Boucher Y, Ramanujan S, McKee TD, Gohongi T, di Tomaso E, et al. Role of tumor-host interactions in interstitial diffusion of macromolecules: cranial vs. subcutaneous tumors. Proc Natl Acad Sci U S A. 2001;98: 4628-4633.
11. Lemley KV, Kriz W. Anatomy of the renal interstitium. Kidney Int. 1991;39: 370-381.
12. Frietas RA. Nanomedicine: Landes Bioscience; 1999.
13. Summers DM, Johnson RJ, Hudson A, Collett D, Watson CJ, Bradley JA. Effect of donor age and cold storage time on outcome in recipients of kidneys donated after circulatory death in the UK: a cohort study. Lancet. 2013;381: 727-734.
14. Kirkpatrick RB, Grooms M, Wang F, Fenderson H, Feild J, Pratta MA, et al. Bacterial production of biologically active canine interleukin-1beta by seamless SUMO tagging and removal. Protein Expr Purif. 2006;50: 102-110.
