# Supplementary material for: A pilot study evaluating GSK1070806 inhibition of interleukin-18 in renal transplant delayed graft function
Source: PLoS One. 2021 Mar 8;16(3):e0247972. doi: 10.1371/journal.pone.0247972 (PMC7939287; doi:10.1371/journal.pone.0247972)
Supplement: S2 File — (PDF) [file pone.0247972.s003.pdf]

TITLE PAGE

Division: Worldwide Development
Information Type: Clinical Protocol

**Title:**	A Phase 2 Pilot, Multicenter, Single Arm Study to Evaluate the Efficacy, Safety, Tolerability, and Pharmacokinetics of GSK1070806 plus Standard of Care for the Prevention of Delayed Graft Function in Adult Subjects After Renal Transplantation

Compound Number: GSK1070806

Development Phase II

Effective Date: 30-NOV-2015

Author(s): PPD

SPONSOR SIGNATORY:

PPD

30 Nov 2015

Laura Demopoulos, MD **Date:**

Director – HF DPU, MPC-TAU

MEDICAL MONITOR/SPONSOR INFORMATION PAGE

Medical Monitor/SAE Contact Information:

**Role**	Name	Day Time Phone Number and email address	After-hours Phone/Cell/ Pager Number	Fax Number	Site Address
Primary Medical Monitor	PPD				GSK Upper Merion, 709 Swedeland Road, King of Prussia, PA 19406
Secondary Medical Monitor					GSK Upper Merion, 709 Swedeland Road, King of Prussia, PA 19406

Sponsor Legal Registered Address:

GlaxoSmithKline Research & Development Limited
980 Great West Road
Brentford
Middlesex, TW8 9GS
UK

In some countries, the clinical trial sponsor may be the local GlaxoSmithKline Affiliate Company (or designee). If applicable, the details of the alternative Sponsor and contact person in the territory will be provided to the relevant regulatory authority as part of the clinical trial application.

Regulatory Agency Identifying Number(s): 2015-002812-33

INVESTIGATOR PROTOCOL AGREEMENT PAGE

- I confirm agreement to conduct the study in compliance with the protocol.
- I acknowledge that I am responsible for overall study conduct. I agree to personally conduct or supervise the described study.
- I agree to ensure that all associates, colleagues and employees assisting in the conduct of the study are informed about their obligations. Mechanisms are in place to ensure that site staff receives the appropriate information throughout the study.

Investigator Name: ______________________________

____ ______________________

Investigator Signature Date

TABLE OF CONTENTS

PAGE

1. PROTOCOL SYNOPSIS FOR STUDY 204824 8

2. INTRODUCTION 10
 2.1. Overview 10
 2.2. Background 10

3. OBJECTIVE(S) AND ENDPOINT(S) 12

4. STUDY DESIGN 14
 4.1. Overall Design 14
 4.2. Treatment Arms and Duration 15
 4.3. Type and Number of Subjects 15
 4.4. Design Justification 15
 4.5. Dose Justification 15
 4.6. Benefit:Risk Assessment 16
  4.6.1. Risk Assessment 17
  4.6.2. Benefit Assessment 22
  4.6.3. Overall Benefit/Risk Conclusion 22

5. SELECTION OF STUDY POPULATION AND WITHDRAWAL CRITERIA 22
 5.1. Inclusion Criteria 22
 5.2. Exclusion Criteria 24
 5.3. Screening/Baseline/Run-in Failures 26
 5.4. Withdrawal/Stopping Criteria 27
  5.4.1. Liver Chemistry Management 27
  5.4.2. QTc Evaluation Criteria 28
 5.5. Safety Interim Analysis 28
 5.6. Subject and Study Completion 29

6. STUDY TREATMENT 29
 6.1. Investigational Product and Other Study Treatment 29
 6.2. Treatment Assignment 29
 6.3. Blinding 29
 6.4. Packaging and Labeling 29
 6.5. Preparation/Handling/Storage/Accountability 30
 6.6. Compliance with Study Treatment Administration 30
 6.7. Treatment of Study Treatment Overdose 30
 6.8. Treatment after the End of the Study 31
 6.9. Lifestyle and/or Dietary Restrictions 31
 6.10. Concomitant Medications and Non-Drug Therapies 31
  6.10.1. Standard Immunosuppressive Therapy 31
  6.10.2. Management of low white cell count 31
  6.10.3. Graft Dysfunction/Graft rejection 32
  6.10.4. Prophylaxis and Surveillance for Infection 32
   6.10.4.1. Pneumocystis and Fungal Infection Prophylaxis 32
   6.10.4.2. Cytomegalovirus Infection 32
   6.10.4.3. BK virus 33

6.11. Prohibited Medications and Non-Drug Therapies....33
6.11.1. Vaccines....33
6.11.2. Concomitant Medications....33

7. STUDY ASSESSMENTS AND PROCEDURES....33
7.1. Time and Events Table....35
7.2. Screening and Critical Baseline Assessments....37
7.3. Efficacy....37
7.4. Safety....37
7.4.1. Adverse Events (AE) and Serious Adverse Events (SAEs)....37
7.4.1.1. Time period and Frequency for collecting AE and SAE information....38
7.4.1.2. Method of Detecting AEs and SAEs....38
7.4.1.3. Follow-up of AEs and SAEs....38
7.4.1.4. Cardiovascular and Death Events....39
7.4.1.5. Regulatory Reporting Requirements for SAEs....39
7.4.2. Pregnancy....39
7.4.3. Physical Exams....40
7.4.4. Vital Signs....40
7.4.5. Electrocardiogram (ECG)....40
7.4.6. Clinical Safety Laboratory Assessments....40
7.4.7. Immunogenicity....41
7.5. Pharmacokinetics....42
7.5.1. Blood Sample Collection and Analysis....42
7.5.2. Renal Graft Biopsies....42
7.6. Mechanistic Biomarker(s)/Pharmacodynamic Markers....43
7.6.1. Cytokines....43
7.6.2. Renal Graft Biopsies....43
7.6.3. Biomarkers relating to acute rejection....43
7.6.4. Clinical Biomarkers....43
7.6.4.1. Urinary biomarkers of injury....43
7.6.4.2. Renal preservation fluid sampling....44
7.6.5. Circulating cellular phenotype (Cambridge only)....44

8. DATA MANAGEMENT....44

9. STATISTICAL CONSIDERATIONS AND DATA ANALYSES....45
9.1. Hypotheses....45
9.2. Sample Size Considerations....45
9.2.1. Sample Size Assumptions....45
9.2.2. Sample Size Sensitivity....46
9.2.3. Sample Size Re-estimation or Adjustment....47
9.3. Data Analysis Considerations....47
9.3.1. Analysis Populations....47
9.3.2. Interim Analysis....48
9.4. Key Elements of Analysis Plan....49
9.4.1. Primary Analyses....49
9.4.2. Secondary Analyses....50
9.4.3. Other Analyses....50
9.4.4. Safety Analyses....50
9.4.5. Pharmacokinetic Analyses....50

10. STUDY GOVERNANCE CONSIDERATIONS 50
10.1. Posting of Information on Publicly Available Clinical Trial Registers 50
10.2. Regulatory and Ethical Considerations, Including the Informed Consent Process 50
10.3. Quality Control (Study Monitoring) 51
10.4. Quality Assurance 52
10.5. Study and Site Closure 52
10.6. Records Retention 52
10.7. Provision of Study Results to Investigators, Posting of Information on Publically Available Clinical Trials Registers and Publication 53

11. REFERENCES 54

12. APPENDICES 59
12.1. Appendix 1: Abbreviations and Trademarks 59
12.2. Appendix 2: Liver Safety Required Actions and Follow up Assessments 60
12.3. Appendix 3: Definition of and Procedures for Recording, Evaluating, Follow-Up and Reporting of Adverse Events 63
12.3.1. Definition of Adverse Events 63
12.3.2. Definition of Serious Adverse Events 64
12.3.3. Definition of Cardiovascular Events 65
12.3.4. Recording of AEs and SAEs 66
12.3.5. Evaluating AEs and SAEs 66
12.3.6. Reporting of SAEs to GSK 67
12.4. Appendix 4: Modified List of Highly Effective Methods for Avoiding Pregnancy in FRP and Collection of Pregnancy Information 69
12.4.1. Modified List of Highly Effective Methods for Avoiding Pregnancy in Females of Reproductive Potential (FRP) 69
12.4.2. Collection of Pregnancy Information 69

1. PROTOCOL SYNOPSIS FOR STUDY 204824

Rationale

GSK1070806 is an anti-IL18 IgG_1 monoclonal antibody, which binds to the IL-18 cytokine and thereby inhibits signalling through the IL-18 receptor. In post renal transplantation patients urinary IL-18 levels are elevated, correlating with the incidence of delayed graft function (DGF). Signalling downstream of IL-18 has also been implicated in renal transplant rejection.

Objective(s)/Endpoint(s)

Objectives	Endpoints
**Primary**	
• To assess the frequency of delayed graft function (DGF) in donation after circulatory death (DCD) renal transplant recipients treated with GSK1070806.	• Proportion of subjects requiring dialysis during the first 7 days post transplant, (excluding requirement for dialysis due to hyperkalemia within first 24 post-operative hours).
**Secondary**	
• To assess graft function in DCD renal transplant recipients treated with GSK1070806.	• Serum creatinine at baseline and over time post transplant.<br>• Urine output at baseline and over time post transplant.<br>• Proportion of subjects in the first 7 days with:<br>– Primary Non Function<br>– Functional DGF<br>– Intermediate Graft Function<br>– Immediate Graft Function
• To assess the effect of GSK1070806 on acute rejection risk, and rejection/PD biomarkers.	• Proportion of subjects with episodes of biopsy-proven acute rejection.<br>• Rejection biomarkers/ PD markers (including serum IP-10 and Mig) at baseline and over time post transplant.
• To assess the effect of GSK1070806 on dialysis dependency and graft survival.	• Number of dialysis events per patient in the first 30 days post transplant.<br>• Proportion of subjects who are dialysis-independent at visits up to 12 months post transplant.
• To assess the safety and tolerability of GSK1070806 in renal transplant recipients.	• AEs and SAEs<br>– Clinical laboratory values<br>– Vital signs

Objectives	Endpoints
	– Frequency, type and severity of infections
• To assess the pharmacokinetics of GSK1070806 in renal transplant recipients.	• Serum concentrations of GSK1070806 over time, and derived pharmacokinetic parameters if feasible (AUC, Cmax).
• To assess the effect of GSK1070806 administration on serum IL-18 levels.	• Free, total, and GSK1070806 bound IL-18 at baseline and over time post transplant.
• To determine immunogenicity of GSK1070806 in renal transplant recipients.	• Frequency of anti-drug antibodies (ADAs) before and after GSK1070806 administration.<br>• ADA titers.

Overall Design

Study 204824 is a phase 2, pilot, multicenter, single arm Bayesian sequential design study to evaluate the efficacy, safety, tolerability and pharmacokinetics of GSK1070806 in patients undergoing renal transplantation.

The primary endpoint is frequency of DGF defined by a requirement for dialysis (except as needed for hyperkalaemia during the first 24 hours) within the first 7 days post transplantation. The trial utilizes a Bayesian sequential design to test whether GSK1070806 lowers the frequency of DGF. As secondary objectives the protocol assesses the safety and tolerability of GSK1070806 in renal transplantation, graft function/survival (serum creatinine, urine output, dialysis events), the impact on indicators of acute rejection, as well as GSK1070806 pharmacokinetics, pharmacodynamics and immunogenicity.

Treatment Arms and Duration

Participants will receive a single intravenous (IV) infusion of GSK1070806 administered prior to kidney allograft reperfusion, and will be followed for 12 months post dose.

Type and Number of Subjects

Adult subjects (18-75 years of age). Dialysis-dependent, recipient of first-time, single kidney-only, Donation after Circulatory Death (DCD) transplants, receiving a planned immunosuppressive standard of care comprised of basiliximab, mycophenolate mofetil (MMF) or azathioprine, tacrolimus and corticosteroids. The number of subjects to be enrolled will be up to approximately 40.

2. INTRODUCTION

2.1. Overview

GSK1070806 is an anti-IL18 IgG_1 monoclonal antibody, which binds to the IL-18 cytokine and thereby inhibits signalling through the IL-18 receptor. In patients post renal transplantation, urinary IL-18 levels may be elevated, correlating with the incidence of delayed graft function (DGF). Signalling downstream of IL-18 has also been implicated in renal allograft rejection.

Recipients of DCD renal allografts will be administered a single infusion of GSK1070806 prior to reperfusion of the transplanted kidney, to test the hypothesis that inhibition of IL-18 can reduce the rate of DGF (primary endpoint) and episodes of graft rejection (secondary endpoints). Historic DGF rates and a Bayesian statistical approach have enabled this single arm interventional approach.

2.2. Background

Acute kidney injury (AKI) complicates 5-10% of hospital admissions, and an increasing body of evidence shows that these acute insults are associated with short- and long-term increases in healthcare cost, morbidity and mortality [Rewa 2014, Ryden 2014, Lewington 2013]. Survivors of AKI experience a risk of new chronic kidney disease of 25.8 cases per 100 patient years, and a risk of end stage renal disease of 8.6 cases per 100 patient years, which is also proportional to the severity of the AKI [Coca 2014]. Accordingly, treatments affording renal protection in the acute setting are of significant interest.

Delayed graft function (DGF) after renal transplantation is a niche manifestation of AKI, defined as the failure of a transplanted kidney to function immediately in the wake of transplantation. Patients affected by DGF require dialysis pending the onset of renal function, and compared to those without DGF are at increased risk of graft failure at one year [Moers 2009, Tapiawala 2010]. Depending upon known risk factors (principally donor age, cold ischemic time, and whether organ donation follows brain death (DBD) or circulatory death (DCD)) the risk of DGF is between 25 and 50% [Summers 2013]. Ischemic renal injury is the consequence of processes that result directly from cellular hypoxia (depletion of ATP, mitochondrial leakage, free radical toxicity, loss of membrane potentials) and from immune amplification of injury (stimulation of TLRs, cytokine release, complement activation, neutrophil and monocyte chemotaxis).

IL-18 is an inflammatory cytokine secreted in response to cellular injury or inflammatory stimulus [Gu 1997]. Transcriptional control of IL-18 is uniquely regulated such that stores are available at rest for immediate release, which is dependent on cleavage from pro-IL-18 by caspase-1 [Puren 1999, Tone 1997]. IL-18 release then signals proximally in the pro-inflammatory cascade, inducing synthesis of IFNγ, TNFα, IL-6, IL-8, and IL-1 amongst other cytokines [Puren 1999, Fortin 2009]. In addition to a role in activation of innate and adaptive immunity, IL-18 has been shown to be directly cytotoxic to renal tubular epithelial cells *in vitro* [Zhang 2011].

Role of IL-18 in DGF: In large clinical cohorts, early rises in urinary IL-18 levels in the first few hours after renal insults correlate closely with the subsequent development of AKI both in native [Parikh 2011] and transplanted [Hall 2010] kidneys, and predict long term dysfunction [Hall 2012, Coca 2014]. Furthermore, emerging data has correlated IL-18 levels measured in organ preservation fluids with post-operative graft dysfunction. More direct evidence of a role in pathogenesis is derived from rodent models of experimental ischemic renal injury, in which disruption of IL-18 signalling by gene deletion, exogenous administration of neutralizing antiserum, genetic knock-in or exogenous administration of the IL-18 binding protein (IL-18BP, a neutralizing decoy receptor) are all protective [Melnikov 2001, Melnikov 2002, He 2008, Wu 2008]. However, some rodent studies have demonstrated no, or non-statistically significant, benefit of inhibiting IL-18 [Daemen 2001; Shigeoka 2010].

Role of IL-18 in allograft rejection: Renal biopsy samples from patients experiencing acute rejection have demonstrated strong staining for IL-18, and serum IL-18 levels are highly elevated compared with controls [Striz 2005]. Furthermore, rodent studies using a mismatched cardiac allograft model demonstrated enhanced graft survival when subject to IL-18 neutralization [Dudler 2007]. IL-18 was originally identified for its role in inducing IFNγ [Okamura 1995], which is strongly implicated in Th1 polarization. Acute cell-mediated rejection has been defined as a CD4+ Th1 mediated process [Issa 2010], where elevated levels of the downstream Th1 chemokine IP-10 have been shown to predict acute rejection episodes [Field 2014, Zhang 2014, Rabant 2015]. IP-10 inhibition was capable of prolonging graft survival in mismatched rodent transplantation models [Hancock 2001], and in GSK1070806 clinical studies a reduction in IP-10 has been a key pharmacodynamic readout [see Investigators' Brochure]. Thus, it is reasonable to hypothesize that neutralization of IL-18 causing downstream reductions in IP-10 levels may protect allografts from rejection.

Clinical experience with IL-18 inhibitors**:** Recognition that IL-18 may play an important role in various diseases has led to the development of investigational therapies targeting IL-18 or its signalling pathways, which have been well tolerated in healthy subjects and patients with RA, Crohn's disease, and plaque psoriasis [Tak, 2006; Mistry, 2014].

In volunteers, single intravenous infusion doses of the anti-IL18 monoclonal antibody, GSK1070806, were well tolerated in both healthy and obese subjects. No differences in infections, immune system disorders or haematologic abnormalities were observed following single intravenous GSK1070806 infusions up to 10 mg/kg in 57 subjects when compared with placebo. Four subjects had SAEs (head injury, mild pericarditis [placebo group], kidney stones, pneumothorax) that were all considered unrelated to study treatment by the Investigator [Mistry, 2014].

GSK1070806 was also evaluated in subjects with type 2 diabetes, in which two repeat doses of GSK1070806, administered 28 days apart, were well-tolerated. The most frequently reported AE was nasopharyngitis which occurred in 11 subjects. Headache, diarrhea and hypertension were the second most frequently reported AEs, which occurred in 5 subjects each. None of the reported AEs was considered related to GSK1070806. The most frequently reported MedDRA system organ class was 'Infections and Infestations'. Adverse events in this category were reported in 15 subjects (N=37 subjects

total), 6/12 on placebo, 4/13 following 0.25 mg/kg GSK1070806 administration, and 5/12 following 5.0 mg/kg GSK1070806 administration.

3. OBJECTIVE(S) AND ENDPOINT(S)

Objectives	Endpoints
**Primary**	
• To assess the frequency of delayed graft function (DGF) in donation after circulatory death (DCD) renal transplant recipients treated with GSK1070806.	• Proportion of subjects requiring dialysis during the first 7 days post transplant, (excluding requirement for dialysis due to hyperkalemia within first 24 post-operative hours).
**Secondary**	
• To assess graft function in DCD renal transplant recipients treated with GSK1070806.	• Serum creatinine at baseline and over time post transplant.<br>• Urine output at baseline and over time post transplant.<br>• Proportion of subjects in the first 7 days with:<br>– Primary Non Function<br>– Functional DGF<br>– Intermediate Graft Function<br>– Immediate Graft Function
• To assess the effect of GSK1070806 on acute rejection risk, and rejection/PD biomarkers.	• Proportion of subjects with episodes of biopsy-proven acute rejection.<br>• Rejection biomarkers/ PD markers (including serum IP-10 and Mig) at baseline and over time post transplant.
• To assess the effect of GSK1070806 on dialysis dependency and graft survival.	• Number of dialysis events per patient in the first 30 days post transplant.<br>• Proportion of subjects who are dialysis-independent at visits up to 12 months post transplant.
• To assess the safety and tolerability of GSK1070806 in renal transplant recipients.	• AEs and SAEs<br>– Clinical laboratory values<br>– Vital signs<br>– Frequency, type and severity of infections
• To assess the pharmacokinetics of GSK1070806 in renal transplant recipients.	• Serum concentrations of GSK1070806 over time, and derived pharmacokinetic parameters if feasible (AUC, Cmax).

Objectives	Endpoints
• To assess the effect of GSK1070806 administration on serum IL-18 levels.	• Free, total, and GSK1070806 bound IL-18 at baseline and over time post transplant.
• To determine immunogenicity of GSK1070806 in renal transplant recipients.	• Frequency of anti-drug antibodies (ADAs) before and after GSK1070806 administration.<br>• ADA titers.
**Exploratory**	
• To assess the impact of GSK1070806 on urine biomarkers of renal injury.	• Serial measurements of urinary biomarkers, including KIM-1, NGAL and IL-18, at baseline and over time post transplant.
• To assess IL-18 target engagement in the kidney.	• GSK1070806 and IL-18 levels within renal graft biopsies, as assessed by immunohistochemistry.
• To assess the relation between IL-18 levels in renal preservation fluid and graft function.	• Level of IL-18 in renal preservation fluid.
• To assess the impact of GSK1070806 administration on IL-18 binding protein levels.	• Serum IL-18 binding protein at baseline and over time post transplant.
• To assess the humoral and cellular response to the transplanted organ in the presence of GSK1070806.	• Serum cytokine levels at baseline and over time post transplant.<br>• Histological analysis of biopsies with respect to cellular infiltrate.
• To assess health outcomes in renal transplant recipients following GSK1070806 administration.	• Length of hospital stay<br>• Re-hospitalization

4. STUDY DESIGN

4.1. Overall Design

Figure 1 Study Design

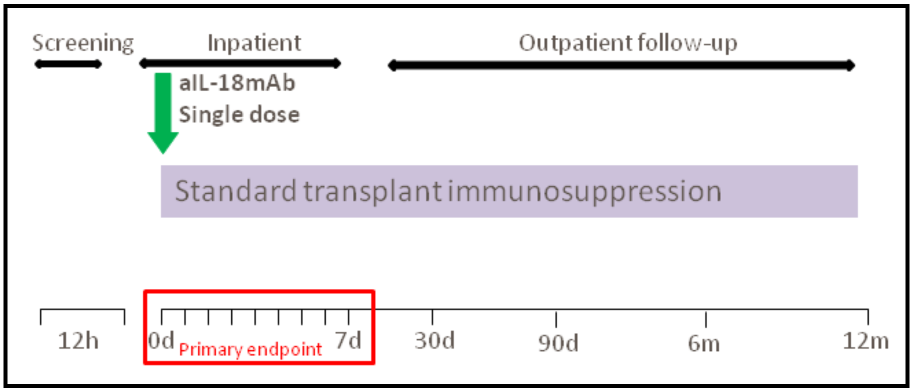

Study 204824 is a phase 2, pilot, multicenter, single arm Bayesian sequential design study to evaluate the efficacy, safety, tolerability and pharmacokinetics of GSK1070806 in patients undergoing renal transplantation.

The primary endpoint is frequency of DGF defined by a requirement for dialysis (except as needed for hyperkalaemia during the first 24 hours) within the first 7 days post transplantation. The trial utilizes a Bayesian sequential design [Section 9] to test whether GSK1070806 lowers the frequency of DGF. As secondary objectives the protocol assesses the safety and tolerability of GSK1070806 in renal transplantation, graft function/survival (serum creatinine, urine output, dialysis events), the impact on indicators of acute rejection, as well as GSK1070806 pharmacokinetics, pharmacodynamics and immunogenicity.

All subjects enrolled will have been evaluated by the investigative site's transplant program, and be considered suitable for transplantation by the institution's eligibility criteria. Informed consent will be obtained from all subjects before screening. Donation after circulatory death (DCD) transplantation is an unplanned activity, thus screening will occur on an emergency basis in the hours before transplantation using available clinical data.

Inclusion/exclusion criteria have been designed to exclude patients with heightened risk of infection, given that GSK1070806 (anti-IL18 monoclonal antibody) in this protocol amounts to an additional immunosuppressive on top of the standard of care immunosuppressive regimen. Furthermore, anti-infective prophylaxis therapy and viral testing has been implemented to mitigate the theoretical elevated infection risk (summarized in Section 4.6).

4.2. Treatment Arms and Duration

Participants will receive a single IV injection of GSK1070806 administered prior to kidney allograft reperfusion, and will be followed for 12 months post dose.

4.3. Type and Number of Subjects

- Adult subjects (18-75 years of age).
- Dialysis-dependent, recipient of first-time, single kidney-only, Donation after Circulatory Death (DCD) transplants, receiving a planned immunosuppressive standard of care comprised of basiliximab, mycophenolate mofetil (MMF) or azathioprine, tacrolimus and corticosteroids.
- For details of the number of subjects refer to go/no go decision criteria defined in Section 9. The number of subjects to be enrolled will be up to approximately 40.
- Since subjects will only receive a single dose of study medication, and patients normally undergo frequent clinic visits during the 12 months post transplantation, minimal attrition/withdrawal is anticipated for this study. However, if subjects prematurely discontinue the study replacements may be enrolled at the discretion of the Sponsor.

4.4. Design Justification

The single-arm, open-label design with a Bayesian sequential approach has been chosen in order to provide an early indication of clinical efficacy using a minimal sample size. The historical rate of DGF in the 7 days post DCD transplantation (a clinically meaningful parameter) was documented at approximately 50% during the time period 2005-2010 [Summers, 2013], and has been confirmed with contemporary registry data (2010-present) held by United Kingdom National Health Service Blood and Transplant (NHSBT). This background DGF rate forms the basis of the Bayesian 'go/no go' design.

The 12 month follow up period post transplantation, and administration of the single dose of GSK1070806, provides sufficient coverage to assess safety and tolerability once GSK1070806 levels decline and IL18 signalling is allowed to return. Furthermore, renal outcomes at 12 months post-transplant are deemed clinically relevant measures.

4.5. Dose Justification

<u>Preclinical Safety</u>: GSK1070806 is a monoclonal antibody with high affinity for human and rhesus/ cynomolgus IL-18 that does not interfere with interactions between IL-18 and the IL-18 binding protein. A 4-week repeat IV bolus GLP toxicology study in male and female cynomolgus monkey at 3, 30 and 300 mg/kg/week dose levels was conducted to assess doses for clinical use. No safety pharmacology findings of concern for clinical use were identified at any of these doses. Based on this, the NOAEL was defined as ≥300 mg/kg/week. The gender averaged exposure at 300 mg/kg/week dose was AUC0-168hr of 1775 mg*h/mL and Cmax of 18.8 mg/mL.

Clinical Experience: A single ascending dose study (A18110040) has been completed in healthy volunteers with GSK1070806 doses up to 10 mg/kg IV. Another study (A18116378) completed in obese subjects with type 2 diabetes mellitus (T2DM) evaluated repeat dosing at 2 dose levels (0.25 and 5 mg/kg IV). In this T2DM study two doses of GSK1070806 were administered to each cohort 4 weeks apart. Overall, all doses up to 10 mg/kg were well tolerated. The geometric mean half-life ($t_{1/2}$) ranged from 19 to 49 days, with a trend of dose-dependent increase in half-life. Based on available data, doses ≥ 1 mg/kg provide near complete suppression of systemic IL-18. No reported adverse events were associated with GSK1070806 administration in these studies.

GSK1070806 Dose Modelling for Renal Transplant: A physiological based pharmacokinetic (PBPK) model and prior clinical PK data were used to model the dose requirement based on the anticipated need to inhibit IL-18 (>90% target engagement) rapidly (1-2 hours post-dose) in both the interstitial space of the kidney and the circulating plasma. Using preliminary data on circulating IL-18 levels in renal transplant patients (anticipated maximum of 1400 pg/mL) and conservative assumptions that enhanced IL-18 levels in DGF were driven by increases in IL-18 production (versus slower metabolism) and derived within the kidney, target engagement with various doses of GSK1070806 was modelled. The PBPK model parameters were adjusted to test sensitivity with respect to extent of target engagement at various dose levels. Based on these data, in the current study we propose to use a single dose of 3 mg/kg administered prior to reperfusion of the transplanted kidney. The use of the single 3 mg/kg dose is well supported by the observed safety data generated in prior clinical studies (n=36 subjects have received ≥ 3 mg/kg) and the PBPK model based approach outlined above, which predicts a rapid engagement in the kidney interstitium followed by >90% inhibition for the 7 days leading up to assessment of the DGF primary endpoint.

Dose Adjustment: Data will be reviewed at an interim analysis after recruitment of 8 to 10 subjects [Section 9.3.2]. Depending on an integrated review of safety/tolerability, efficacy, and exposure, a dose adjustment will be considered. This review will include available data from the assessment of renal interstitial target engagement performed on renal biopsies, in which an estimated target engagement of less than approximately 90% in the majority of subjects will support consideration to increase dose above 3 mg/kg in subsequent recruited subjects. A minimum of 30 days post-dose safety experience in at least 6 subjects, along with a minimum of 7 days post-dose safety experience in the remaining interim subjects will be evaluated. Any decision to increase dose will be conducted in consultation with the GSK safety review team (SRT). The maximum dose level administered to patients will not exceed 10mg/kg as precedented by previous clinical studies.

4.6. Benefit:Risk Assessment

Summaries of findings from both clinical and non-clinical studies conducted with GSK1070806 can be found in the Investigators' Brochure. The following section outlines the risk assessment and mitigation strategy for this protocol:

4.6.1. Risk Assessment

Potential Risk of Clinical Significance	Summary of Data/Rationale for Risk	Mitigation Strategy
**Investigational Product (IP) [GSK1070806]**		
Infections	• IL-18 plays a role in host defense against microbial pathogens. IL-18 primes both innate and acquired immunity to viruses and other intracellular pathogens. As a result, there is a theoretical risk that blocking of IL-18 signalling by GSK1070806 may increase a patient's susceptibility to bacterial and viral infections.<br>• In the FTIH study no additional infections were observed after a single IV GSK1070806 infusion in 57 healthy and obese subjects when compared to placebo. Similarly, in a type 2 diabetes study, there was no apparent increase in infection rate in either treatment group (total of 25 treated subjects) when compared to placebo.<br>• Of note, patients in the current study are pharmacologically immunosuppressed as standard of care. Furthermore, IL-18 system neutralization is likely to have a duration of approximately 6-9 months at the 3mg/kg dose. Accordingly, infection is the key risk in this clinical program.	• **Dose selection**<br>The dose of 3 mg/kg has been selected as it is considered to be the minimum dose that will achieve sufficient IL18 inhibition in the initial hours after transplantation, during which it is presumed that the pathological processes causing DGF are initiated. This minimizes the duration of prolonged IL18 inhibition in order to mitigate any potential infection risk due to the addition of GSK1070806 to the immunosuppressive standard of care.<br>• **Eligibility criteria**<br>Patients will be excluded if they are at particular risk for infectious complications. Specifically, patients who are HIV+, have evidence of acute or chronic hepatitis B infection, are hepatitis C+, have a history of TB exposure or infection, are being actively managed for ongoing infection, or have a history of recent viral illness will be excluded. EBV-ve recipients of EBV+ve transplants will be excluded due to elevated risk of post-transplant lymphoproliferative disorder (PTLD). Transplant recipients receiving allografts from donors with significant bacterial, fungal or viral infections at the time of death will be excluded.<br>Patients for whom 'intensive' immunosuppressive regimes are planned including Alemtuzumab (Campath) or anti-thymocyte globulin, Belatacept, OKT3 or mTOR inhibitors- (known to increase risk of CMV, PTLD, and other infections) are excluded.

Potential Risk of Clinical Significance	Summary of Data/Rationale for Risk	Mitigation Strategy
		• **Standard of care**<br>Due to immunosuppressive treatments being standard of care patients routinely receive prophylactic intravenous antibiotics during transplantation, as well as prophylaxis for pneumocystis (PCP; usually co-trimoxazole, variable by centre), fungal infections (ie. nystatin), and cytomegalovirus (valganciclovir). CMV donor - /recipient - patients will receive leukodepleted blood and blood products (if needed), to minimise the risk of primary infection (per British Transplantation Society Guidelines, 2011).<br>Subjects will be monitored for symptoms and signs of infection including fever, hypotension, tachycardia and respiratory status. If necessary, cultures will be taken, and appropriate antibacterial, antifungal or antiviral therapy will be initiated. After discharge from hospital, subjects will be followed at regular intervals, being evaluated by professionals experienced in care of immunosuppressed organ transplant recipients.<br>• **Additions to standard of care**<br>In addition to routine infectious prophylaxis/ surveillance as described above, an elevated level of infectious prophylaxis/ surveillance will be mandated as follows:<br>– PCP prophylaxis will be continued for 12 months (as opposed to 3-6 months).<br>– CMV prophylaxis using valganciclovir for 200 days (as opposed to 100 days in some centres) will be provided as follows:<br>▪ Donor + /Recipient - (prevention of primary

Potential Risk of Clinical Significance	Summary of Data/Rationale for Risk	Mitigation Strategy
		infection)<br>▪ Donor + /Recipient + (prevention of re-infection)<br>▪ Donor - / Recipient + (prevention of re-activation)<br>▪ CMV PCR will be performed regularly after discontinuation of prophylaxis or 'for cause' at any time after transplantation in accordance with UK guidelines.<br>▪ Donor - /Recipient – will receive prophylaxis with acyclovir for 200 days, dose determined based on unit protocol.<br>– BK virus surveillance by BK PCR of whole blood will be performed post transplantation. In the event that a patient becomes BK +ve, screening will be repeated in accordance with UK guidelines. Immunosuppression will be reduced / titrated at the discretion of the investigator.<br>In the event of deteriorating renal function in the context of BK viremia, transplant biopsy to diagnose BK nephropathy will be considered at the discretion of the investigator.
Blood Pressure Effects	• In the T2DM study, hypertension was one of the most frequent AEs reported. Three patients in the placebo group (n=12) had mild hypertensive events (max systolic 182 mm/Hg, max diastolic 106 mm/Hg); five patients in the 0.25mg/kg group (n=13) had mild hypertensive events (max	• In the peri-operative and immediate post-operative period, renal transplant patients are closely monitored within an operating theatre / critical care environment. Settings are well equipped to respond to cardiovascular emergencies, as standard of care.

Potential Risk of Clinical Significance	Summary of Data/Rationale for Risk	Mitigation Strategy
	systolic 174 mm/Hg, max diastolic 112 mm/Hg, and in the 5mg/kg group (n=12), four patients had mild hypertensive events (max systolic 176mm/Hg). Detailed safety review indicated that the above values did not constitute an overall trend. However, blood pressure was flagged as a safety endpoint to monitor in subsequent studies.	• Throughout the hospital stay, blood pressure will be closely monitored, and anti-hypertensive medications titrated as standard of care. Thereafter, close outpatient monitoring, performed routinely in this population, will be used to identify and treat any blood pressure complications.
Immunogenicity	• A host immune response (immunogenicity) against GSK1070806 following IV administration is possible. Risk of immunogenicity is deemed to be low.<br>• The emergence of anti-GSK1070806 antibodies was investigated in the FTIH and Type 2 diabetes studies. To date, three subjects (of a total of 82 administered GSK1070806) have had confirmed relatively low titre of anti-GSK1070806 antibodies post-dosing. In 2 out 3 subjects the presence of anti-drug antibodies was transient, and is therefore not considered to be of clinical significance.<br>• To date there was no apparent evidence that anti-drug antibodies affected the pharmacokinetic profile of GSK1070806.<br>• Delayed-type, non-acute hypersensitivity reactions have not been observed with GSK1070806.	• The immunogenicity risk for GSK1070806 is deemed low. Nonetheless it is mitigated by use of a single dose in this study along with concomitant steroid treatment as SOC.<br>• Patients will be observed for symptoms such as rash, nausea, fatigue, myalgia, headache, and facial oedema.
Infusion reactions	• Risk of infusion reaction is judged to be low in view of the clinical experience with GSK1070806:<br>– In the FTIH study, no allergic reactions or infusion related reactions were observed after a single IV GSK1070806 infusion in 57 healthy and obese subjects.<br>– Additionally, no allergic or infusion related reactions	• Investigators/site personnel will be made aware of the risk of hypersensitivity reactions, which may present as an infusion reaction, and will monitor patients closely for a minimum of 3 hours.<br>• Investigational product will be administered by the investigator/site personnel prepared to manage infusion reactions and anaphylaxis, following their standard practices to

Potential Risk of Clinical Significance	Summary of Data/Rationale for Risk	Mitigation Strategy
	were observed in T2DM study in which 25 subjects were administered GSK1070806.	manage any untoward infusion reactions noted during or after the infusion period.<br>• Subjects will be on corticosteroids and hospitalized during dosing.<br>• For subjects who have previously received IV immunoglobulin (IVIG) or subjects with a history of allergies (allergic responses to food, drugs, insects, or a history of urticaria), diphenhydramine (12.5 to 50 mg based on clinical judgment) or equivalent and paracetamol (acetaminophen) may be administered prophylactically prior to dosing. Antihistamine H2-receptor antagonists (e.g., ranitidine) are also permitted.
**Study Procedures**		
Renal biopsy	• Renal biopsy may cause bleeding / injury to the transplanted organ.	• Intra-operative biopsy will be performed under direct visualization thus any bleeding will be controlled immediately by the operating surgeon. Biopsies are optional per protocol. Therefore if a surgeon judges that risk of biopsy is unacceptable, they will have the option not to perform this procedure.

4.6.2. Benefit Assessment

Delayed graft function is an area of considerable unmet need, affecting up to half of renal transplant recipients, and contributing to worse long term transplantation outcomes. To date no approved therapies are available for DGF, or broader acute kidney injury indications. It is anticipated that GSK1070806 will decrease the incidence of DGF and thereby improve long term renal function, resulting in lower rates of graft failure. In addition, other mechanisms, particularly antagonism of Th1 mediated rejection, may support a beneficial effect of IL-18 inhibition and contribute to an enhancement of 1 year graft survival.

4.6.3. Overall Benefit/Risk Conclusion

Weighing the existing preclinical and clinical safety record of GSK1070806 with the potential for benefit in terms of prevention of DGF and reduction in risk of allograft rejection, it is judged that the potential risks are justified.

5. SELECTION OF STUDY POPULATION AND WITHDRAWAL CRITERIA

Specific information regarding warnings, precautions, contraindications, adverse events, and other pertinent information on the GSK investigational product or other study treatment that may impact subject eligibility is provided in the Investigators Brochure.

Deviations from inclusion and exclusion criteria are not allowed because they can potentially jeopardize the scientific integrity of the study, regulatory acceptability or subject safety. Therefore, adherence to the criteria as specified in the protocol is essential.

5.1. Inclusion Criteria

A subject will be eligible for inclusion in this study only if all of the following criteria apply:

AGE
1. **Recipient age range**: Between 18 and 75 years of age inclusive, at the time of signing the informed consent.

TYPE OF SUBJECT AND DIAGNOSIS INCLUDING DISEASE SEVERITY
**2. Dialysis-dependent recipient of first time, <u>single kidney-only</u>, Donation after Circulatory Death (DCD) transplant.**
3. **Eligible for kidney transplantation**: Considered eligible for transplantation after undergoing multidisciplinary evaluation at the institution at which the transplantation will be performed.
4. **Immunosuppressants (at the time of transplantation):** planned to receive a combination of immunosuppressants including basiliximab, mycophenolate mofetil or azathioprine, tacrolimus, and corticosteroids.

SEX
5. **Male and Female**:<br>**Males:**<br>Male subjects with female partners of child bearing potential must utilize a condom and female partners must comply with use of one of the highly effective contraceptive methods described in Appendix 4 for 180 days post-dose of study medication.<br>**Females**:<br>a. Non-reproductive potential defined as:<br>• Females with one of the following procedures documented and no plans to utilize assisted reproductive techniques (e.g., in vitro fertilization or donor embryo transfer):<br>– Bilateral tubal ligation or salpingectomy<br>– Hysteroscopic tubal occlusion procedure with follow-up confirmation of bilateral tubal occlusion<br>– Hysterectomy<br>– Bilateral Oophorectomy (surgical menopause)<br>• Postmenopausal defined as 12 months of spontaneous amenorrhea.<br>– In questionable cases (including cases in which amenorrhea is suspected to result from a subject's poor renal function/dialysis) a blood sample with simultaneous follicle stimulating hormone (FSH) and estradiol levels consistent with menopause are required. If FSH/estradiol results are not available in time subjects are to be initiated on contraceptive methods (see below and Appendix 4).<br>– Females on hormone replacement therapy (HRT) and whose menopausal status is in doubt will be required to use one of the highly effective contraception methods if they wish to continue their HRT during the study.<br>b. Reproductive potential and agrees to follow one of the options listed in the Modified List of Highly Effective Methods for Avoiding Pregnancy in Females of Reproductive Potential (FRP) (see Appendix 4) for 180 days post dose. If a hormonal method of birth control is selected from the list in Appendix 4 then subjects must have been using these methods at least 28 days prior to GSK1070806 administration, or be abstinent, or utilize a condom as a method of contraception until the selected hormonal method has been in place for the 28 day period.<br>**The investigator is responsible for ensuring that subjects understand how to properly use the indicated methods of contraception by providing counsel directly or by referring subjects to health care professionals with expertise in this area.**

INFORMED CONSENT
6. **Capable of providing signed informed consent** as described in Section 10.2 which includes compliance with the requirements and restrictions listed in the consent form and in this protocol.

5.2. Exclusion Criteria

A subject will not be eligible for inclusion in this study if any of the following criteria apply:

CONCURRENT CONDITIONS/MEDICAL HISTORY (INCLUDES LIVER FUNCTION AND QTc INTERVAL)
1. **Liver function**: ALT >2xULN and bilirubin >1.5xULN (isolated bilirubin >1.5xULN is acceptable if bilirubin is fractionated and direct bilirubin <35%).
2. **QT interval**: single or average QTc > 480 msec or in subjects with bundle branch block QTc > 500 msec (these criteria do not apply to subjects with predominately paced rhythms).

CONCOMITANT MEDICATIONS
3. **Concurrent medication**: Subjects who receive treatment that is prohibited for safety reasons (e.g. live vaccines, cyclophosphamide or other biologic immunosuppressants as described in Section 6.11) should not receive investigational product without the explicit approval of the Medical Monitor (Sponsor).
4. **Investigational product**: Any within 5 half-lives or twice the duration of the biological effect whichever is longer (investigational product refers to any drug not approved for sale in the country in which it is being used).
5. **Immunosuppression**: Are being considered for steroid-free, anti-thymocyte globulin (ATG) or alemtuzumab induction, which have a much more profound and prolonged immunosuppressive effect than basiliximab.
6. **Prior biologic immunosuppressives**: The subject has received an agent within the following time period prior to the day of dosing in the current study: 30 days, 5 half-lives or twice the duration of the biological effect, whichever is longer.
7. **Vaccines**: A live vaccine within 30 days prior to GSK1070806 administration.

CONTRAINDICATIONS
**8. Receiving a DCD kidney allograft from a donor with any of the following characteristics**
a. cold ischemic time > 36 hours
b. age < 5 years old
c. age >75 years old.

d. ABO blood type incompatible against the recipient.

e. T- and/or B-cell positive crossmatch by complement dependent cytotoxicity or flow cytometry against the recipient (where positive crossmatch is unavailable, virtual crossmatch is allowed).

f. serology positive for hepatitis B (except hepatitis B surface antibody and prior vaccination), hepatitis C or human immunodeficiency virus (HIV)

g. EBV positive donor allograft with an EBV negative recipient

h. donor had acute or chronic bacterial, viral or fungal infection that according to the investigator causes a risk to recipient, particularly if the infection was resistant or systemic

i. normothermic regional machine perfusion organ retrieval techniques were utilized

9. **Previous organ transplantation**: has previously undergone any other organ transplantation (with the exception of corneal transplantation).

10. **Malignancy:** has a history of malignancy in the past 5 years except for adequately treated cancers of the skin (basal or squamous cell) or carcinoma in situ of the uterine cervix.

11. **Acute or chronic infection:** has required management of acute or chronic infections (excludes prophylaxis of infections), as follows:

    a. currently being treated for a chronic infection, which in the opinion of the investigator, could put the subject at undue risk

    b. hospitalized for treatment of infection, or treated for an infection with parenteral antibiotics (includes antibacterials, antivirals, anti-fungals, or anti-parasitic agents) within 30 days before Day 0, which in the opinion of the investigator, could put the subject at undue risk

    c. current evidence, or history within the last 14 days, of an influenza-like illness as defined by fever (>38°C) and two or more of the following symptoms: cough, sore throat, runny nose, sneezing, limb / joint pain, headache, vomiting / diarrhoea

    d. patients with any history of active tuberculosis, recent tuberculosis exposure, or judged by investigators to be at risk of tuberculosis will be excluded from the study

12. **Other disease/conditions.** Has any of the following:

    a. clinical evidence of significant unstable or uncontrolled acute or chronic diseases, which in the opinion of the investigator, could confound the results of the study or put the subject at undue risk

    b. a surgical procedure planned in the 12 months after Day 0, other than kidney transplantation or related procedure

    c. a known history of any other medical disease (e.g., cardiopulmonary), laboratory abnormality, or condition (e.g., poor venous access) that, in the

opinion of the investigator, makes the subject unsuitable for the study

13. **Hepatitis B**: patients will be excluded with **any** evidence of acute or chronic infection, or if interpretation of their results is unclear. This includes:
    a. HBsAg +
    b. Anti-HBc +
    c. HB DNA+

    It is permissible to enrol patients who are anti-HBs+ **only**, when this is attributable to vaccination and there is **no** history of previous infection.
14. **Hepatitis C**: patients will be excluded if there is any evidence of past or current hepatitis C infection, including hepatitis C antibody, hepatitis C RIBA immunoblot or PCR.
15. **HIV:** known to have a historically positive HIV test.
16. **Immunodeficiency:** recipient with a history of, or laboratory evidence of immunodeficiency.
17. **Drug Sensitivity:** has a history of sensitivity to any of the study medications including:
    a. GSK1070806
    b. background immunosuppressive regimen,
    c. designated prophylactic anti-infective therapies

    or components thereof, or a history of drug or other allergy including a previous anaphylactic reaction to parenteral administration or biologic therapy (ie monoclonal antibody) that, in the opinion of the Investigator or Medical Monitor, contraindicates their participation.
18. **Substance abuse:** has clinical evidence of current drug or alcohol abuse or dependence.
19. **Co enrolment:** participating in another interventional study (participation in purely observational or cohort studies is acceptable provided they do not impair feasibility, or involve excessive additional sampling).
20. **Compliance:** is unlikely to comply with scheduled study visits based on investigator judgment or has a history of a psychiatric disorder or condition that may compromise communication with the investigator.

5.3. Screening/Baseline/Run-in Failures

Screen failures are defined as subjects who consent to participate in the clinical trial but do not receive study medication. In order to ensure transparent reporting of screen failure subjects, meet the Consolidated Standards of Reporting Trials (CONSORT) publishing requirements, and respond to queries from Regulatory authorities, a minimal set of screen failure information is required including Demography, Screen Failure details, Eligibility Criteria, and Serious Adverse Events (see Section 7.4.1.5).

5.4. Withdrawal/Stopping Criteria

A subject may withdraw from study participation at any time at his/her own request, or may be withdrawn at any time at the discretion of the investigator for safety, behavioural or administrative reasons. Since participants will receive a single dose of study medication on day 0, once this dose has been given, withdrawal will constitute declining to participate in follow-up activities. If a subject withdraws from the study, he/she may request destruction of any samples taken, and the investigator must document this in the site study records.

If a subject withdraws from the study, they should be strongly encouraged to return for subsequent visits, particularly the Safety Follow-up visit at 12 months, and to continue with adjustments to infectious prophylaxis/ standard of care medicines intended to mitigate safety risks associated with administration of a long-acting, novel immunosuppressive.

The following actions must be taken in relation to a subject who fails to attend the clinic for a required study visit:

- The site must attempt to contact the subject and re-schedule the missed visit as soon as possible.
- The site must counsel the subject on the importance of maintaining the assigned visit schedule.
- The site must counsel the subject on the importance of maintaining additional anti-infectious prophylaxis and testing regimens.
- In cases where the subject is deemed 'lost to follow up', the investigator or designee must make every effort to regain contact with the subject (where possible, 3 telephone calls and if necessary a certified letter to the subject's last known mailing address or local equivalent methods). These contact attempts should be documented in the subject's medical record.
- Should the subject continue to be unreachable, only then will he/she be considered to have withdrawn from the study with a primary reason of "Lost to Follow-up".

5.4.1. Liver Chemistry Management

Since participants will receive a single dose of study medication, there are no liver-specific stopping criteria as such. In the event of liver parameters deviating from the thresholds described in Appendix 2, follow instructions in that section.

Liver chemistry increased monitoring criteria have been designed to assure subject safety and evaluate liver event etiology (in alignment with the FDA premarketing clinical liver safety guidance).
http://www.fda.gov/downloads/Drugs/GuidanceComplianceRegulatoryInformation/Guidances/UCM174090.pdf

5.4.2. QTc Evaluation Criteria

Further monitoring and/or evaluation should be considered in any subject who meets the following:

- QTc >530 msec OR uncorrected QT >600 msec (machine or manual overread)

For subjects with underlying **bundle-branch block**, follow the evaluation criteria listed below:

Baseline QTc with Bundle-Branch Block	Criteria for Further Evaluation and Monitoring of QTc with Bundle-Branch Block
<480 msec	>530 msec
480 to 500 msec	≥550 msec

- The enhanced monitoring criteria are based on an average QTc value of triplicate ECGs. If an ECG demonstrates a prolonged QT interval, obtain 2 more ECGs over a brief period, and then use the averaged QTc values of the 3 ECGs to determine whether the subject meets these criteria.
- The *same* QT correction formula *must* be used for *all QTc data being collected for each individual subject* to determine eligibility for the study and to determine whether further evaluation and/or monitoring is/are necessary. This formula may not be changed or substituted once the subject has been enrolled. (Safety ECGs and other non-protocol-specified ECGs are an exception.)
  - For example, if a subject is eligible for the protocol based on QTcB, then QTcB must be used for instigation of enhanced monitoring criteria of this individual subject as well.
- Considerations as actions for further monitoring and evaluation should include the following, as appropriate:
  - Assessment of any relevant symptoms (e.g., presyncope);
  - Follow-up ECGs to assess for resolution of QTc prolongation;
  - Review of concomitant or recently taken medications, including over the counter preparations and herbal supplements
  - Evaluation of electrolytes to include magnesium and potassium
  - Referral to an emergency facility/cardiology consultation for further evaluation and monitoring, or any other evaluation that may be necessary as determined by the investigator.

5.5. Safety Interim Analysis

A safety interim assessment after 8-10 subjects will be performed at the same time as the dose adjustment interim (Section 4.5). If the rate/severity of infectious SAEs exceeds that expected on background immunosuppression based on the SRT medical judgement (SRT

will include an independent transplant infectious disease specialist) then recruitment may be paused. The expected background rate of infection will be determined based on data from publications, registries, trial site databases, and ongoing studies in comparable renal transplant populations.

5.6. Subject and Study Completion

A completed subject will be defined as a patient who receives the scheduled study infusion and completes the 12 month assessment.

The end of the study is defined as the last subject's last clinic visit.

6. STUDY TREATMENT

6.1. Investigational Product and Other Study Treatment

The term 'study treatment' is used throughout the protocol to describe any combination of products received by the subject as per the protocol design. Study treatment may therefore refer to the individual study treatments or the combination of those study treatments.

**Product name:**	GSK1070806
**Dosage form:**	100 mg/mL injectable solution
**Unit dose strength(s)/Dosage level(s):**	Single dose of 3 mg/kg (maximum of 10 mg/kg if dose adjustment deemed necessary, based on interim analysis - Section 4.5)
**Route of Administration**	IV infusion prior to allograft reperfusion
**Dosing instructions:**	Dilute into 100 mL sterile IV infusion bag 0.9% Sodium Chloride (details see Study Reference Manual)

The rate of infusion should not be increased above the recommended rate, but may be slowed or interrupted if the subject appears to develop signs of adverse reactions or infusion-related symptoms. See SRM for IV infusion rate details.

6.2. Treatment Assignment

This is a non-randomized, single-arm study and all subjects will receive active investigational product.

6.3. Blinding

This will be a single-arm unblinded study.

6.4. Packaging and Labeling

The contents of the label will be in accordance with all applicable regulatory requirements.

6.5. Preparation/Handling/Storage/Accountability

A description of the methods and materials required for preparation of GSK1070806 is detailed in the Study Reference Manual (SRM).

Study treatment must be dispensed or administered according to procedures described herein and in the SRM. Only subjects enrolled in the study may receive study treatment. Only authorized site staff may supply or administer study treatment. All study treatment must be stored in a secure area with access limited to the investigator and authorized site staff.

The investigator, institution, or the head of the medical institution (where applicable) is responsible for study treatment accountability, reconciliation, and record maintenance. The investigator or the head of the medical institution (where applicable), or designated site staff (e.g., storage manager, where applicable) must maintain study treatment accountability records throughout the course of the study. The responsible person(s) will document the amount of study treatment received from and returned to GSK and the amount administered to subjects. The required accountability unit for this study will be the vial. Discrepancies are to be reconciled or resolved. Procedures for final disposition of unused study treatment are listed in the SRM.

Investigational product is not expected to pose significant occupational safety risk to site staff under normal conditions of use and administration. However, precautions are to be taken to avoid direct skin contact, eye contact, and generating aerosols or mists. In the case of unintentional occupational exposure notify the monitor, medical monitor and/or study manager. A Material Safety Data Sheet (MSDS)/equivalent document describing occupational hazards and recommended handling precautions either will be provided to the investigator, where this is required by local laws, or is available upon request from GSK.

6.6. Compliance with Study Treatment Administration

GSK1070806 will be administered intravenously to subjects as an infusion at the site; they will receive study treatment directly from the investigator or designee, under medical supervision. The date and time of each dose administered in the clinic will be recorded in the source documents. The dose of study treatment and study subject identification will be confirmed at the time of dosing by a member of the study site staff other than the person administering the study treatment.

6.7. Treatment of Study Treatment Overdose

For this study, any dose of GSK1070806 > 10 mg/kg will be considered an overdose.

There are no known antidotes and GSK does not recommend specific treatment for an overdose. In the event of an overdose the investigator will use clinical judgement in treating the symptoms and should:

1. Contact the Medical Monitor immediately.

2. Closely monitor the subject for adverse events (AEs)/serious adverse events (SAEs) and laboratory abnormalities per the investigator's discretion for at least 12 months or twice the length of the anticipated biologic effect of the dose received.
3. Obtain a plasma sample for pharmacokinetic (PK) analysis within 2 days from the date of the study treatment overdose if requested by the Medical Monitor (determined on a case-by-case basis)
4. Document the quantity of the excess dose in the CRF.

6.8. Treatment after the End of the Study

Subjects will not receive any additional treatment from GSK after completion of the study because all enrolled subjects are already receiving standard of care as background therapy. The investigator is responsible for ensuring that consideration has been given to the post-study care of the subject's medical condition.

6.9. Lifestyle and/or Dietary Restrictions

All subjects should follow the recommended lifestyle and dietary restrictions deemed as standard of care by investigators for renal transplant patients.

6.10. Concomitant Medications and Non-Drug Therapies

GSK1070806 will be administered in addition to the defined immunosuppressant regimen (Section 6.10.1). Concomitant medication adjustments will be made as clinically indicated and at the investigator's discretion. All medication that is necessary for the appropriate clinical management of subjects in this study will be allowed with the exception of those listed in Section 6.11.

6.10.1. Standard Immunosuppressive Therapy

Patients should be intended to receive a combination immunosuppression comprised of basiliximab; mycophenolate mofetil (MMF) or aziothioprine; tacrolimus; and corticosteroids. The immunosuppressant regimen may be revised based on the clinical judgment of the investigator including titration of tacrolimus target trough levels. Caution is advised if target tacrolimus levels are anticipated to exceed 10μg/L. Dose adjustments of immunosuppressant agents should be recorded.

6.10.2. Management of low white cell count

If patients are found to have white cell count $\leq 4 \times 10^9$/l then the investigator should consider proactively reducing dose of valgancyclovir or MMF per site specific clinical practice. In the event that valgancyclovir prophylaxis is discontinued, CMV PCR should be performed at a frequency according to the risk perceived by the investigator. If MMF dosage is reduced, investigators should be vigilant for increased risk of rejection.

6.10.3. Graft Dysfunction/Graft rejection

Investigations for the cause of graft dysfunction/rejection and subsequent treatment will follow institutional protocol. If additional immunosuppressive medications are administered for a diagnosed/suspected rejection episode it is suggested that the investigator consider prolonging prophylaxis/surveillance for infection (Section 6.10.4).

6.10.4. Prophylaxis and Surveillance for Infection

Investigators may consult with the medical monitor in regards to determining individual prophylaxis treatment and surveillance plans in order to mitigate any increased risk of infection. Investigators should take into consideration that an additional immunosuppressive, GSK1070806, has been added to treatment, and therefore intensive prophylaxis and surveillance against perioperative bacterial, pneumocystis, viral and fungal infections is appropriate.

6.10.4.1. Pneumocystis and Fungal Infection Prophylaxis

A minimum of 12 months of pneuomocystis and fungal infection prophylaxis with cotrimoxazole and nystatin, respectively (or equivalents), should be implemented unless otherwise contraindicated. In cases of cotrimoxazole intolerance, then alternative therapy may be considered at the discretion of the investigator and in discussion with the medical monitor.

6.10.4.2. Cytomegalovirus Infection

Subjects at risk of CMV disease include:

- Donor +ve /Recipient -ve (risk of primary infection)
- Donor +ve /Recipient +ve (risk of re-infection)
- Donor -ve /Recipient +ve (risk of re-activation)

CMV infection prophylaxis with a 200 day course of valganciclovir will be administered to all subjects at risk of CMV disease as listed above. If the recipient is prescribed additional immunosuppression for allograft rejection during the course of prophylaxis, the course will be extended such that the patient receives treatment for 200 days afterwards; if anti-rejection medicines are administered at between six and twelve months post-transplantation, a repeat 200 day course of valganciclovir will be prescribed.

After completion of prophylaxis, subjects will undergo routine surveillance for CMV viraemia by polymerase chain reaction testing of whole blood in accordance with UK guidelines. In addition, ‘for cause’ CMV testing may be performed at any time during the first post-transplant year at the discretion of the investigator. In cases of low white cell counts, valganciclovir dose can be reduced or discontinued at the investigator’s discretion in consultation with the medical monitor. If valganciclovir is discontinued, CMV PCR should be performed at a frequency appropriate to the perceived risk of CMV infection.

Subjects that are CMV:

- Donor -ve /Recipient –ve

Should receive prophylaxis with acyclovir for 200 days, with dose determined based on unit protocol.

6.10.4.3. BK virus

To address a theoretical increased risk of BK virus infection, subjects will undergo surveillance by BK PCR of whole blood per UK guidelines, and as outlined in Section 7.

In the event that a patient becomes BK +ve, screening should be repeated at subsequent study visits and according to local practice. Immunosuppression will be reduced / titrated at the discretion of the investigator. In the event of deteriorating renal function in the context of BK viremia a transplant biopsy, to diagnose BK nephropathy, should also be considered at the discretion of the investigator.

6.11. Prohibited Medications and Non-Drug Therapies

6.11.1. Vaccines

Live vaccines are not permitted 30 days prior to GSK1070806 administration, or during the study period.

6.11.2. Concomitant Medications

Subjects who start prohibited medications or therapies at any time during the study should be discussed with the Medical Monitor (Sponsor). Since a single dose of GSK1070806 is administered at day 0 in this study subjects should continue with study assessments and be closely followed up for safety.

The following medications and therapies are prohibited at any time during the study:

- Use of other investigational agents (biologic or non-biologic, or non-drug therapy; investigational applies to any drug not approved for sale in the country in which it is used).
- Use of other immunosuppressive agents (e.g., alemtuzumab, belatacept, rituximab, eculizumab, ATG, OKT3, mTOR inhibitors) is discouraged, although it is recognized that use of these therapies may be unavoidable in certain clinical situations such as steroid resistant rejection. In these circumstances, the medical monitor should be consulted, and investigators should extend infectious prophylaxis and surveillance as described above.

7. STUDY ASSESSMENTS AND PROCEDURES

This section lists the procedures and parameters of each planned study assessment. The exact timing of each assessment is listed in the Time and Events Table Section 7.1

Protocol waivers or exemptions are not allowed with the exception of immediate safety concerns. Therefore, adherence to the study design requirements, including those specified in the Time and Events Table, are essential and required for study conduct.

The following points must be noted:

- If assessments are scheduled for the same nominal time, then assessments should occur in the following order:
    1. 12-lead ECG
    2. vital signs
    3. blood draws

  Note: The timing of the assessments should allow the blood draw to occur at the defined time point.
- The timing and number of planned study assessments, including safety, pharmacokinetic, pharmacodynamic/biomarker assessments may be altered during the course of the study based on newly available data to ensure appropriate monitoring.
- The change in timing or addition of time points for any planned study assessments must be documented in a 'Note to File' which is approved by the relevant GSK study team member and then archived in the study sponsor and site study files, but this will not constitute a protocol amendment.
- The IRB/IEC will be informed of any safety issues that require alteration of the safety monitoring scheme or amendment of the Informed Consent Form.
- Approximately 250-300 mL of blood will be collected over the first 30 days of this study, for study-related procedures.

7.1. Time and Events Table

Study Day[1]	Screening	Pre-operative (baseline)	Intra-operative (D0) [1]	4-8h [2]	D1	D2	Daily until discharge	Discharge	D30	D90	6 & 12 months [13]	Unscheduled Visit
Informed Consent/Demographics	X											
Medical/drug/alcohol/tobacco history	X											
GSK1070806 IV Infusion		←------------------------------→[15]										
Kidney preservation fluid IL-18			X[16]									
Kidney biopsy (post-reperfusion) [3]			X (~45 min)									
Clinical biopsy reports (if applicable)		←--→										
Concomitant medication	X	X	X[17]	X	X	X	X	X	X	X	X	X
Evidence of infection	X			X	X	X	X	X	X	X	X	X
SAE / AE monitoring	←--→											
DGF status (up to day 7)				←------------------------------→								
Urine output		X (native output)	X	X	X	X	X	X				
Dialysis events since last assessment		X		X	X	X	X	X	X	X	X	X
Graft survival			←--→									
Viral serology (recipient & donor – HIV, Hep B/C, EBV) [4]	X[5]	X									X	X
Viral serology (recipient & donor - herpes, varicella) [4]		X										
CMV monitoring	X[5]	X									X	X
BK virus monitoring [6]		X							X	X	X	X
Vital signs [7]	X			X	X	X	X	X	X	X	X	X
Complete physical	X										X	X
12-lead ECG	X							X			X	X
Hematology / Clinical Chemistry / Urinalysis	X			X	X	X	X	X	X	X	X	X
Pregnancy test [8]	X								X	X	X	X
Serum cytokines / PD biomarkers		X[11]	X (~45 min at biopsy)	X	X	X		X	X	X	X	X
Free, GSK1070806 bound & total IL18 [9]		X[11]	X (~45 min at biopsy)	X	X	X		X	X	X	X	X
Pharmacokinetics [9,10]		X[11]	(~45 min at biopsy)	X	X			X (@168 hrs or at earlier discharge)	X	X	X	X

**Study Day**[1]	**Screening**	**Pre-operative (baseline)**	**Intra-operative (D0)** [1]	**4-8h** [2]	**D1**	**D2**	**Daily until discharge**	**Discharge**	**D30**	**D90**	**6 & 12 months** [13]	**Unscheduled Visit**
Immunogenicity [12]		X[11]							X	X	X	X
Urinary biomarkers		X[11]		X	X	X	X	X	X	X	X	X
Peripheral blood leucocyte flow phenotyping [14]		X						X		X	X	
PBMCs frozen for in vitro stimulation assays [14]		X						X		X	X	
PBMCs placed in trizol for transcriptomic analysis [14]		X						X		X	X	

1. Day 0 defined as the calendar day of transplant.
2. Time post reperfusion.
3. Biopsies during transplantation may be 'core or wedge' at the discretion of the operating surgeon. If, in the judgement of the operating surgeon, biopsy poses unwarranted risk to the patient, it may be omitted.
4. Viral serology will include HIV, HBsAg, anti-HBc, anti-HBs antibodies, and hepatitis C antibody (if hepatitis C antibody positive, a hepatitis C RIBA immunoblot or polymerase chain reaction assay should be reflexively performed on the same sample to confirm the results as per NHS standard procedure), and serology for herpes simplex virus, Epstein Barr virus, and varicella zoster virus.
5. Screening viral status may be the most recent value recorded in the patient's medical records.
6. If BK positive then follow up screening should be implemented.
7. Vital signs will include systolic and diastolic blood pressure, pulse rate, and temperature.
8. Pregnancy testing will be performed in women of childbearing potential. Screening test may be urine or serum.
9. Sampling time points may be adjusted as appropriate based on emerging data.
10. PK sampling time points are as follows: predose, 45 min to 1 hr into infusion (coinciding with biopsy), 4-8 hrs, 24 hrs, 168 hrs (or pre-discharge if discharge before day 7), day 30 day 90, 6 and 12 months. Exact PK sampling time is to be recorded.
11. Blood and urine samples for immunogenicity and biomarker analyses must be collected prior to infusion with GSK1070806
12. For any non-completer a serum sample for immunogenicity testing will be taken 12 mths post-operatively.
13. For subjects who withdraw from study, where possible, assessments should continue according to the Time and Events table. The 6 and 12 mth safety follow-up should be performed.
14. **Samples for subjects from Cambridge site only.**
15. GSK1070806 can be infused any time during the pre-operative and/or intra-operative periods, but infusion must be complete prior to reperfusion of the allograft.
16. Sample to be obtained at time of back-table allograft preparation.
17. Documentation of anaesthetics excluded.

7.2. Screening and Critical Baseline Assessments

Cardiovascular medical history/risk factors (as detailed in the CRF) will be assessed at screening.

The following demographic parameters will be captured: year of birth, sex, race and ethnicity.

Medical/medication/family history, including infections, as well as drug/alcohol/tobacco history will be assessed as related to the inclusion/exclusion criteria listed in Section 5.

Procedures conducted as part of the subject's routine clinical management and obtained prior to signing of informed consent may be utilized for Screening or baseline purposes provided the procedure meets the protocol-defined criteria.

7.3. Efficacy

The incidence of DGF is the primary efficacy measure. Investigations for the cause of graft dysfunction / rejection will follow institutional protocol. Acknowledging the subjective component of a DGF diagnosis, a secondary analysis of serum creatinine data and dialysis events will be performed.

In the event that a subject develops graft dysfunction, the assessments described in Section 7 'unscheduled visit' will be obtained by the investigator/site staff and their results recorded in the eCRF for inclusion in study analyses.

In order to realise the greatest clinical benefit, a drug reducing incidence of DGF would also prolong graft survival. To this end, in addition to short term renal DGF outcome (up to day 7), the following will be captured:

a. Graft survival up to 1 year.
b. 30 & 90 day, and 6 & 12 month serum creatinine.
c. Urinary biomarkers indicative of injury, including NGAL, KIM-1, IL-18.
d. Clinical, biopsy proven acute rejection episodes.
e. Biomarkers indicative of risk of allograft rejection, including IP-10, Mig, inflammatory cytokines.

7.4. Safety

Planned time points for all safety assessments are listed in the Time and Events Table (Section 7.1). Additional time points for safety tests such as vital signs, physical exams and laboratory safety tests may be added during the course of the study based on newly available data to ensure appropriate safety monitoring.

7.4.1. Adverse Events (AE) and Serious Adverse Events (SAEs)

The definitions of an AE or SAE can be found in Section 12.3.

The investigator and their designees are responsible for detecting, documenting and reporting events that meet the definition of an AE or SAE.

7.4.1.1. Time period and Frequency for collecting AE and SAE information

- Any SAEs assessed as related to study participation (e.g., protocol-mandated procedures, invasive tests, or change in existing therapy) or related to a GSK product will be recorded from the time a subject consents to participate in the study up to and including any follow-up contact.
- AEs will be collected from the start of Study Treatment until the follow-up contact (see Section 7.4.1.3), at the time points specified in the Time and Events Table (Section 7.1).
- Medical occurrences that begin prior to the start of study treatment but after obtaining informed consent may be recorded on the Medical History/Current Medical Conditions section of the CRF.
- All SAEs will be recorded and reported to GSK within 24 hours, as indicated in Section 12.3.
- Investigators are not obligated to actively seek AEs or SAEs in former study subjects. However, if the investigator learns of any SAE, including a death, at any time after a subject has been discharged from the study, and he/she considers the event reasonably related to the study treatment or study participation, the investigator must promptly notify GSK.

NOTE: The method of recording, evaluating and assessing causality of AEs and SAEs plus procedures for completing and transmitting SAE reports to GSK are provided in Appendix 4.

7.4.1.2. Method of Detecting AEs and SAEs

Care will be taken not to introduce bias when detecting AEs and/or SAEs. Open-ended and non-leading verbal questioning of the subject is the preferred method to inquire about AE occurrence. Appropriate questions include:

- “How are you feeling?”
- “Have you had any (other) medical problems since your last visit/contact?”
- “Have you taken any new medicines, other than those provided in this study, since your last visit/contact?”

If such questioning is not possible, the investigator will use his/her medical judgment in determining whether an event is considered to be an AE or SAE.

7.4.1.3. Follow-up of AEs and SAEs

After the initial AE/SAE report, the investigator is required to proactively follow each subject at subsequent visits/contacts. All SAEs, and non-serious AEs of special interest (as defined in Section 4.6.1) will be followed until resolution, until the condition stabilizes, until the event is otherwise explained, or until the subject is lost to follow-up

(as defined in Section 5.4). Further information on follow-up procedures is given in Appendix 3.

7.4.1.4. Cardiovascular and Death Events

For any cardiovascular events detailed in Section 12.3 and all deaths, whether or not they are considered SAEs, specific Cardiovascular (CV) and Death sections of the CRF will be required to be completed. These sections include questions regarding cardiovascular (including sudden cardiac death) and non-cardiovascular death.

The CV CRFs are presented as queries in response to reporting of certain CV MedDRA terms. The CV information should be recorded in the specific cardiovascular section of the CRF within one week of receipt of a CV Event data query prompting its completion.

The Death CRF is provided immediately after the occurrence or outcome of death is reported. Initial and follow-up reports regarding death must be completed within one week of when the death is reported.

7.4.1.5. Regulatory Reporting Requirements for SAEs

Prompt notification by the investigator to GSK of SAEs related to study treatment (even for non- interventional post-marketing studies) is essential so that legal obligations and ethical responsibilities towards the safety of subjects and the safety of a product under clinical investigation are met.

GSK has a legal responsibility to notify both the local regulatory authority and other regulatory agencies about the safety of a product under clinical investigation. GSK will comply with country specific regulatory requirements relating to safety reporting to the regulatory authority, Institutional Review Board (IRB)/Independent Ethics Committee (IEC) and investigators.

Investigator safety reports are prepared for suspected unexpected serious adverse reactions according to local regulatory requirements and GSK policy and are forwarded to investigators as necessary.

An investigator who receives an investigator safety report describing a SAE(s) or other specific safety information (e.g., summary or listing of SAEs) from GSK will file it with the IB and will notify the IRB/IEC, if appropriate according to local requirements.

7.4.2. Pregnancy

- Details of all pregnancies in female subjects and female partners of male subjects will be collected after the start of dosing and until 180 days post dose.
- If a pregnancy is reported then the investigator should inform GSK within 2 weeks of learning of the pregnancy and should follow the procedures outlined in Appendix 4.

7.4.3. Physical Exams

- A complete physical examination will include, at a minimum, assessment of the Cardiovascular, Respiratory, Gastrointestinal and Neurological systems. Height and weight will also be measured and recorded.
- Investigators should pay special attention to clinical signs related to previous serious illnesses.

7.4.4. Vital Signs

- Vital sign measurements will include systolic and diastolic blood pressure, pulse rate and temperature.
- Vital signs will be measured in semi-supine position after 5 minutes rest.

7.4.5. Electrocardiogram (ECG)

- Single or triplicate 12-lead ECGs will be obtained at each time point indicated in TE table (Section 7.1) using an ECG machine that automatically calculates the heart rate and measures PR, QRS, QT, and QTc intervals. Refer to Section 5.4.2 for when triplicate QTc readings are required, and for QTc enhanced monitoring criteria.

7.4.6. Clinical Safety Laboratory Assessments

All protocol required laboratory assessments, as defined in Table 1 below, must be conducted in accordance with the Laboratory Manual, and Protocol Time and Events Schedule. Laboratory requisition forms must be completed and samples must be clearly labelled with the subject number, protocol number, site/centre number, and visit date. Details for the preparation and shipment of samples will be provided by the laboratory and are detailed in the SRM. Reference ranges for all safety parameters will be provided to the site by the laboratory responsible for the assessments.

If additional non-protocol specified laboratory assessments are performed at the institution's local laboratory and result in a change in subject management or are considered clinically significant by the investigator (e.g., SAE or AE or dose modification) the results must be recorded in the CRF.

Refer to the SRM for appropriate processing and handling of samples to avoid duplicate and/or additional blood draws.

Haematology, clinical chemistry, urinalysis and additional parameters to be tested are listed in Table 1.

Table 1 Protocol Required Safety Laboratory Assessments

Laboratory Assessments	Parameters			
Haematology	Platelet Count	*RBC Indices*:	*WBC count with Differential*:	
	RBC Count	MCV	Neutrophils	
	Hemoglobin	MCH	Lymphocytes	
	Hematocrit		Monocytes	
			Eosinophils	
			Basophils	
Clinical Chemistry [1]	Urea	Potassium	GGT	Bilirubin
	Creatinine	Sodium	ALT	Total protein
	Glucose	Calcium	Alkaline phosphatase	Albumin
Routine Urinalysis	• Specific gravity<br>• pH, glucose, protein, blood and ketones by dipstick<br>• Microscopic examination (if blood or protein is abnormal)			
Other Tests	• HIV, CMV, BK, Herpes, Varicella, EBV<br>• Hepatitis B (HBsAg, anti-HBc, anti-HBs)<br>• Hepatitis C (Hep C antibody)<br>• As needed in women of non-child bearing potential only:<br>– FSH and estradiol<br>• For women of child bearing potential only:<br>– hCG pregnancy test, blood (urine only at screening)			

NOTES :

1. Details of Liver Chemistry Increased Monitoring Criteria and Required Actions and Follow-Up Assessments after an event is reached are given in Section 5.4.1 and Appendix 2

All laboratory tests with values that are considered clinically significantly abnormal during participation in the study should be repeated until the values return to normal or baseline. If such values do not return to normal within a period judged reasonable by the investigator, the etiology should be identified and the sponsor notified.

7.4.7. Immunogenicity

Low titres of ADAs have been observed post dosing with GSK1070806, but only one patient (of 82 exposed) had levels which were detectable on more than one occasion.

Serum samples for testing antibodies against GSK1070806 will be collected as described in the Time and Events table (Section 7.1). Final samples will be taken when serum levels of GSK1070806 are lowest, and are anticipated to be below that causing ADA assay interference. For any non-completer, an attempt will be made to obtain a sample at 30 days or later post dose.

The presence of anti-GSK1070806 binding antibodies will be assessed using a validated electrochemiluminescent (ECL) immunoassay. Immunogenicity sample testing will be performed by Clinical Immunology, GlaxoSmithKline. Details of blood sample collection (including volume to be collected), processing, storage and shipping procedures are provided in the SRM. For each subject, immunogenicity results, including the incidence and titers, will be reported.

7.5. Pharmacokinetics

7.5.1. Blood Sample Collection and Analysis

Blood samples for pharmacokinetic (PK) analysis of GSK1070806 will be collected at the time points indicated in Section 7.1, Time and Events Table. The actual date and time of each blood sample collection will be recorded. The timing of PK samples may be altered and/or PK samples may be obtained at additional time points to ensure thorough PK monitoring.

Processing, storage and shipping procedures are provided in the Study Reference Manual (SRM).

Serum analysis will be performed under the control of PTS-DMPK/Scinovo, GlaxoSmithKline, the details of which will be included in the Study Reference Manual (SRM). Concentrations of GSK1070806 will be determined in serum samples using the currently approved bioanalytical methodology. Raw data will be archived at the bioanalytical site (detailed in the SRM).

Once the serum has been analyzed for GSK1070806 any remaining serum may be analyzed for other drug-related catabolites and the results reported under a separate PTS-DMPK/Scinovo, GlaxoSmithKline protocol.

7.5.2. Renal Graft Biopsies

Graft biopsies will be obtained approximately 45 minutes post reperfusion of the kidney prior to closure of the abdominal wall. Post-reperfusion biopsies will be sent to GSK and utilized to assess pharmacokinetics/pharmacodynamics by detection of IL-18 and GSK1070806 within the renal parenchyma. Evaluating both IL-18 and GSK1070806 levels within the interstitium of the kidney during early reperfusion is likely to be helpful in understanding the level of target engagement.

Intra-operative biopsies may be ‘core’ or ‘wedge’ at the discretion of the operating surgeon. If the operating surgeon deems obtaining the biopsy as a potential risk to the subject this procedure may be omitted at their discretion. Processing, storage and shipping procedures are provided in the Study Reference Manual (SRM).

7.6. Mechanistic Biomarker(s)/Pharmacodynamic Markers

Ischemia reperfusion has a direct negative impact in the kidney causing hypoxic cellular injury, ATP depletion, membrane depolarization, and free radical toxicity (Kalogeris et al., 2012). These cellular insults induce cytokine release and cellular infiltration to the site of injury. IL-18 is a primary driver of the innate immune response leading to acute renal injury.

7.6.1. Cytokines

IL-18 was first described as an IFNγ–inducing factor in the circulation of endotoxin-injected mice (Nakamura et al., 1989). In this study we will follow the effects of GSK1070806 on IL-18 signalling following transplant, by taking measures of primary and secondary IL-18 responsive cytokines at time points defined in TE table (Section 7.1).

7.6.2. Renal Graft Biopsies

Biopsies will be obtained as outlined in Section 7.5.2 and assessed by histology and immunohistochemistry. If sufficient tissue is available, the sample will also be examined for immune cell infiltrates. Significant influx of innate immune cells has been observed on biopsies taken from renal allografts within the first hour of reperfusion, and the burden of infiltrating cells has been correlated with graft outcome [Fuggle 1993], with more prominent infiltrates in kidneys from cadaveric donor derived allografts than living donors [Koo 1998]. IL-18 has pleiotropic effects upon multiple cytokine systems and immune cells. It is therefore reasonable to hypothesize that IL-18 antagonism will decrease cellular infiltrates following ischemic renal injury.

Where other clinical biopsy reports are available the data will be captured in the eCRF.

7.6.3. Biomarkers relating to acute rejection

The IFN-γ -inducible chemokines IP10 (CXCL10) and Mig (CXCL9) have been identified as early predictive markers of antibody-mediated kidney graft rejection [Hancock, 2001; Hauser, 2005; Matz, 2006]. This likely reflects their function in recruiting effector T cells to the site of inflammation. We therefore will monitor changes in serum biomarkers, including IP10 and Mig, as IL-18 dependent predictors of allograft rejection.

7.6.4. Clinical Biomarkers

7.6.4.1. Urinary biomarkers of injury

<u>NGAL:</u> One of the earliest urinary markers of injury to appear is NGAL (Neutrophil-gelatinase-associated lipocalin) which is transcriptionally induced rapidly following ischemia and secreted from intrarenal cells. Elevated levels of NGAL protein are detectable in the urine within 3 hours after injury and its production is sustained for up to 5 days [Mishra, 2003].

KIM1: KIM1 is expressed on T cells and is shed from the membrane in a matrix metalloprotein-dependent fashion following ischemia or toxic injury [Han, 2002]. It is also considered to be an early marker of AKI. However its peak occurrence in the urine within 48 hours reflects a comparatively delayed response to injury consistent with its proposed role in renal recovery [Fontanilla, 2011].

IL-18 is itself rapidly secreted from intracellular stores following inflammasome mediated-activation [Keyel, 2014]. Elevated levels of urinary IL-18 appear within 6 hours after injury and peak at 12-18 hours. The appearance of IL-18 marks the initiation of the inflammatory response leading to further injury [Wu, 2008]. We will monitor urinary NGAL and KIM1, and IL-18 levels starting from time 0 (preoperative) and over time post-transplantation as outlined in TE table Section 7.1.

Other disease urinary biomarkers may be assessed, as relevant, to enhance understanding of the IL-18 mechanism of action. All samples will be retained for a maximum of 5 years after the last subject completes the trial.

7.6.4.2. Renal preservation fluid sampling

Emerging data indicate that higher IL-18 levels in kidney preservation fluid may predict delayed graft function and poorer outcome of transplantation. This fluid will be sampled and analysed for IL-18. Results will be compared with clinical outcomes.

7.6.5. Circulating cellular phenotype (Cambridge only)

IL-18 receptor is expressed on macrophages, lymphocytes, neutrophils, and natural killer cells. Evidence from published studies suggests that IL-18 influences diverse immune functions and regulates other important mediators of immune responses. To understand the cellular immune response to transplantation in the absence of IL-18 signalling, serial whole blood samples will be examined by flow cytometry, RNA transcriptome analysis and in *in vitro* stimulation assays.

8. DATA MANAGEMENT

- For this study subject data will be entered into GSK defined CRFs, transmitted electronically to GSK or designee and combined with data provided from other sources in a validated data system.
- Management of clinical data will be performed in accordance with applicable GSK standards and data cleaning procedures to ensure the integrity of the data, e.g., removing errors and inconsistencies in the data.
- Adverse events and concomitant medications terms will be coded using MedDRA (Medical Dictionary for Regulatory Activities) and an internal validated medication dictionary, GSKDrug.
- CRFs (including queries and audit trails) will be retained by GSK, and copies will be sent to the investigator to maintain as the investigator copy. Subject initials will not be collected or transmitted to GSK according to GSK policy.

9. STATISTICAL CONSIDERATIONS AND DATA ANALYSES

9.1. Hypotheses

The primary objective is to determine the effect of GSK1070806 on DGF. Stopping rules have been defined to test whether the DGF rate is less than or equal to the null hypothesis rate of 50%.

A Bayesian sequential analysis of efficacy data will be utilized to allow for the possibility of stopping early for success or failure. Study success/failure will be declared once substantial evidence is observed. "Success" at full enrolment is defined as 11 or fewer DGF events in 30 patients. After each patient has been evaluated during the course of the study, early success on the primary endpoint may be declared if the predictive probability of success is >0.92 (i.e. the probability the eventual number of DGF events would be 11 or fewer if the study were to continue to full enrolment) and a failure may be declared on the primary endpoint if the predictive probability of success is <0.02. This check is made sequentially as each patient becomes evaluable by reaching 7 days post transplant. The coloured grid in Figure 3 illustrates which events may trigger an early Go (success) decision by green as the predictive probability of success >0.92 and No Go (failure) decision by red as the predictive probability of success < 0.02.

9.2. Sample Size Considerations

9.2.1. Sample Size Assumptions

The DGF rate has been established at approximately 50% based on literature [Summers, 2013] and contemporary registry data held by NHS Blood and Transplant.

Success in this study with full enrollment would be no more than 11 DGF occurrences in 30 evaluable subjects. A Beta(5,8) prior distribution is set on the rate of DGF. This prior was selected because the Bayes estimate has smaller mean squared error than the maximum likelihood estimate over the true range of DGF rates from 20% to 60% for all sample sizes less than or equal to 30.

As shown in Figure 2, at a maximum of 30 subjects, this design yields the probability of a 'go' decision of 0.139 when the GSK1070806 DGF rate is 50% (ie: null hypothesis) and 0.69 at what has been considered to be a clinically impactful GSK1070806 DGF rate of 35%.

Figure 2 Operating Characteristic Curve

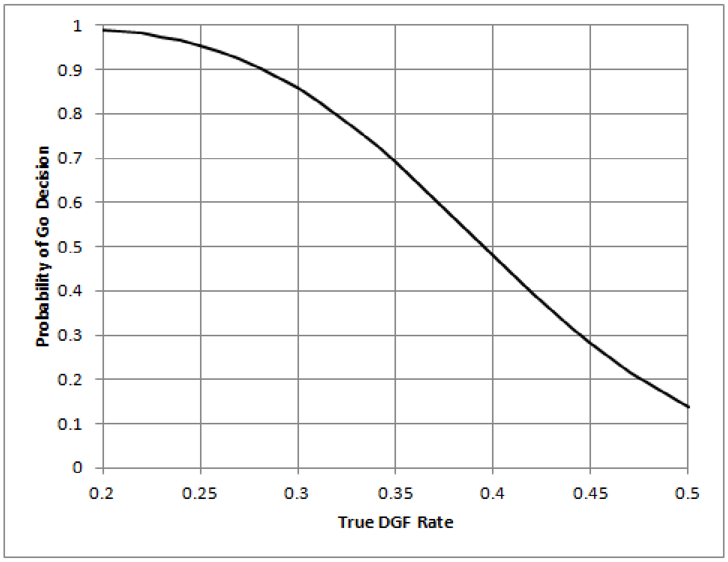

Up to approximately 30 subjects may be enrolled. Efficacy data generated during the study will be used to inform the probability that GSK1070806 is performing at the above-mentioned response rates. This study may therefore conclude prior to completion of enrolling 30 subjects. Under the null hypothesis (i.e. DGF rate = 50%) the probability that a no-go decision is declared by 50% enrolment (15 patients) is 0.3271 and by 75% enrolment (22 patients) is 0.6033.

9.2.2. Sample Size Sensitivity

Sample sizes of 25, 30, 35, 40, and 45 were explored. Table 2 shows the type I error rates for a DGF rate of 50% and power for detecting a 35% DGF rate for the various sample size assumptions. The type I error rate is the probability of a go decision if the true DGF rate is 50% (i.e., the null) and the power is the probability of a go decision if the true DGF rate is 35%. Increasing the sample size above 30 subjects has diminishing returns on type I error and power for the study.

Table 2 Probability of Go Decision under the Null for Various Sample Sizes

	Probability of Go Decision	
	Type I error (if true DGF rate is 50%)	Power (if true DGF rate is 35%)
n_{max} = 25	0.143	0.659
n_{max} = 30	0.139	0.690
n_{max} = 35	0.135	0.716
n_{max} = 40	0.138	0.742
n_{max} = 45	0.114	0.747

9.2.3. Sample Size Re-estimation or Adjustment

No sample size re-estimation will be performed. The analysis of the primary endpoint is sequential and will be performed adaptively.

9.3. Data Analysis Considerations

9.3.1. Analysis Populations

All Subjects Population

The 'All Subjects Population' is defined as all subjects who receive a dose of study medication.

Analysis Population

For DGF related endpoints the 'Analysis Population' (AP) is defined as subjects in the 'All Subjects' population who have been declared to have DGF or have reached 7 days. For other endpoints, the AP is defined as subjects having baseline and at least one post-baseline assessment.

Per Protocol Population (PP)

The 'Per Protocol Population' will consist of any AP subjects who are compliant with protocol-specific criteria. Subjects with specified protocol deviations will be excluded. The Per Protocol population will be used for analysis of DGF rates.

Pharmacokinetic (PK) Population

The 'PK Population' is defined as subjects in the 'All Subjects' population for whom a serum pharmacokinetic sample was obtained and analyzed for GSK1070806.

9.3.2. Interim Analysis

Safety, tolerability and efficacy data will be reviewed by the GSK study team on an ongoing basis throughout the study. These reviews can include individual subject data, summaries by treatment group and graphical displays. Preliminary safety, efficacy and PK results may be reported prior to database freeze for the purposes of safety review by GSK and where required by regulatory bodies.

The sequential decision rule is shown graphically in Figure 3. The number in the first column indicates the number of subjects who have completed study treatment. From the point in time when approximately 6 subjects have reached day 7 post transplantation the sponsor study team will begin to evaluate the DGF rates. This will start with the first 6 subjects and then with addition of data from each subsequent subject that completes 7 days of the trial. A sequential Go/No Go/Continue rule is based on the predictive probability of success. A high predictive probability (PP) of success means that GSK1070806 is likely to be efficacious by the end of the study given the observed data, whereas a low PP suggests that the treatment may not have sufficient activity. If the PP value is less than 2% (red region) the trial may be stopped and the alternative hypothesis will be rejected. If the PP is greater 92% (green), the conclusion may be made that GSK1070806 has better efficacy than the standard of care. If the PP is between 2 - 92% (white region), the trial will continue to the next interim analysis (to be conducted after the next subject has completed treatment) or until reaching 30 completed subjects.

This decision chart serves as guidance for the decision making at interim data reviews in terms of the primary efficacy endpoint. However, all efficacy and safety data will also be reviewed at the same time. Decisions will be made based on the totality of the data.

In addition to sequential review of the DGF data as noted above, an interim analysis will be performed after recruitment of approximately 8 to 10 subjects to review for a consideration of dose adjustment based on an integrated review of safety/tolerability, efficacy, and exposure [Section 4.5]. Any decision to change dose will be conducted in consultation with the GSK safety review team. If a dose adjustment is made, then the decision grid in Figure 3 would return to the top and up to a maximum of 30 subjects at the adjusted dose would be evaluated sequentially using the same Go/No Go/Continue rules as defined above. Hence approximately 40 subjects would be recruited into the current study if the dose is adjusted.

Figure 3 Sequential Decision Rules

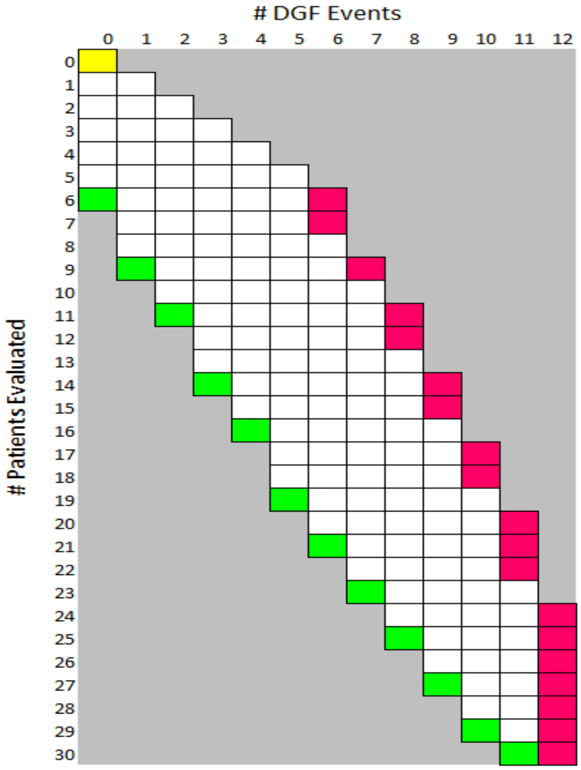

9.4. Key Elements of Analysis Plan

Complete details will be documented in the Reporting and Analysis Plan (RAP). Any deviations from, or additions to, the original analysis plan described in this protocol will be documented in the RAP and final study report.

Final analyses will be performed after all subjects have completed or withdrawn from the study and after database freeze. Version 9.1 or higher of the SAS system will be used to analyze the data as well as to generate tables, figures, and listings.

For continuous data, descriptive statistics of percentage change from baseline, change from baseline data and raw data where indicated in the RAP, (n, arithmetic, standard deviation, minimum, median and maximum) will be calculated and summarized by time. Graphical displays will be produced by time if deemed appropriate. For binary data, number of events and percentages will be calculated. Subject listings will be produced.

9.4.1. Primary Analyses

A summary of the observed DGF rate during the study will be provided. The posterior probability that the proportion of subjects experiencing DGF is less than 50% will be calculated.

9.4.2. Secondary Analyses

Summaries of baseline and post-dose values or proportions of subjects at various time points will be provided. Graft survival will be summarized by Kaplan-Meier plots.

9.4.3. Other Analyses

Exploratory analyses will be summarized as noted above.

9.4.4. Safety Analyses

Safety data will be presented in tabular and/or graphical format, and will be summarized descriptively according to GSK's Integrated Data Standards Library (IDSL) standards.

9.4.5. Pharmacokinetic Analyses

Pharmacokinetic sampling will be related to the start of the GSK1070806 infusion procedure.

PK analysis will be the responsibility of the GSK Clinical Pharmacology, Modelling and Simulation (CPMS) Department. Key PK parameters such as AUC may be calculated subject to data availability. PK parameters, if any generated, will be presented using appropriate graphic and tabular summaries. Systemic GSK1070806 concentrations obtained from this study may be analyzed in a population PK analysis, to characterize further any potential effects of disease status, demographic or other patient characteristics based on data availability. The analysis details will be included in the Reporting and Analysis Plan (RAP).

Data permitting serum and urinary total and free IL-18 levels will be determined as outlined in the Time and Events table (Section 7.1).

10. STUDY GOVERNANCE CONSIDERATIONS

10.1. Posting of Information on Publicly Available Clinical Trial Registers

Study information from this protocol will be posted on publicly available clinical trial registers before enrollment of subjects begins.

10.2. Regulatory and Ethical Considerations, Including the Informed Consent Process

Prior to initiation of a site, GSK will obtain favourable opinion/approval from the appropriate regulatory agency to conduct the study in accordance with ICH Good Clinical Practice (GCP) and applicable country-specific regulatory requirements.

The study will be conducted in accordance with all applicable regulatory requirements, and with GSK policy.

The study will also be conducted in accordance with ICH Good Clinical Practice (GCP), all applicable subject privacy requirements, and the guiding principles of the current version of the Declaration of Helsinki. This includes, but is not limited to, the following:

- IRB/IEC review and favorable opinion/approval of the study protocol and amendments as applicable
- Obtaining signed informed consent
- Investigator reporting requirements (e.g. reporting of AEs/SAEs/protocol deviations to IRB/IEC)
- GSK will provide full details of the above procedures, either verbally, in writing, or both.
- Signed informed consent must be obtained for each subject prior to participation in the study.
- The IEC/IRB, and where applicable the regulatory authority, approve the clinical protocol and all optional assessments, including genetic research.
- Optional assessments (including those in a separate protocol and/or under separate informed consent) and the clinical protocol should be concurrently submitted for approval unless regulation requires separate submission.
- Approval of the optional assessments may occur after approval is granted for the clinical protocol where required by regulatory authorities. In this situation, written approval of the clinical protocol should state that approval of optional assessments is being deferred and the study, with the exception of the optional assessments, can be initiated.

10.3. Quality Control (Study Monitoring)

- In accordance with applicable regulations including GCP, and GSK procedures, GSK monitors will contact the site prior to the start of the study to review with the site staff the protocol, study requirements, and their responsibilities to satisfy regulatory, ethical, and GSK requirements.
- When reviewing data collection procedures, the discussion will also include identification, agreement and documentation of data items for which the CRF will serve as the source document.

GSK will monitor the study and site activity to verify that the:

- Data are authentic, accurate, and complete.
- Safety and rights of subjects are being protected.
- Study is conducted in accordance with the currently approved protocol and any other study agreements, GCP, and all applicable regulatory requirements.

The investigator and the head of the medical institution (where applicable) agrees to allow the monitor direct access to all relevant documents

10.4. Quality Assurance

- To ensure compliance with GCP and all applicable regulatory requirements, GSK may conduct a quality assurance assessment and/or audit of the site records, and the regulatory agencies may conduct a regulatory inspection at any time during or after completion of the study.
- In the event of an assessment, audit or inspection, the investigator (and institution) must agree to grant the advisor(s), auditor(s) and inspector(s) direct access to all relevant documents and to allocate their time and the time of their staff to discuss the conduct of the study, any findings/relevant issues and to implement any corrective and/or preventative actions to address any findings/issues identified.

10.5. Study and Site Closure

- Upon completion or premature discontinuation of the study, the GSK monitor will conduct site closure activities with the investigator or site staff, as appropriate, in accordance with applicable regulations including GCP, and GSK Standard Operating Procedures.
- GSK reserves the right to temporarily suspend or prematurely discontinue this study at any time for reasons including, but not limited to, safety or ethical issues or severe non-compliance. For multicenter studies, this can occur at one or more or at all sites.
- If GSK determines such action is needed, GSK will discuss the reasons for taking such action with the investigator or the head of the medical institution (where applicable). When feasible, GSK will provide advance notification to the investigator or the head of the medical institution, where applicable, of the impending action.
- If the study is suspended or prematurely discontinued for safety reasons, GSK will promptly inform all investigators, heads of the medical institutions (where applicable) and/or institution(s) conducting the study. GSK will also promptly inform the relevant regulatory authorities of the suspension or premature discontinuation of the study and the reason(s) for the action.
- If required by applicable regulations, the investigator or the head of the medical institution (where applicable) must inform the IRB/IEC promptly and provide the reason for the suspension or premature discontinuation.

10.6. Records Retention

- Following closure of the study, the investigator or the head of the medical institution (where applicable) must maintain all site study records (except for those required by local regulations to be maintained elsewhere), in a safe and secure location.
- The records must be maintained to allow easy and timely retrieval, when needed (e.g., for a GSK audit or regulatory inspection) and must be available for review in conjunction with assessment of the facility, supporting systems, and relevant site staff.

- Where permitted by local laws/regulations or institutional policy, some or all of these records can be maintained in a format other than hard copy (e.g., microfiche, scanned, electronic); however, caution needs to be exercised before such action is taken.
- The investigator must ensure that all reproductions are legible and are a true and accurate copy of the original and meet accessibility and retrieval standards, including re-generating a hard copy, if required. Furthermore, the investigator must ensure there is an acceptable back-up of these reproductions and that an acceptable quality control process exists for making these reproductions.
- GSK will inform the investigator of the time period for retaining these records to comply with all applicable regulatory requirements. The minimum retention time will meet the strictest standard applicable to that site for the study, as dictated by any institutional requirements or local laws or regulations, GSK standards/procedures, and/or institutional requirements.
- The investigator must notify GSK of any changes in the archival arrangements, including, but not limited to, archival at an off-site facility or transfer of ownership of the records in the event the investigator is no longer associated with the site.

10.7. Provision of Study Results to Investigators, Posting of Information on Publically Available Clinical Trials Registers and Publication

Where required by applicable regulatory requirements, an investigator signatory will be identified for the approval of the clinical study report. The investigator will be provided reasonable access to statistical tables, figures, and relevant reports and will have the opportunity to review the complete study results at a GSK site or other mutually-agreeable location.

GSK will also provide the investigator with the full summary of the study results. The investigator is encouraged to share the summary results with the study subjects, as appropriate.

The procedures and timing for public disclosure of the results summary and for development of a manuscript for publication will be in accordance with GSK Policy.

11. REFERENCES

Agostini C et al. (2001). Cxcr3 and its ligand CXCL10 are expressed by inflammatory cells infiltrating lung allografts and mediate chemotaxis of T cells at sites of rejection. *Am.J.Pathol*. 158, 1703-1711.

Alge JL & Arthur JM (2015). Biomarkers of AKI: A Review of Mechanistic Relevance and Potential Therapeutic Implications. *Clin.J.Am.Soc.Nephrol*. 10, 147-155.

Allograft rejection. Transpl Int. 20, 460-6.

British Transplantation Society Guidelines, 2011 https://www.bts.org.uk/Documents/Guidelines/Active/Final%20CMV%20Guideline%20for%20web%2012-9-2011.pdf

Coca (2014). Urinary Biomarkers of AKI and Mortality 3 Years after Cardiac Surgery. J Am Soc Nephrol 25, 1063–1071.

Cruzado J et al. (2000). “Glomerulonephritis associated with hepatitis C virus infection after renal transplantation,” Nephrology Dialysis Transplantation, vol. 15, supplement 8, pp. 65–67.

Daemen MA, Denecker G, van't Veer C, Wolfs TG, Vandenabeele P, Buurman WA. Activated caspase-1 is not a central mediator of inflammation in the course of ischemia-reperfusion. Transplantation. 2001 Mar 27;71(6):778-84

Dudler J et al. (2007). Gene transfer of interleukin-18-binding protein attenuates cardiac

Fabrizi F et al. (2004). “HBV infection in patients with end-stage renal disease,” Seminars in Liver Disease, vol. 24, no. 1, pp. 63–70.

Field M et al. (2014). The use of NGAL and IP-10 in the prediction of early acute rejection in highly sensitized patients following HLA-incompatible renal transplantation. Transpl Int. 27, 362-70.

Fontanilla J & Han WK (2011). Kidney injury molecule-1 as an early detection tool for acute kidney injury and other kidney diseases. Expert.Opin.Med.Diagn. 5, 161-173.

Fortin CF et al. (2009). Autocrine role of endogenous interleukin-18 on inflammatory cytokine generation by human neutrophils. FASEB J. 1, 194-203.

Fuggle SV et al. (1993). Variation in expression of endothelial adhesion molecules in pretransplant and transplanted kidneys--correlation with intragraft events. Transplantation. 55, 117-23

Futosi K et al. (2013). Neutrophil cell surface receptors and their intracellular signal transduction pathways. *Int.Immunopharmacol*. 17, 638-650.

Gracie JA et al. (2003). Interleukin-18. *J.Leukoc.Biol.* 73, 213-224.

Gu et al. (1997). Activation of interferon-gamma inducing factor mediated by interleukin-1beta converting enzyme. Science. 275, 206-9.

Hall et al. (2010). IL-18 and Urinary NGAL Predict Dialysis and Graft Recovery after Kidney Transplantation. *J Am Soc Nephrol* 21, 189–197.

Hall et al. (2012). Association between Peritransplant Kidney Injury Biomarkers and 1-Year Allograft Outcomes. *Clin J Am Soc Nephrol* 7, 1224–1233.

Han WK et al. (2002). Kidney Injury Molecule-1 (KIM-1): a novel biomarker for human renal proximal tubule injury. *Kidney Int.* 62, 237-244.

Hancock et al. (2001). Donor-derived IP-10 initiates development of acute allograft rejection. *J.Exp.Med*. 193, 975-980.

Hatcher RA et al. editors (2011). Contraceptive Technology. 20th edition. Atlanta, Georgia: Ardent Media, Inc., 50. Table 3-2.

Hauser IA et al. (2005). Prediction of acute renal allograft rejection by urinary monokine induced by IFN-gamma (MIG). *J.Am.Soc.Nephrol*. 16, 1849-1858.

He et al. (2008). Interleukin-18 binding protein transgenic mice are protected against ischemic acute kidney injury. *Am J Physiol Renal Physiol*. 295: F1414–F1421.

Issa F et al. (2010). Role of T cells in graft rejection and transplantation tolerance. Expert Rev Clin Immunol. 6, 155-69.

Kalia H et al. (2011). Hepatitis B virus and renal transplantation, Transplantation Reviews. 25, 102–109.

Kalogeris T et al (2012). Cell biology of ischemia/reperfusion injury. *Int.Rev.Cell Mol.Biol.* 298, 229-317.

Keyel, P.A. (2014). How is inflammation initiated? Individual influences of IL-1, IL-18 and HMGB1. *Cytokine* 69, 136-145.

Kimura K et al. (2002). Interleukin-18 Inhibits Hepatitis B Virus Replication in the Livers of Transgenic Mice *J. Virology* 76, 10702–10707.

Koo DD et al. (1998). Ischemia/reperfusion injury in human kidney transplantation: an immunohistochemical analysis of changes after reperfusion. Am J Pathol. 153, 557-66.

Kunikata T et al. (1998). Constitutive and induced IL-18 receptor expression by various peripheral blood cell subsets as determined by anti-hIL-18R monoclonal antibody. *Cell Immunol*. 189, 135-143.

Kwon O et al. (2003). Urinary actin, interleukin-6, and interleukin-8 may predict sustained ARF after ischemic injury in renal allografts. *Am.J.Kidney Dis.* 41, 1074-1087.

Lewington AJ, Cerdá J, Mehta RL. Raising awareness of acute kidney injury: a global perspective of a silent killer. Kidney Int. 2013 Sep;84(3):457-67

Mathurin P et al. (1999). Impact of hepatitis B and C virus on kidney transplantation outcome. Hepatology. 29, 257–263.

Matz M et al. (2006) Early post-transplant urinary IP-10 expression after kidney transplantation is predictive of short- and long-term graft function. *Kidney Int.* 69, 1683-1690.

Melnikov et al. (2001). Impaired IL-18 processing protects caspase-1–deficient mice from ischemic acute renal failure. *J Clin Invest.* 107, 1145–1152.

Melnikov et al. (2002). Neutrophil-independent mechanisms of caspase-1– and IL-18–mediated ischemic acute tubular necrosis in mice. J. Clin. Invest. 110, 1083–1091.

Mishra J et al. (2003). Identification of neutrophil gelatinase-associated lipocalin as a novel early urinary biomarker for ischemic renal injury. J.Am.Soc.Nephrol. 14, 2534-2543.

Mistry P et al. (2014). Safety, tolerability, pharmacokinetics, and pharmacodynamics of single-dose antiinterleukin- 18 mAb GSK1070806 in healthy and obese subjects. Int J Clin Pharmacol Ther. 52, 867-79.

Moers C et al. (2009). The influence of deceased donor age and old-for-old allocation on kidney transplant outcome. Transplantation. 88, 542-52.

Musso T et al. (1992). IL-2 induces IL-6 production in human monocytes. *J.Immunol.* 148, 795-800.

Nakamura K et al. (1989). Endotoxin-induced serum factor that stimulates gamma interferon production. Infect.Immun. 57, 590-595.

Okamura H et al. (1995). A novel costimulatory factor for gamma interferon induction found in the livers of mice causes endotoxic shock. Infect Immun. 63, 3966-72.

Parikh C et al. (2011). Postoperative Biomarkers Predict Acute Kidney Injury and Poor Outcomes after Adult Cardiac Surgery. *J Am Soc Nephrol* 22, 1748–1757.

Puren AJ et al. (1998). Interleukin-18 (IFNgamma-inducing factor) induces IL-8 and IL-1beta via TNFalpha production from non-CD14+ human blood mononuclear cells. *J.Clin.Invest* 101*,* 711-721.

Puren AJ et al. (1999). Gene expression, synthesis, and secretion of interleukin 18 and interleukin 1b are differentially regulated in human blood mononuclear cells and mouse spleen cells. PNAS 96, 2256–2261.

Rabant M et al. (2015). Urinary C-X-C Motif Chemokine 10 Independently Improves the Noninvasive Diagnosis of Antibody-Mediated Kidney Allograft Rejection. J Am Soc Nephrol. 26, 2840-51.

Rewa O & Bagshaw S (2014). Acute kidney injury—epidemiology, outcomes and economics. *Nat. Rev. Nephrol*. 10, 193–207

Ryden L et al. (2014). Acute Kidney Injury After Coronary Artery Bypass Grafting and Long-Term Risk of End-Stage Renal Disease. Circulation. 130, 2005-2011.

Sharma A et al. (2008). Elevation of interleukin-18 in chronic hepatitis C: implications for hepatitis C virus pathogenesis Immunology, 128, e514–e522.

Shigeoka AA, Mueller JL, Kambo A, Mathison JC, King AJ, Hall WF, Correia Jda S, Ulevitch RJ, Hoffman HM, McKay DB. An inflammasome-independent role for epithelial-expressed Nlrp3 in renal ischemia-reperfusion injury. J Immunol. 2010 Nov 15;185(10):6277-85

Striz I et al. (2005). Interleukin 18 (IL-18) upregulation in acute rejection of kidney allograft. Immunol Lett. 99, 30-5.

Summers D et al. (2013). Effect of donor age and cold storage time on outcome in recipients of kidneys donated after circulatory death in the UK: a cohort study. Lancet 381, 727–34.

Tak PP et al. (2006). Pharmacokinetics of IL-18 binding protein in healthy volunteers and subjects with rheumatoid arthritis or plaque psoriasis. Eur J Drug Metab Pharmacokinetics. 31, 109-16.

Tapiawala SN et al. (2010). Delayed graft function and the risk for death with a functioning graft. J Am Soc Nephrol. 21, 153-61.

Terrault N et al. (2007). The Kidney Transplant Recipient with Hepatitis C Infection: Pre- and Posttransplantation Treatment. Clin J Am Soc Nephrol 2, 563-575.

Tone et al. (1997). Regulation of IL-18 (Interferon-ϒ inducing factor) gene expression. J. Immunol. 159, 6156-63.

Tsutsui H et al. (1996). IFN-gamma-inducing factor up-regulates Fas ligand-mediated cytotoxic activity of murine natural killer cell clones. *J.Immunol*. 157, 3967-3973.

Wu H et al. (2008). IL-18 contributes to renal damage after ischemia-reperfusion. *J.Am.Soc.Nephrol*. 19, 2331-2341.

Yoshimoto T et al. (1998a). IL-12 up-regulates IL-18 receptor expression on T cells, Th1 cells, and B cells: synergism with IL-18 for IFN-gamma production. *J.Immunol*. 161, 3400-3407.

Yoshimoto, T et al. (1998b). IL-12 up-regulates IL-18 receptor expression on T cells, Th1 cells, and B cells: synergism with IL-18 for IFN-gamma production. J.Immunol. 161, 3400-3407.

Zhang et al. (2011). IL-18 mediates proapoptotic signaling in renal tubular cells through a Fas ligand-dependent mechanism. Am J Physiol Renal Physiol. 301, F171-8.

Zhang Q et al., (2014). Serum fractalkine and interferon-gamma inducible protein-10 concentrations are early detection markers for acute renal allograft rejection. Transplant Proc. 46, 1420-5.

12. APPENDICES

12.1. Appendix 1: Abbreviations and Trademarks

Abbreviations

AE	Adverse Event
GSK	GlaxoSmithKline

Trademark Information

Trademarks of the GlaxoSmithKline group of companies
ADVAIR

Trademarks not owned by the GlaxoSmithKline group of companies
Chiron RIBA
SAS
WinNonlin

12.2. Appendix 2: Liver Safety Required Actions and Follow up Assessments

Phase II liver chemistry increased monitoring criteria have been designed to assure subject safety and evaluate liver event etiology (in alignment with the FDA premarketing clinical liver safety guidance). http://www.fda.gov/downloads/Drugs/GuidanceComplianceRegulatoryInformation/Guidances/UCM174090.pdf.

Phase II liver chemistry increased monitoring criteria and required follow up assessments

Liver Chemistry Increased Monitoring Criteria	
**ALT-absolute**	ALT ≥ 5xULN
**ALT Increase**	ALT ≥ 3xULN persists for ≥4 weeks
**Bilirubin[1, 2]**	ALT ≥ 3xULN **and** bilirubin ≥ 2xULN (>35% direct bilirubin)
**INR[2]**	ALT ≥ 3xULN **and** INR>1.5, if INR measured
**Cannot Monitor**	ALT ≥ 3xULN and cannot be monitored weekly for 4 weeks
**Symptomatic[3]**	ALT ≥ 3xULN associated with symptoms (new or worsening) believed to be related to liver injury or hypersensitivity

Required Actions and Follow up Assessments following ANY Liver Event	
**Actions**	**Follow Up Assessments**
• Report the event to GSK **within 24 hours**<br>• Complete the liver event CRF and complete an SAE data collection tool if the event also meets the criteria for an SAE[2]<br>• Perform liver event follow up assessments<br>• Monitor the subject until liver chemistries resolve, stabilize, or return to within baseline (see **MONITORING** below). "Baseline" refers to the laboratory assessments performed closest and prior to dosing of study treatment.<br><br>**MONITORING:**<br>**<u>For bilirubin or INR criteria:</u>**	• Viral hepatitis serology[4]<br>• Blood sample for pharmacokinetic (PK) analysis, obtained within 72 hrs after an identified liver event if within 6 months post dose.[5]<br>• Serum creatine phosphokinase (CPK) and lactate dehydrogenase (LDH).<br>• Fractionate bilirubin, if total bilirubin≥2xULN<br>• Obtain complete blood count with differential to assess eosinophilia<br>• Record the appearance or worsening of clinical symptoms of liver injury, or hypersensitivity, on the AE report form

• Repeat liver chemistries (include ALT, AST, alkaline phosphatase, bilirubin) and perform liver event follow up assessments within **24 hrs**<br>• Monitor subjects twice weekly until liver chemistries resolve, stabilize or return to within baseline<br>• A specialist or hepatology consultation is recommended<br>**For All other criteria:**<br>• Repeat liver chemistries (include ALT, AST, alkaline phosphatase, bilirubin) and perform liver event follow up assessments within **24-72 hrs**<br>• Monitor subjects weekly until liver chemistries resolve, stabilize or return to within baseline	• Record use of concomitant medications on the concomitant medications report form including acetaminophen, herbal remedies, other over the counter medications.<br>• Record alcohol use on the liver event alcohol intake case report form<br>**For bilirubin or INR criteria:**<br>• Anti-nuclear antibody, anti-smooth muscle antibody, Type 1 anti-liver kidney microsomal antibodies, and quantitative total immunoglobulin G (IgG or gamma globulins).<br>• Serum acetaminophen adduct HPLC assay (quantifies potential acetaminophen contribution to liver injury in subjects with definite or likely acetaminophen use in the preceding week [James 2009]).<br>• Liver imaging (ultrasound, magnetic resonance, or computerised tomography) and /or liver biopsy to evaluate liver disease; complete Liver Imaging and/or Liver Biopsy CRF forms.

1. Serum bilirubin fractionation should be performed if testing is available. If serum bilirubin fractionation is not immediately available, discontinue study treatment for that subject if ALT ≥ 3xULN **and** bilirubin ≥ 2xULN.. Additionally, if serum bilirubin fractionation testing is unavailable, **record presence of detectable urinary bilirubin on dipstick**, indicating direct bilirubin elevations and suggesting liver injury.
2. All events of ALT ≥ 3xULN **and** bilirubin ≥ 2xULN (>35% direct bilirubin) or ALT ≥ 3xULN **and** INR>1.5, if INR measured which may indicate severe liver injury (possible 'Hy's Law'), **must be reported as an SAE (excluding studies of hepatic impairment or cirrhosis)**; INR measurement is not required and the threshold value stated will not apply to subjects receiving anticoagulants
3. New or worsening symptoms believed to be related to liver injury (such as fatigue, nausea, vomiting, right upper quadrant pain or tenderness, or jaundice) or believed to be related to hypersensitivity (such as fever, rash or eosinophilia)
4. Includes: Hepatitis A IgM antibody; Hepatitis B surface antigen and Hepatitis B Core Antibody (IgM); Hepatitis C RNA; Cytomegalovirus IgM antibody; Epstein-Barr viral capsid antigen IgM antibody (or if unavailable, obtain heterophile antibody or monospot testing); Hepatitis E IgM antibody
5. Record the date/time of the PK blood sample draw and the date/time of the last dose of study treatment prior to blood sample draw on the CRF. If the date or time of the last dose is unclear, provide the subject's best approximation. If the date/time of the last dose cannot be approximated OR a PK sample cannot be collected in the time period indicated above, do not obtain a PK sample. Instructions for sample handling and shipping are included in the SRM.

References

James LP, Letzig L, Simpson PM, Capparelli E, Roberts DW, Hinson JA, Davern TJ, Lee WM. Pharmacokinetics of Acetaminophen-Adduct in Adults with Acetaminophen Overdose and Acute Liver Failure. Drug Metab Dispos 2009; 37:1779-1784.

12.3. Appendix 3: Definition of and Procedures for Recording, Evaluating, Follow-Up and Reporting of Adverse Events

12.3.1. Definition of Adverse Events

**Adverse Event Definition:**
• An AE is any untoward medical occurrence in a patient or clinical investigation subject, temporally associated with the use of a medicinal product, whether or not considered related to the medicinal product.<br>• NOTE: An AE can therefore be any unfavorable and unintended sign (including an abnormal laboratory finding), symptom, or disease (new or exacerbated) temporally associated with the use of a medicinal product.

**Events meeting AE definition include:**
• Any abnormal laboratory test results (hematology, clinical chemistry, or urinalysis) or other safety assessments (e.g., ECGs, radiological scans, vital signs measurements), including those that worsen from baseline, and felt to be clinically significant in the medical and scientific judgement of the investigator.<br>• Exacerbation of a chronic or intermittent pre-existing condition including either an increase in frequency and/or intensity of the condition.<br>• New conditions detected or diagnosed after study treatment administration even though it may have been present prior to the start of the study.<br>• Signs, symptoms, or the clinical sequelae of a suspected interaction.<br>• Signs, symptoms, or the clinical sequelae of a suspected overdose of either study treatment or a concomitant medication (overdose per se will not be reported as an AE/SAE unless this is an intentional overdose taken with possible suicidal/self-harming intent. This should be reported regardless of sequelae).<br>• "Lack of efficacy" or "failure of expected pharmacological action" per se will not be reported as an AE or SAE. However, the signs and symptoms and/or clinical sequelae resulting from lack of efficacy will be reported if they fulfil the definition of an AE or SAE.

**Events NOT meeting definition of an AE include:**
• Any clinically significant abnormal laboratory findings or other abnormal safety assessments which are associated with the underlying disease, unless judged by the investigator to be more severe than expected for the subject's condition.<br>• The disease/disorder being studied or expected progression, signs, or symptoms of the disease/disorder being studied, unless more severe than expected for the subject's condition.<br>• Medical or surgical procedure (e.g., endoscopy, appendectomy): the condition that

leads to the procedure is an AE.
• Situations where an untoward medical occurrence did not occur (social and/or convenience admission to a hospital).
• Anticipated day-to-day fluctuations of pre-existing disease(s) or condition(s) present or detected at the start of the study that do not worsen.

12.3.2. Definition of Serious Adverse Events

If an event is not an AE per definition above, then it cannot be an SAE even if serious conditions are met (e.g., hospitalization for signs/symptoms of the disease under study, death due to progression of disease, etc).

**Serious Adverse Event (SAE) is defined as any untoward medical occurrence that, at any dose:**
**a. Results in death**
**b. Is life-threatening**<br>NOTE:<br>The term 'life-threatening' in the definition of 'serious' refers to an event in which the subject was at risk of death at the time of the event. It does not refer to an event, which hypothetically might have caused death, if it were more severe.
**c. Requires hospitalization or prolongation of existing hospitalization**<br>NOTE:<br>• In general, hospitalization signifies that the subject has been detained (usually involving at least an overnight stay) at the hospital or emergency ward for observation and/or treatment that would not have been appropriate in the physician's office or out-patient setting. Complications that occur during hospitalization are AEs. If a complication prolongs hospitalization or fulfills any other serious criteria, the event is serious. When in doubt as to whether "hospitalization" occurred or was necessary, the AE should be considered serious.<br>• Hospitalization for elective treatment of a pre-existing condition that did not worsen from baseline is not considered an AE.
**d. Results in disability/incapacity**<br>NOTE:<br>• The term disability means a substantial disruption of a person's ability to conduct normal life functions.<br>• This definition is not intended to include experiences of relatively minor medical significance such as uncomplicated headache, nausea, vomiting, diarrhea, influenza, and accidental trauma (e.g. sprained ankle) which may interfere or prevent everyday

	life functions but do not constitute a substantial disruption
**e.**	**Is a congenital anomaly/birth defect**
**f.**	**Other situations:**<br>• Medical or scientific judgment should be exercised in deciding whether reporting is appropriate in other situations, such as important medical events that may not be immediately life-threatening or result in death or hospitalization but may jeopardize the subject or may require medical or surgical intervention to prevent one of the other outcomes listed in the above definition. These should also be considered serious.<br>• Examples of such events are invasive or malignant cancers, intensive treatment in an emergency room or at home for allergic bronchospasm, blood dyscrasias or convulsions that do not result in hospitalization, or development of drug dependency or drug abuse
**g.**	**Is associated with liver injury <u>and</u> impaired liver function defined as:**<br>• ALT ≥ 3xULN and total bilirubin* ≥ 2xULN (>35% direct), **or**<br>• ALT ≥ 3xULN and INR** > 1.5.<br>* Serum bilirubin fractionation should be performed if testing is available; if unavailable, measure urinary bilirubin via dipstick. If fractionation is unavailable and ALT ≥ 3xULN and total bilirubin ≥ 2xULN, then the event is still to be reported as an SAE.<br>** INR testing not required per protocol and the threshold value does not apply to subjects receiving anticoagulants. If INR measurement is obtained, the value is to be recorded on the SAE form.

12.3.3. Definition of Cardiovascular Events

**Cardiovascular Events (CV) Definition:**
Investigators will be required to fill out the specific CV event page of the CRF for the following AEs and SAEs:<br>• Myocardial infarction/unstable angina<br>• Congestive heart failure<br>• Arrhythmias<br>• Valvulopathy<br>• Pulmonary hypertension<br>• Cerebrovascular events/stroke and transient ischemic attack<br>• Peripheral arterial thromboembolism<br>• Deep venous thrombosis/pulmonary embolism<br>• Revascularization

12.3.4. Recording of AEs and SAEs

AEs and SAE Recording:
• When an AE/SAE occurs, it is the responsibility of the investigator to review all documentation (e.g., hospital progress notes, laboratory, and diagnostics reports) relative to the event.<br>• The investigator will then record all relevant information regarding an AE/SAE in the CRF<br>• It is **not** acceptable for the investigator to send photocopies of the subject's medical records to GSK in lieu of completion of the GSK, AE/SAE CRF page.<br>• There may be instances when copies of medical records for certain cases are requested by GSK. In this instance, all subject identifiers, with the exception of the subject number, will be blinded on the copies of the medical records prior to submission of to GSK.<br>• The investigator will attempt to establish a diagnosis of the event based on signs, symptoms, and/or other clinical information. In such cases, the diagnosis will be documented as the AE/SAE and not the individual signs/symptoms.

12.3.5. Evaluating AEs and SAEs

Assessment of Intensity
The investigator will make an assessment of intensity for each AE and SAE reported during the study and will assign it to one of the following categories:<br>• Mild: An event that is easily tolerated by the subject, causing minimal discomfort and not interfering with everyday activities.<br>• Moderate: An event that is sufficiently discomforting to interfere with normal everyday activities<br>• Severe: An event that prevents normal everyday activities. - an AE that is assessed as severe will not be confused with an SAE. Severity is a category utilized for rating the intensity of an event; and both AEs and SAEs can be assessed as severe.<br>• An event is defined as 'serious' when it meets at least one of the pre-defined outcomes as described in the definition of an SAE.

Assessment of Causality
• The investigator is obligated to assess the relationship between study treatment and the occurrence of each AE/SAE.<br>• A "reasonable possibility" is meant to convey that there are facts/evidence or arguments to suggest a causal relationship, rather than a relationship cannot be ruled

out.

- The investigator will use clinical judgment to determine the relationship.
- Alternative causes, such as natural history of the underlying diseases, concomitant therapy, other risk factors, and the temporal relationship of the event to the study treatment will be considered and investigated.
- The investigator will also consult the Investigator Brochure (IB) and/or Product Information, for marketed products, in the determination of his/her assessment.
- For each AE/SAE the investigator **must** document in the medical notes that he/she has reviewed the AE/SAE and has provided an assessment of causality.
- There may be situations when an SAE has occurred and the investigator has minimal information to include in the initial report to GSK. However, **it is very important that the investigator always make an assessment of causality for every event prior to the initial transmission of the SAE data to GSK.**
- The investigator may change his/her opinion of causality in light of follow-up information, amending the SAE data collection tool accordingly.
- The causality assessment is one of the criteria used when determining regulatory reporting requirements.

Follow-up of AEs and SAEs

- The investigator is obligated to perform or arrange for the conduct of supplemental measurements and/or evaluations as may be indicated or as requested by GSK to elucidate as fully as possible the nature and/or causality of the AE or SAE.
- The investigator is obligated to assist. This may include additional laboratory tests or investigations, histopathological examinations or consultation with other health care professionals.
- If a subject dies during participation in the study or during a recognized follow-up period, the investigator will provide GSK with a copy of any post-mortem findings, including histopathology.
- New or updated information will be recorded in the originally completed CRF.
- The investigator will submit any updated SAE data to GSK within the designated reporting time frames.

12.3.6. Reporting of SAEs to GSK

SAE reporting to GSK via electronic data collection tool

- Primary mechanism for reporting SAEs to GSK will be the electronic data collection tool
- If the electronic system is unavailable for greater than 24 hours, the site will use the

paper SAE data collection tool and fax it to the Medical Monitor.

- Site will enter the serious adverse event data into the electronic system as soon as it becomes available.
- The investigator will be required to confirm review of the SAE causality by ticking the ‘reviewed’ box at the bottom of the eCRF page within 72 hours of submission of the SAE.
- After the study is completed at a given site, the electronic data collection tool (e.g., InForm system) will be taken off-line to prevent the entry of new data or changes to existing data
- If a site receives a report of a new SAE from a study subject or receives updated data on a previously reported SAE after the electronic data collection tool has been taken off-line, the site can report this information on a paper SAE form or to the Medical Monitor by telephone.
- Contacts for SAE receipt can be found at the beginning of this protocol on the Sponsor/Medical Monitor Contact Information page.

12.4. Appendix 4: Modified List of Highly Effective Methods for Avoiding Pregnancy in FRP and Collection of Pregnancy Information

12.4.1. Modified List of Highly Effective Methods for Avoiding Pregnancy in Females of Reproductive Potential (FRP)

This list does not apply to FRP with same sex partners, or for subjects who are and will continue to be abstinent from penile-vaginal intercourse on a long term and persistent basis, when this is their preferred and usual lifestyles. Periodic abstinence (e.g. calendar, ovulation, symptothermal, post-ovulation methods) and withdrawal are not acceptable methods of contraception.

- Contraceptive subdermal implant
- Intrauterine device or intrauterine system
- Combined estrogen and progestogen oral contraceptive[Hatcher, 2011])
- Injectable progestogen [Hatcher, 2011]
- Contraceptive vaginal ring [Hatcher, 2011]
- Percutaneous contraceptive patches [Hatcher, 2011]
- Male partner sterilization with documentation of azoospermia prior to the female subject's entry into the study, and this male is the sole partner for that subject [Hatcher, 2011]. The documentation on male sterility can come from the site personnel’s: review of subject’s medical records, medical examination of the subject and/or semen analysis, or medical history interview provided by her or her partner.

This is an all inclusive list of those methods that meet the GSK definition of highly effective: having a failure rate of less than 1% per year when used consistently and, correctly and, when applicable, in accordance with the product label. For non-product methods (e.g. male sterility), the investigator determines what is consistent and correct use. The GSK definition is based on the definition provided by the ICH [ICH, M3 (R2) 2009].

The investigator is responsible for ensuring that subjects understand how to properly use these methods of contraception by providing counsel directly or by referring subjects to health care professionals with expertise in this area.

12.4.2. Collection of Pregnancy Information

Female Participant:

- Investigator will collect pregnancy information on any female subject, who becomes pregnant while participating in this study
- Information will be recorded on the appropriate form and submitted to GSK within 2 weeks of learning of a subject's pregnancy.

- Subject will be followed to determine the outcome of the pregnancy. The investigator will collect follow up information on mother and infant, which will be forwarded to GSK. Generally, follow-up will not be required for longer than 6 to 8 weeks beyond the estimated delivery date.
- Any termination of pregnancy will be reported, regardless of fetal status (presence or absence of anomalies) or indication for procedure.
- While pregnancy itself is not considered to be an AE or SAE, any pregnancy complication or elective termination of a pregnancy will be reported as an AE or SAE.
- A spontaneous abortion is always considered to be an SAE and will be reported as such.
- Any SAE occurring as a result of a post-study pregnancy which is considered reasonably related to the study treatment by the investigator will be reported to GSK as described in Appendix 4. While the investigator is not obligated to actively seek this information in former study participants, he or she may learn of an SAE through spontaneous reporting.

Female Partner of Male Participant:

- Investigator will attempt to collect pregnancy information on any female partner of a male study subject who becomes pregnant while participating in this study. This applies only to subjects who are randomized to receive study medication.
- After obtaining the necessary signed informed consent from the female partner directly, the investigator will record pregnancy information on the appropriate form and submit it to GSK within 2 weeks of learning of the partner's pregnancy
- Partner will also be followed to determine the outcome of the pregnancy. Information on the status of the mother and child will be forwarded to GSK.
- Generally, follow-up will be no longer than 6 to 8 weeks following the estimated delivery date. Any termination of the pregnancy will be reported regardless of fetal status (presence or absence of anomalies) or indication for procedure.

References

Hatcher RA, Trussell J, Nelson AL, Cates W Jr, Stewart F, Kowal D, Policar MS, editors. Contraceptive Technology. 20th edition. Atlanta, Georgia: Ardent Media, Inc., 2011: 50. Table 3 2.

International Conference on Harmonization of Technical Requirements for Registration of Pharmaceuticals For Human Use. ICH Harmonized Tripartite Guideline. Nonclinical Safety Studies for the Conduct of Human Clinical Trials and Marketing Authorization for Pharmaceuticals. ICH M3 (R2). 2009.
